# Supplementary material for: Integrative Single-Cell RNA-Seq and ATAC-Seq Analysis of Human Developmental Hematopoiesis
Source: Cell Stem Cell. 2021 Mar 4;28(3):472–487.e7. doi: 10.1016/j.stem.2020.11.015 (PMC7939551; doi:10.1016/j.stem.2020.11.015)
Supplement: Document S1. Figures S1–S7 and Tables S1–S3 [file mmc1.pdf]

Cell Stem Cell, Volume *28*

Supplemental Information

Integrative Single-Cell RNA-Seq and ATAC-Seq Analysis of Human Developmental Hematopoiesis

Anna Maria Ranzoni, Andrea Tangherloni, Ivan Berest, Simone Giovanni Riva, Brynelle Myers, Paulina M. Strzelecka, Jiarui Xu, Elisa Panada, Irina Mohorianu, Judith B. Zaugg, and Ana Cvejic

Integrative Single-cell RNA-Seq and ATAC-Seq Analysis of Human Developmental Haematopoiesis

Anna Maria Ranzoni, Andrea Tangherloni, Ivan Berest,
Simone Giovanni Riva, Brynelle Myers, Paulina M. Strzelecka,
Jiarui Xu, Elisa Panada, Irina Mohorianu,
Judith B. Zaugg, Ana Cvejic

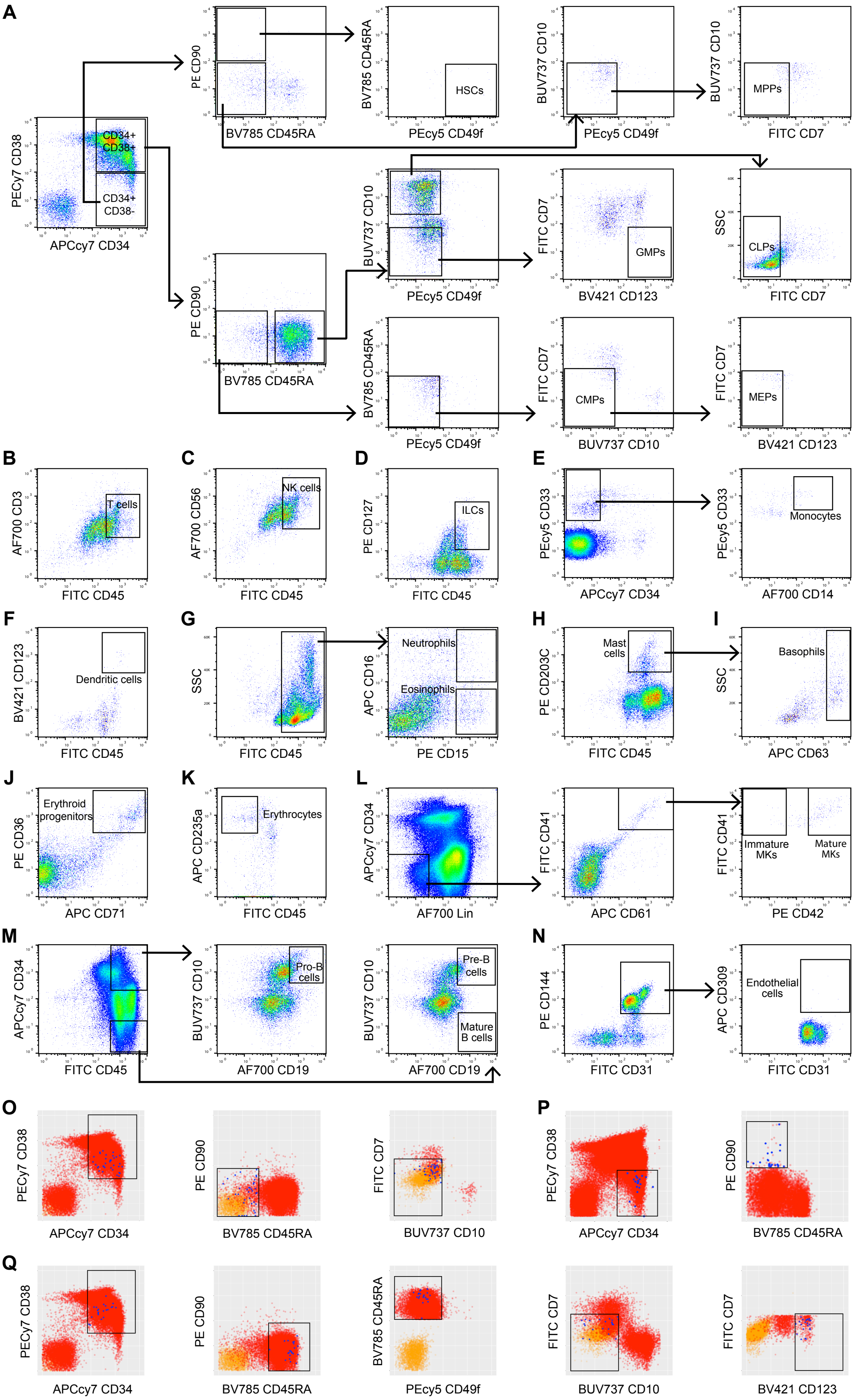

A
PE CD90
BV785 CD45RA
HSCs
PEcy5 CD49f
BUV737 CD10
MPPs
FITC CD7
PECy7 CD38
CD34+ CD38+
CD34+ CD38-
APCcy7 CD34
GMPs
BV421 CD123
SSC
CLPs
CMPs
MEPs
B
AF700 CD3
T cells
FITC CD45
C
AF700 CD56
NK cells
D
PE CD127
ILCs
E
PEcy5 CD33
Monocytes
AF700 CD14
F
BV421 CD123
Dendritic cells
G
Neutrophils
Eosinophils
APC CD16
PE CD15
H
PE CD203C
Mast cells
I
Basophils
APC CD63
J
PE CD36
Erythroid progenitors
APC CD71
K
APC CD235a
Erythrocytes
L
APCcy7 CD34
AF700 Lin
FITC CD41
APC CD61
Immature MKs
Mature MKs
PE CD42
M
Pro-B cells
Pre-B cells
Mature B cells
AF700 CD19
N
PE CD144
FITC CD31
APC CD309
Endothelial cells
O
P
Q

Supplementary figure 1 – Sorting panels, Related to Figure 1, related to Figure 1. A-N. FACS sorting panel and gating strategy for the isolation of phenotypically defined cell types: **A.** Committed and non-committed haematopoietic progenitors, HSCs, MPPs, CMPs, GMPs, MEPs, and CLPs. **B.** T cells. **C.** NK cells. **D.** ILCs. **E.** Monocytes. **F.** Dendritic cells. **G.** Neutrophils and eosinophils. **H.** Mast cells. **I.** Basophils. **J.** Erythroid progenitors. **K.** Erythrocytes. **L.** Immature and mature MKs. **M.** Pro-B cells, pre-B cells, and mature B cells. **N.** Endothelial cells. **O-Q.** Index sorting data FACS plots showing sorted cells (blue), total gated population (red), and unstained population (yellow) for defined cell types: **O.** CMPs. **P.** HSCs. **Q.** GMPs. HSCs - haematopoietic stem cells, MPPs - multipotent progenitors, CMPs - common myeloid progenitors, GMPs - granulocyte-monocyte progenitors, MEPs - megakaryocyte-erythroid progenitors and CLPs - common lymphoid progenitors, NK cells - natural killer cells, ILCs - innate lymphoid cells, MKs - megakaryocytes.

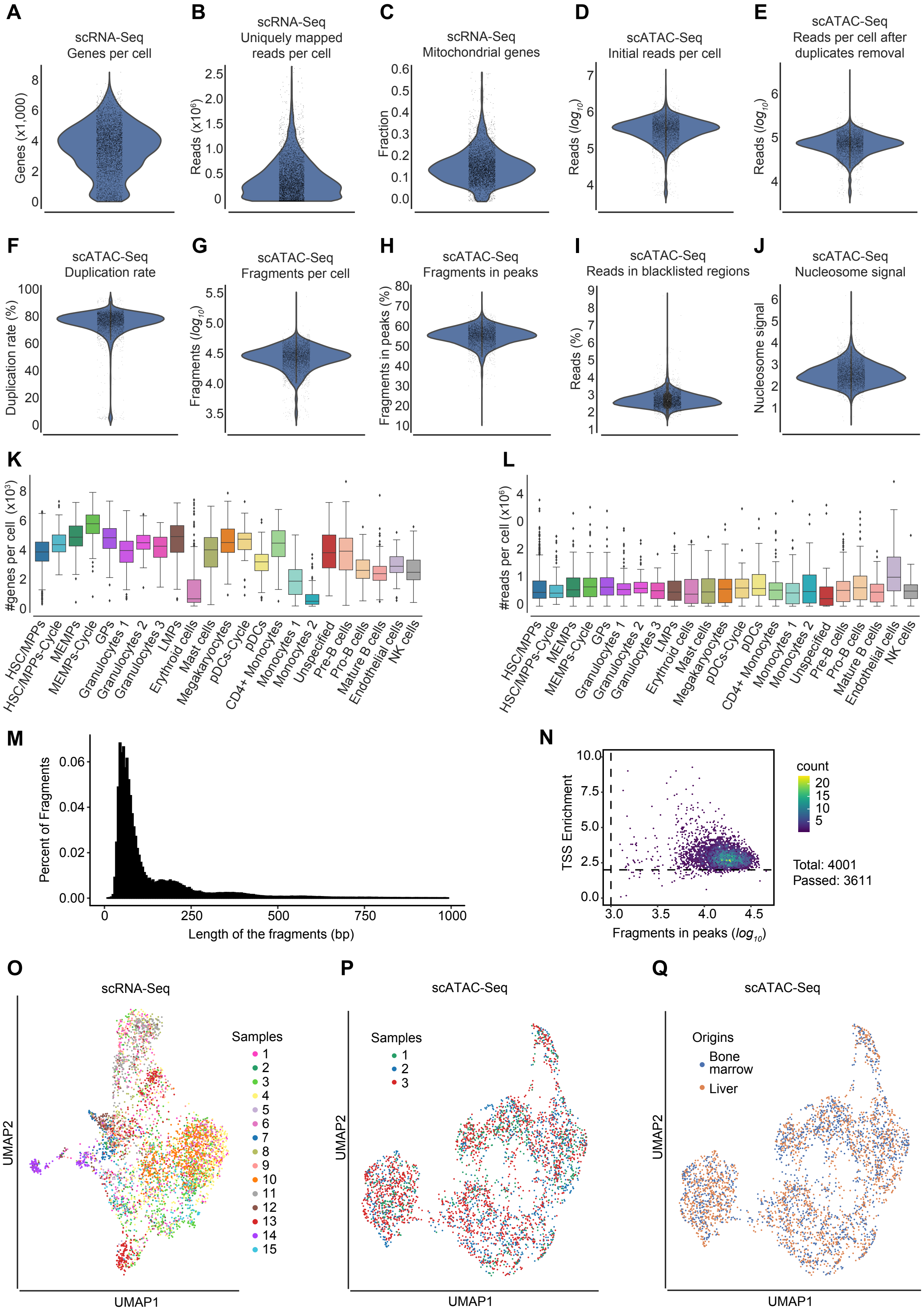
A
scRNA-Seq
Genes per cell
Genes (x1,000)
B
scRNA-Seq
Uniquely mapped
reads per cell
Reads (x10⁶)
C
scRNA-Seq
Mitochondrial genes
Fraction
D
scATAC-Seq
Initial reads per cell
Reads (log10)
E
scATAC-Seq
Reads per cell after
duplicates removal
Reads (log10)
F
scATAC-Seq
Duplication rate
Duplication rate (%)
G
scATAC-Seq
Fragments per cell
Fragments (log10)
H
scATAC-Seq
Fragments in peaks
Fragments in peaks (%)
I
scATAC-Seq
Reads in blacklisted regions
Reads (%)
J
scATAC-Seq
Nucleosome signal
Nucleosome signal
K
#genes per cell (x10³)
L
#reads per cell (x10⁶)
HSC/MPPs
HSC/MPPs-Cycle
MEMPs
MEMPs-Cycle
GPs
Granulocytes 1
Granulocytes 2
Granulocytes 3
LMPs
Erythroid cells
Mast cells
Megakaryocytes
pDCs-Cycle
pDCs
CD4+ Monocytes
Monocytes 1
Monocytes 2
Unspecified
Pre-B cells
Pro-B cells
Mature B cells
Endothelial cells
NK cells
M
Percent of Fragments
Length of the fragments (bp)
N
TSS Enrichment
Fragments in peaks (log10)
count
Total: 4001
Passed: 3611
O
scRNA-Seq
Samples
UMAP1
UMAP2
P
scATAC-Seq
Samples
UMAP1
UMAP2
Q
scATAC-Seq
Origins
Bone marrow
Liver
UMAP1
UMAP2

Supplementary figure 2 – Quality control and batch effects correction in scRNA-Seq and scATAC-Seq data, related to Figures 1 and 3. A. Violin plots showing the number of expressed genes per cell in scRNA-Seq data. **B.** Violin plots showing the number of uniquely mapped reads against the reference genome per cell in scRNA-Seq data. **C.** Violin plots showing the fraction of mitochondrial genes compared to all genes per cell in scRNA-Seq data. **D.** Violin plots showing the number of reads per cell, prior to duplicates removal, in scATAC-Seq data. The y-axis is in log_{10} scale. **E.** Violin plots showing the number of reads per cell after duplicates removal in scATAC-Seq data. The y-axis is in log_{10} scale. **F.** Violin plots showing the duplicate rate in scATAC-Seq data. **G.** Violin plots showing the number of fragments per cell in scATAC-Seq data. The y-axis is in log_{10} scale. **H.** Violin plots showing the percentage of fragments per peak in scATAC-Seq data. **I.** Violin plots showing the percentage of reads mapping to the blacklist regions in scATAC-Seq data. **J.** Violin plots showing the nucleosome signal per cell in scATAC-Seq data. **K.** Box plot showing the number of genes per cell in each identified cell type in scRNA-Seq data. **L.** Box plot showing the number of uniquely mapped reads per cell in each identified cell type in scRNA-Seq data. M. Histogram showing the length of the fragments in terms of base pairs (200 bins). **N.** Scatterplot showing the fragments in peaks with respect to TSS enrichment. The colour intensity represents the number of counts. The x-axis is in log_{10} scale. **O.** UMAP visualization of the scRNA-Seq samples ($n = 15$) after the batch effect correction with BBKNN. Each colour represents a different sample. **P.** UMAP visualization of the scATAC-Seq samples ($n = 3$) after the batch effect correction with Harmony. Each colour represents a different sample. **Q.** UMAP visualization of the scATAC-Seq bone marrow (blue) and liver (orange) CD34+ CD38- cells after the batch effect correction with Harmony.

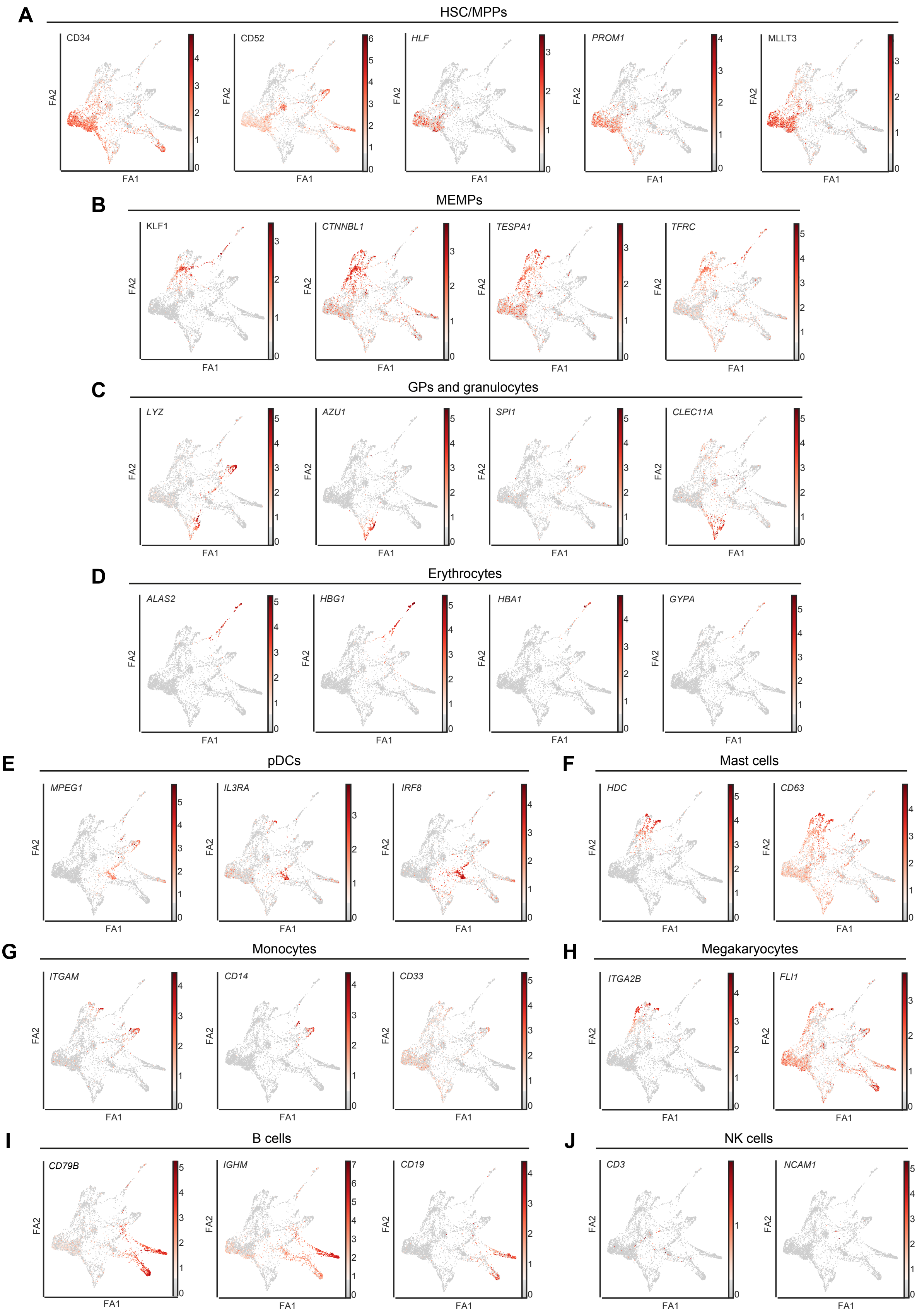
A
HSC/MPPs
CD34
CD52
HLF
PROM1
MLLT3
B
MEMPs
KLF1
CTNNBL1
TESPA1
TFRC
C
GPs and granulocytes
LYZ
AZU1
SPI1
CLEC11A
D
Erythrocytes
ALAS2
HBG1
HBA1
GYPA
E
pDCs
MPEG1
IL3RA
IRF8
F
Mast cells
HDC
CD63
G
Monocytes
ITGAM
CD14
CD33
H
Megakaryocytes
ITGA2B
FLI1
I
B cells
CD79B
IGHM
CD19
J
NK cells
CD3
NCAM1
FA1
FA2

Supplementary figure 3 – Expression of top marker genes along the differentiation trajectory, related to Figure 2. (A-J) FDG visualisation of the log-normalised gene expression of marker genes along the differentiation trajectory. **A.** HSC/MPPs (*CD34*, *CD52*, *HLF*, *PROM1*, and *MLLT3*). **B.** MEMPs (*KLF1*, *CTNNBL1*, *TESPA1*, and *TFRC*). **C.** GPs and granulocytes (*LYZ*, *AZU1*, *SPI1*, and *CLEC11A*). **D.** Erythrocytes (*ALAS2*, *HBG1*, *HBA1*, and *GYPA*). **E.** pDCs (*MPEG1*, *IL3RA*, and *IRF8*). **F.** Mast cells (*HDC* and *CD63*). **G.** Monocytes (*ITGAM*, *CD14*, and *CD33*). **H.** Megakaryocytes (*ITGA2B* and *FLI1*). **I.** B cells (*CD79B*, *IGHM*, and *CD19*). **J.** NK cells (*CD3* and *NCAM1*). Force-Directed Graph - FDG; ForceAtlas2 - FA2; HSC/MPPs - haematopoietic stem cells/multipotent progenitors; MEMPs - megakaryocyte-erythroid-mast progenitors; GPs - granulocytic progenitors; pDCs-Cycle - cycling plasmacytoid dendritic cells.

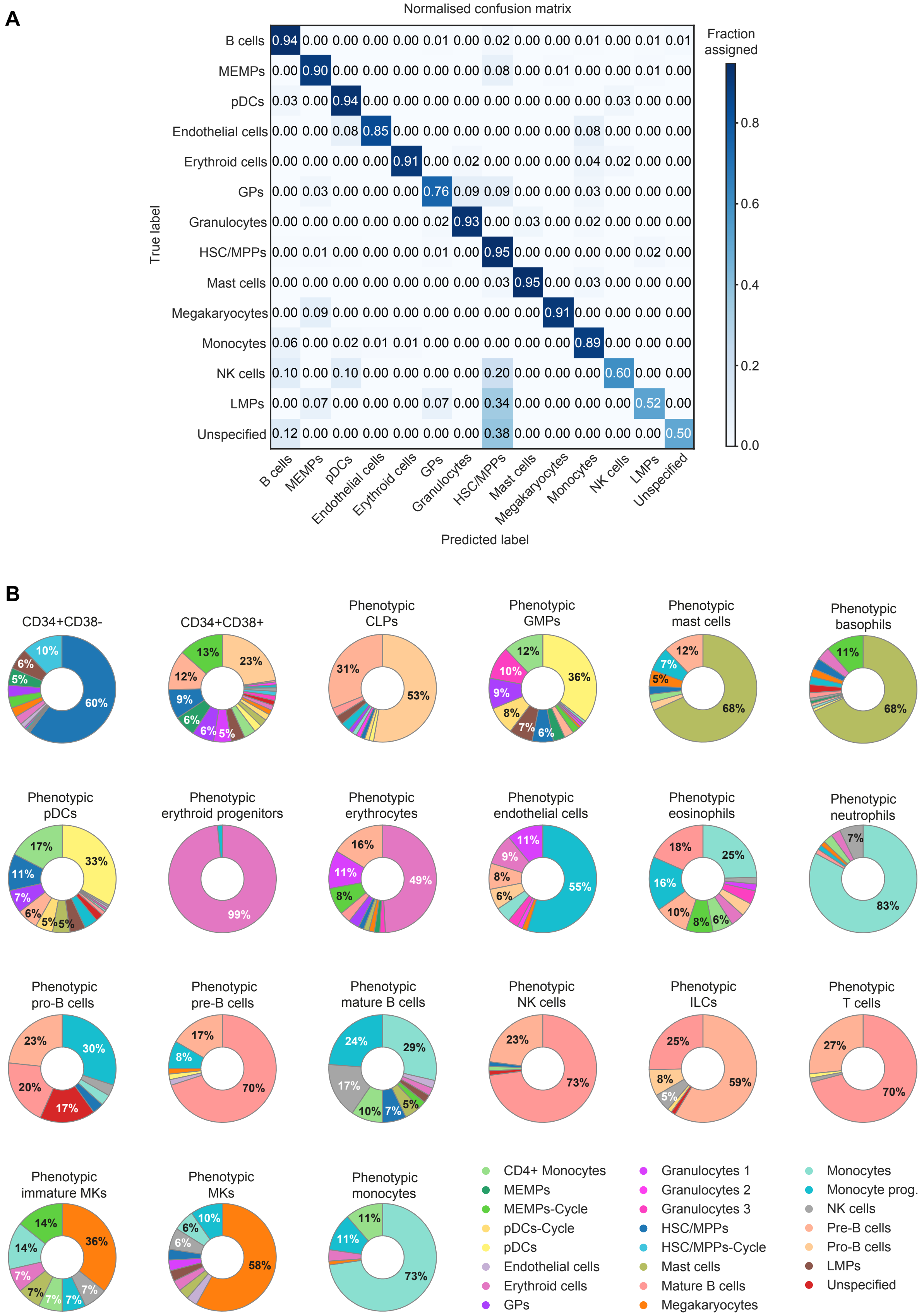
A
Normalised confusion matrix
True label
Predicted label
Fraction assigned
0.0
0.2
0.4
0.6
0.8
B cells 0.94 0.00 0.00 0.00 0.00 0.01 0.00 0.02 0.00 0.00 0.01 0.00 0.01 0.01
MEMPs 0.00 0.90 0.00 0.00 0.00 0.00 0.00 0.08 0.00 0.01 0.00 0.00 0.01 0.00
pDCs 0.03 0.00 0.94 0.00 0.00 0.00 0.00 0.00 0.00 0.00 0.00 0.03 0.00 0.00
Endothelial cells 0.00 0.00 0.08 0.85 0.00 0.00 0.00 0.00 0.00 0.00 0.08 0.00 0.00 0.00
Erythroid cells 0.00 0.00 0.00 0.00 0.91 0.00 0.02 0.00 0.00 0.00 0.04 0.02 0.00 0.00
GPs 0.00 0.03 0.00 0.00 0.00 0.76 0.09 0.09 0.00 0.00 0.03 0.00 0.00 0.00
Granulocytes 0.00 0.00 0.00 0.00 0.00 0.02 0.93 0.00 0.03 0.00 0.02 0.00 0.00 0.00
HSC/MPPs 0.00 0.01 0.00 0.00 0.00 0.01 0.00 0.95 0.00 0.00 0.00 0.00 0.02 0.00
Mast cells 0.00 0.00 0.00 0.00 0.00 0.00 0.00 0.03 0.95 0.00 0.03 0.00 0.00 0.00
Megakaryocytes 0.00 0.09 0.00 0.00 0.00 0.00 0.00 0.00 0.00 0.91 0.00 0.00 0.00 0.00
Monocytes 0.06 0.00 0.02 0.01 0.01 0.00 0.00 0.00 0.00 0.00 0.89 0.00 0.00 0.00
NK cells 0.10 0.00 0.10 0.00 0.00 0.00 0.00 0.20 0.00 0.00 0.00 0.60 0.00 0.00
LMPs 0.00 0.07 0.00 0.00 0.00 0.07 0.00 0.34 0.00 0.00 0.00 0.00 0.52 0.00
Unspecified 0.12 0.00 0.00 0.00 0.00 0.00 0.00 0.38 0.00 0.00 0.00 0.00 0.00 0.50
B cells
MEMPs
pDCs
Endothelial cells
Erythroid cells
GPs
Granulocytes
HSC/MPPs
Mast cells
Megakaryocytes
Monocytes
NK cells
LMPs
Unspecified
B
CD34+CD38-
CD34+CD38+
Phenotypic CLPs
Phenotypic GMPs
Phenotypic mast cells
Phenotypic basophils
Phenotypic pDCs
Phenotypic erythroid progenitors
Phenotypic erythrocytes
Phenotypic endothelial cells
Phenotypic eosinophils
Phenotypic neutrophils
Phenotypic pro-B cells
Phenotypic pre-B cells
Phenotypic mature B cells
Phenotypic NK cells
Phenotypic ILCs
Phenotypic T cells
Phenotypic immature MKs
Phenotypic MKs
Phenotypic monocytes
CD4+ Monocytes
MEMPs
MEMPs-Cycle
pDCs-Cycle
pDCs
Endothelial cells
Erythroid cells
GPs
Granulocytes 1
Granulocytes 2
Granulocytes 3
HSC/MPPs
HSC/MPPs-Cycle
Mast cells
Mature B cells
Megakaryocytes
Monocytes
Monocyte prog.
NK cells
Pre-B cells
Pro-B cells
LMPs
Unspecified

Supplementary figure 4 – Validation of the cell type assignment and transcriptional heterogeneity of phenotypically defined cell populations, related to Figures 1 and 3. A. Confusion matrix showing the cell type assignment achieved by the DNN on the test set (901 cells), considering the top 30 marker genes per cell type (14 distinct cell types). The colour intensity represents the fraction of the assigned cells per cell type. **B.** Donut plots showing the percentage of transcriptionally defined (i.e., manually curated) cell populations in each of the phenotypically defined populations (Expanded from Figure 1C). Each colour represents a different cell type. HSC/MPPs-Cycle - cycling haematopoietic stem cells/multipotent progenitors; HSC/MPPs - haematopoietic stem cells/multipotent progenitors; MEMPs - megakaryocyte-erythroid-mast progenitors; MEMPs-Cycle - cycling megakaryocyte-erythroid-mast progenitors; GPs - granulocytic progenitors; LMPs - lympho-myeloid progenitors; pDCs-Cycle - cycling plasmacytoid dendritic cells; pDCs - plasmacytoid dendritic cells.

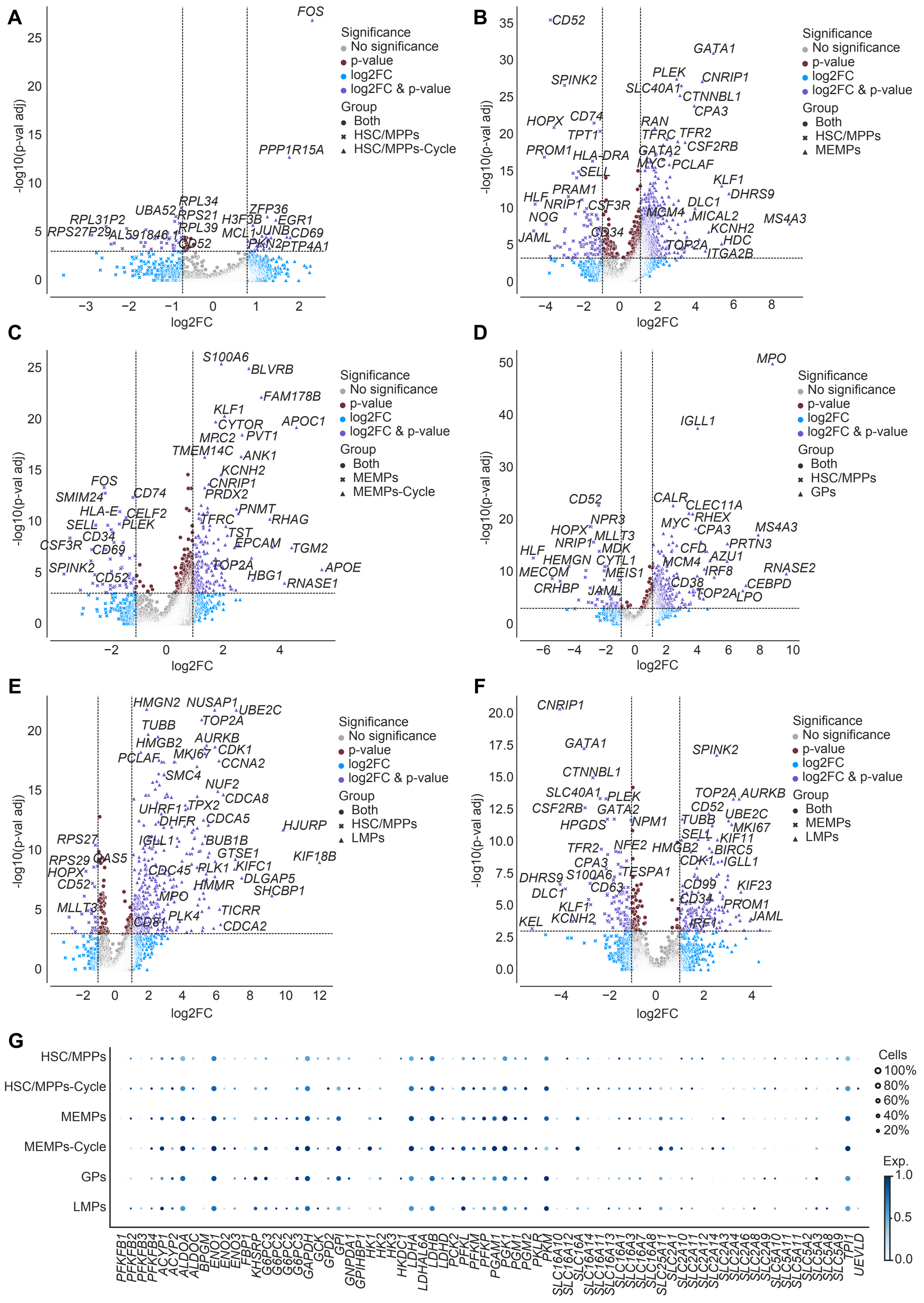

A
FOS
PPP1R15A
RPL34
RPS21
RPL39
UBA52
RPL31P2
RPS27
P29
AL591846.1
CD52
H3F3B
ZFP36
EGR1
MCL1
JUNB
CD69
PKN2
PTP4A1
-log10(p-val adj)
log2FC
Significance
No significance
p-value
log2FC
log2FC & p-value
Group
Both
HSC/MPPs
HSC/MPPs-Cycle
B
CD52
SPINK2
HOPX
CD74
TPT1
PROM1
HLA-DRA
SELL
PRAM1
HLF
NRIP1
CSF3R
NOG
JAML
CD34
GATA1
PLEK
CNRIP1
SLC40A1
CTNNBL1
CPA3
RAN
TFRC
TFR2
GATA2
CSF2RB
MYC
PCLAF
KLF1
DHRS9
DLC1
MCM4
MICAL2
MS4A3
KCNH2
HDC
TOP2A
ITGA2B
-log10(p-val adj)
log2FC
Significance
No significance
p-value
log2FC
log2FC & p-value
Group
Both
HSC/MPPs
MEMPs
C
S100A6
BLVRB
FAM178B
KLF1
CYTOR
APOC1
MPC2
PVT1
TMEM14C
ANK1
KCNH2
CNRIP1
PRDX2
FOS
SMIM24
CD74
HLA-E
CELF2
SELL
PLEK
CD34
CSF3R
CD69
PNMT
RHAG
TFRC
TST
EPCAM
TGM2
SPINK2
CD52
TOP2A
HBG1
APOE
RNASE1
-log10(p-val adj)
log2FC
Significance
No significance
p-value
log2FC
log2FC & p-value
Group
Both
MEMPs
MEMPs-Cycle
D
MPO
IGLL1
CD52
NPR3
HOPX
NRIP1
MLLT3
HLF
MDK
HEMGN
CYTL1
MECOM
MEIS1
CRHBP
JAML
CALR
CLEC11A
RHEX
MYC
CPA3
MS4A3
CFD
PRTN3
AZU1
MCM4
IRF8
RNASE2
CD38
CEBPD
TOP2A
LPO
-log10(p-val adj)
log2FC
Significance
No significance
p-value
log2FC
log2FC & p-value
Group
Both
HSC/MPPs
GPs
E
HMGN2
NUSAP1
UBE2C
TUBB
TOP2A
HMGB2
AURKB
MKI67
CDK1
PCLAF
CCNA2
SMC4
NUF2
CDCA8
UHRF1
TPX2
CDCA5
DHFR
HJURP
RPS27
IGLL1
BUB1B
RPS29
CASP5
GTSE1
KIF18B
HOPX
CDC45
PLK1
KIFC1
CD52
DLGAP5
HMMR
SHCBP1
MPO
MLLT3
TICRR
PLK4
CD81
CDCA2
-log10(p-val adj)
log2FC
Significance
No significance
p-value
log2FC
log2FC & p-value
Group
Both
HSC/MPPs
LMPs
F
CNRIP1
GATA1
SPINK2
CTNNBL1
SLC40A1
PLEK
TOP2A
AURKB
CSF2RB
GATA2
CD52
UBE2C
HPGDS
NPM1
TUBB
MKI67
SELL
KIF11
TFR2
NFE2
HMGB2
BIRC5
CPA3
CDK1
IGLL1
DHRS9
S100A6
TESPA1
DLC1
CD63
CD99
KIF23
CD34
KLF1
PROM1
KEL
KCNH2
IRF1
JAML
-log10(p-val adj)
log2FC
Significance
No significance
p-value
log2FC
log2FC & p-value
Group
Both
MEMPs
LMPs
G
HSC/MPPs
HSC/MPPs-Cycle
MEMPs
MEMPs-Cycle
GPs
LMPs
Cells
100%
80%
60%
40%
20%
Exp.
1.0
0.5
0.0
PFKFB1
PFKFB2
PFKFB3
PFKFB4
ACYP1
ACYP2
ALDOA
ALDOC
BPGM
ENO1
ENO2
ENO3
FBP1
KHSRP
G6PC2
G6PC3
G6PC3
GAPDH
GCK
GPD2
GPI
GNPDA1
GPIHBP1
HK1
HK2
HK3
HKDC1
LDHA
LDHAL6A
LDHB
LDHD
PCK2
PFKL
PFKM
PFKP
PGAM1
PGK1
PGM1
PGM2
PKLR
PKM
SLC16A10
SLC16A12
SLC16A14
SLC16A13
SLC16A1
SLC16A3
SLC16A7
SLC16A8
SLC2A1
SLC25A11
SLC2A10
SLC2A11
SLC2A12
SLC2A14
SLC2A3
SLC2A4
SLC2A6
SLC2A8
SLC2A9
SLC5A10
SLC5A11
SLC5A12
SLC5A3
SLC5A8
SLC5A9
TPI1
UEVLD

Supplementary figure 5 – Differential expression analysis of the progenitor compartment, related to the STAR Methods section. (A-F) Volcano plot showing DEGs between two cell types of interest. **A.** HSC/MPPs and HSC/MPPs-Cycle. **B.** HSC/MPPs and MEMPs. **C.** MEMPs and MEMPs-Cycle. **D.** HSC/MPPs and GPs. **E.** HSC/MPPs and LMPs. **F.** MEMPs and LMPs. The x-axes show the log_2 fold-change (magnitude of change), while the y-axes show the $-log_{10}$ adjusted p-value (statistical significance). We used the Wilcoxon rank-sum with the Benjamini-Hochberg correction. Colours represent the significance of the genes, both in terms of p-value and log_2 fold-change. **G.** Dot plot of the expression of metabolic genes involved in glycolysis in the identified progenitor compartment. The expression of the genes is standardised between 0 and 1. For each gene, the minimum value is subtracted and the result is divided by the maximum. The spot size indicates the percentage of cells that express the gene of interest within each cell type. The colour intensity represents the standardised expression level. HSC/MPPs-Cycle - cycling haematopoietic stem cells/multipotent progenitors; HSC/MPPs - haematopoietic stem cells/multipotent progenitors; MEMPs - megakaryocyte-erythroid-mast progenitors; MEMPs-Cycle - cycling megakaryocyte-erythroid-mast progenitors; GPs - granulocytic progenitors; LMPs - lympho-myeloid progenitors.

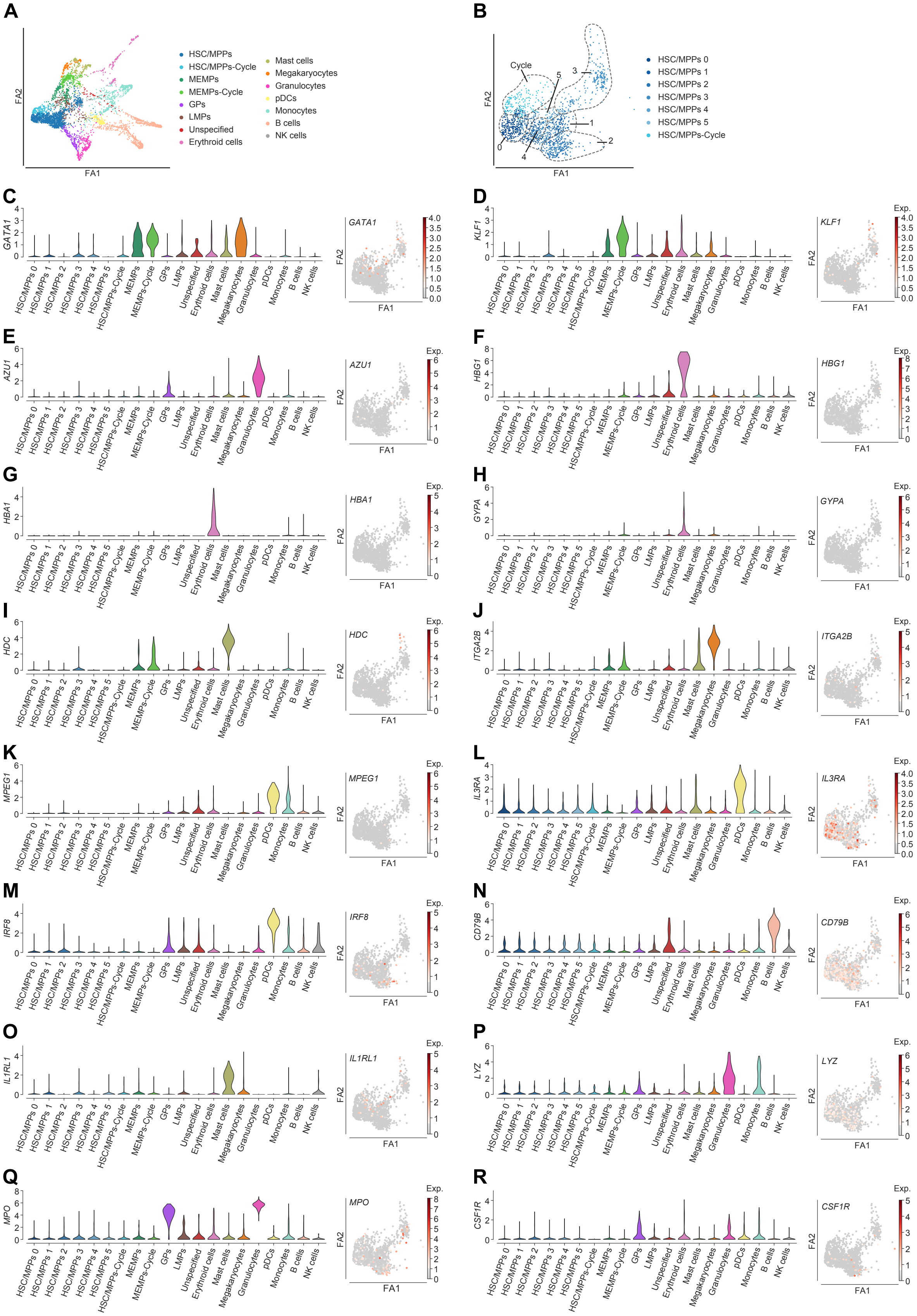
A
FA2
FA1
HSC/MPPs
HSC/MPPs-Cycle
MEMPs
MEMPs-Cycle
GPs
LMPs
Unspecified
Erythroid cells
Mast cells
Megakaryocytes
Granulocytes
pDCs
Monocytes
B cells
NK cells
B
Cycle
HSC/MPPs 0
HSC/MPPs 1
HSC/MPPs 2
HSC/MPPs 3
HSC/MPPs 4
HSC/MPPs 5
HSC/MPPs-Cycle
C
GATA1
D
KLF1
E
AZU1
F
HBG1
G
HBA1
H
GYPA
I
HDC
J
ITGA2B
K
MPEG1
L
IL3RA
M
IRF8
N
CD79B
O
IL1RL1
P
LYZ
Q
MPO
R
CSF1R
Exp.

Supplementary figure 6 – Expression of lineage-specific marker genes in HSC/MPP sub-populations, related to 2. A. FDG visualisation of the identified differentiation trajectory. **B.** FDG visualisation of the HSC/MPP sub-populations. **(C-R)** Left panels - violin plots showing the log-normalised median gene expression of lineage-specific marker genes in the HSC/MPP sub-populations and more differentiated haematopoietic cells. Right panels - FDG visualisations of the log-normalised gene expression of the same genes as in violin plots along the differentiation trajectory, considering only the HSC/MPP sub-populations. **C.** *GATA1*. **D.** *KLF1*. **E.** *AZU1*. **F.** *HBG1*. **G.** *HBA1*. **H.** *GYPA*. **I.** *HDC*. **J.** *ITGA2B*. **K.** *MPEG1*. **L.** *IL3RA*. **M.** *IRF8*. **N.** *CD79B*. **O.** *IL1RL1*. **P.** *LYZ*. **Q.** *MPO*. **R.** *CSF1R*. Force-Directed Graph - FDG; ForceAtlas2 - FA2; HSC/MPPs - haematopoietic stem cells/multipotent progenitors; HSC/MPPs-Cycle - cycling haematopoietic stem cells/multipotent progenitors; MEMPs - megakaryocyte-erythroid-mast progenitors; MEMPs-Cycle - cycling megakaryocyte-erythroid-mast progenitors; GPs - granulocytic progenitors; LMPs - lympho-myeloid progenitors; pDCs - plasmacytoid dendritic cells.

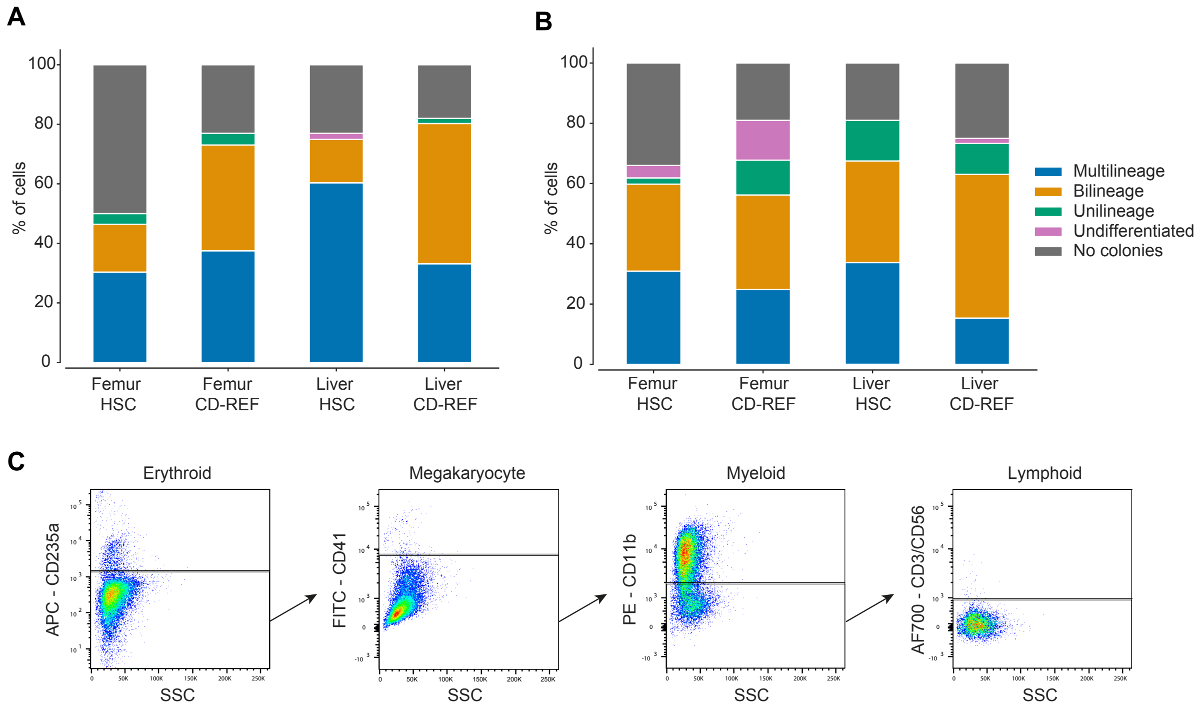

Supplementary figure 7 – Colony formation and the lineage output of CD-REF cells and phenotypic HSCs isolated from the foetal liver and femur, related to Figure 6. A-B. Stacked bar chart showing the differentiation potential and efficiency of colony formation of CD-REF cells compared to the phenotypic HSCs isolated from foetal liver and femur. The y-axis shows the percentage of colonies. The colonies have been divided by their differentiation potential as determined by FACS, namely: multilineage, bilineage, unilineage, and undifferentiated. Data from two different experiments are shown. **C.** Representative FACS plot showing the lineage composition of a quadrilineage colony.

Supplementary table 1 – Cell-surface markers used to isolate cell types, related to Figure 1.

Phenotypic cell type	Cell-surface markers
HSCs	Lin- CD34+ CD38- CD45RA- CD90+ CD49f+/-
MPPs	Lin- CD34+ CD38- CD90- CD45RA- CD49f- CD10- CD7-
CMPs	Lin- CD34+ CD38+ CD90- CD45RA- CD49f- CD10- CD7-
MEPs	Lin- CD34+ CD38+ CD90- CD45RA- CD49f- CD10- CD7- CD123-
GMPs	Lin- CD34+ CD38+ CD90- CD45RA+ CD49f- CD10- CD7- CD123+/-
CLPs	Lin-, CD34+ CD38+ CD90- CD45RA+ CD49f - CD10+ CD7-
T cells	CD45+ CD3+
NK cells	CD45+ CD56+
ILCs	Lin+/- CD45+ CD127+
Monocytes	CD34- CD33+ CD14+
Dendritic cells	Lin- CD45+ CD123+
Mast cells	CD45+ CD203c+
Basophils	CD45+ CD203c+ CD63+
Neutrophils	CD45+ CD15+ CD16+
Eosinophils	CD45+ CD15+ CD16-
Erythroid progenitors	CD36+ CD71+
Erythrocytes	CD235a+
Immature MKs	Lin- CD34- CD41+ CD61+ CD42-
Mature MKs	Lin- CD34- CD41+ CD61+ CD42+
Pro-B cells	CD45+ CD34+ CD19+ CD10+
Pre-B cells	CD45+ CD34- CD19+ CD10+
Mature B cells	CD45+ CD34- CD19+ CD10-
Endothelial cells	CD31+ CD144+ CD309+

Supplementary table 2 – Phenotypic cell types per organ, related to Figure 1.

Phenotypic cell type	Femur	Hip	Liver	Total
Committed progenitors (Lin-CD34+CD38+)	291	128	257	676
Non-committed progenitors (Lin-CD34+CD38-)	262	114	187	563
Basophils	76	0	47	123
CLPs	33	0	41	74
CMPs	130	12	76	218
CD62L-CD52-CD114-CD125-CD117+	79	0	86	165
Dendritic cells	85	0	87	172
Erythroid progenitors	126	0	0	126
Endothelial cells	35	0	29	64
Eosinophils	20	0	29	49
Erythrocytes	22	0	39	61
GMPs	117	7	88	212
HSCs	50	8	67	125
ILCs	75	0	83	158
MEPs	131	22	95	248
Immature MKs	48	0	0	48
Mature MKs	31	0	0	31
Monocytes	45	0	43	88
MPPs	121	38	111	270
Bone marrow cells	137	0	0	137
Mast cells	41	0	0	41
Neutrophils	39	0	31	70
NK-cell	74	0	0	74
CD-REF cells	500	0	0	500
T cells	79	0	0	79
Mature B cells	20	0	22	42
Pre-B cells	34	0	26	60
Pro-B cells	26	0	4	30
Total	2727	329	1448	4504

Supplementary table 3 – scRNA-Seq samples, related to the STAR Methods section.

No.	Gates	Min #counts	Max #counts	Min #genes	Max #genes	Max %mito
1	Non-committed progenitors<br>Committed progenitors	10,000	1,750,000	200	7,500	40
2	Non-committed progenitors<br>Committed progenitors<br>HSCs	10,000	2,000,000	200	7,500	40
3	Non-committed progenitors<br>Committed progenitors<br>HSCs<br>MPPs<br>CMPs<br>GMPs<br>MEPs	10,000	1,500,000	200	7,500	40
4	Non-committed progenitors<br>Committed progenitors<br>HSCs<br>MPPs<br>CMPs<br>GMPs<br>MEPs	10,000	2,750,000	200	9,000	40
5	Immature MKs<br>Mature MKs	10,000	1,750,000	200	9,000	40
6	Non-committed progenitors<br>Committed progenitors<br>HSCs<br>MPPs<br>CMPs<br>GMPs<br>MEPs	10,000	1,500,000	350	7,500	40
7	Bone marrow cells	400	1,750,000	200	7,500	60
8	Endothelial cells<br>Pro-B cells<br>Pre-B cells<br>Mature B	400	1,500,000	200	7,500	60
9	CLPs<br>GMPs<br>Monocytes	1,000	2,000,000	200	7,500	40
10	CD-REF cells	1,000	550,000	200	6,000	40
11	ILCs<br>NK cells<br>T cells	2,000	1,500,000	200	10,000	40
12	Neutrophils<br>Eosinophils<br>Erythrocytes	2,000	1,500,000	200	6,000	40
13	Dendritic cells<br>Mast cells<br>Basophils	2,000	1,500,000	200	7,500	40
14	Erythroid progenitors	500	3,000,000	100	4,500	40
15	CD62L- CD52- CD114-<br>CD125- CD117+	2,000	1,000,000	1,200	7,000	40
