# Supplementary material for: Integrative Single-Cell RNA-Seq and ATAC-Seq Analysis of Human Developmental Hematopoiesis
Source: Cell Stem Cell. 2021 Mar 4;28(3):472–487.e7. doi: 10.1016/j.stem.2020.11.015 (PMC7939551; doi:10.1016/j.stem.2020.11.015)
Supplement: Document S2. Article plus Supplemental Information [file mmc2.pdf]

Cell Stem Cell

Article

Integrative Single-Cell RNA-Seq and ATAC-Seq Analysis of Human Developmental Hematopoiesis

Graphical Abstract

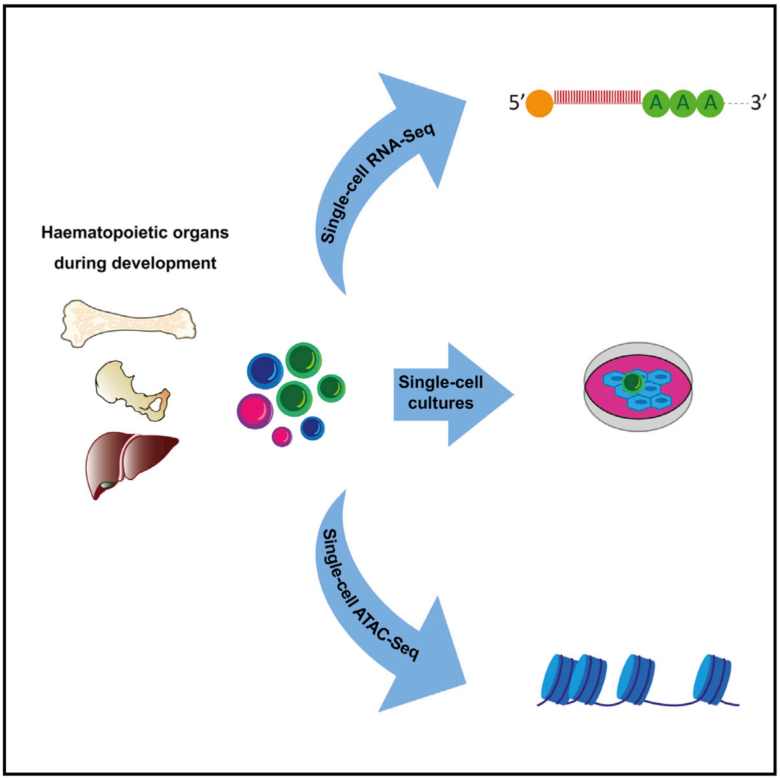

Highlights

- The epigenetic and transcriptional landscape of human fetal hematopoiesis
- Blood stem cells differentiate into three distinct oligopotent progenitor populations
- Changes in motif accessibility in blood stem cells precede transcriptional priming
- Refined sorting strategy to isolate and enrich for human fetal blood stem cells

Authors

Anna Maria Ranzoni,
Andrea Tangherloni, Ivan Berest, ...,
Irina Mohorianu, Judith B. Zaugg,
Ana Cvejic

Correspondence

as889@cam.ac.uk

In Brief

Ranzoni et al. provide a detailed transcriptional and chromatin accessibility map of fetal liver and bone marrow hematopoietic stem cells (HSCs). Within HSCs, they revealed extensive epigenetic but not transcriptional priming. They identified transcriptional and functional differences between HSCs from liver and bone marrow.

Ranzoni et al., 2021, Cell Stem Cell *28*, 472–487
March 4, 2021 © 2020 The Authors. Published by Elsevier Inc.
https://doi.org/10.1016/j.stem.2020.11.015

CellPress

CellPress
OPEN ACCESS

Cell Stem Cell

Article

Integrative Single-Cell RNA-Seq and ATAC-Seq Analysis of Human Developmental Hematopoiesis

Anna Maria Ranzoni,[1,2,3,6] Andrea Tangherloni,[1,2,3,6] Ivan Berest,[4,6] Simone Giovanni Riva,[1,2,3] Brynelle Myers,[2,3] Paulina M. Strzelecka,[1,3,5] Jiarui Xu,[1,2,3] Elisa Panada,[2,3] Irina Mohorianu,[2] Judith B. Zaugg,[4] and Ana Cvejic[1,2,3,7,*]
[1]University of Cambridge, Department of Haematology, Cambridge CB2 0AW, UK
[2]Wellcome Trust – Medical Research Council Cambridge Stem Cell Institute, Cambridge CB2 0AW, UK
[3]Wellcome Trust Sanger Institute, Wellcome Trust Genome Campus, Hinxton CB10 1SA, UK
[4]European Molecular Biology Laboratory, Structural and Computational Biology Unit, Meyerhofstrasse 1, 69115 Heidelberg, Germany
[5]Present address: Charité Universitätsmedizin Berlin, 13353 Berlin, Germany
[6]These authors contributed equally
[7]Lead Contact
*Correspondence: as889@cam.ac.uk
https://doi.org/10.1016/j.stem.2020.11.015

SUMMARY

Regulation of hematopoiesis during human development remains poorly defined. Here we applied single-cell RNA sequencing (scRNA-seq) and single-cell assay for transposase-accessible chromatin sequencing (scATAC-seq) to over 8,000 human immunophenotypic blood cells from fetal liver and bone marrow. We inferred their differentiation trajectory and identified three highly proliferative oligopotent progenitor populations downstream of hematopoietic stem cells (HSCs)/multipotent progenitors (MPPs). Along this trajectory, we observed opposing patterns of chromatin accessibility and differentiation that coincided with dynamic changes in the activity of distinct lineage-specific transcription factors. Integrative analysis of chromatin accessibility and gene expression revealed extensive epigenetic but not transcriptional priming of HSCs/MPPs prior to their lineage commitment. Finally, we refined and functionally validated the sorting strategy for the HSCs/MPPs and achieved around 90% enrichment. Our study provides a useful framework for future investigation of human developmental hematopoiesis in the context of blood pathologies and regenerative medicine.

INTRODUCTION

During embryonic development, hematopoietic stem cells (HSCs) need to rapidly differentiate into mature blood cells. Our current knowledge of fetal hematopoietic stem and progenitor cells (HSPCs) has mainly been advanced by murine and *in vitro* model systems. It has been demonstrated that fetal hematopoiesis consists of several separate waves of specification, migration, and differentiation of rare HSCs at distinct organs during development (Ivanovs et al., 2017). In humans, definitive hematopoiesis starts with the appearance of HSCs within hematopoietic clusters, in the dorsal aorta, 27 days post-conception. These definitive HSCs first colonize the fetal liver at 4 post-conceptional weeks (pcw), where they expand in numbers. At 10.5 pcw, the hematopoietic site shifts once more to the cavities of bones (i.e., bone marrow [BM]), where adult hematopoiesis is established permanently. The first HSCs that seed the bone marrow are thought to continue to rapidly increase in numbers before undergoing a dramatic change in their proliferative and differentiation properties to accommodate the need for high production of differentiated progeny (Mikkola and Orkin, 2006).

Historically, differentiation processes in the hematopoietic system have been depicted as a series of intermediate steps, defined by panels of cell surface markers (i.e., cluster of differentiation [CD]). In this model, often represented as a "hematopoietic tree," HSCs give rise to increasingly lineage-restricted cell types, eventually leading to mature blood cells (Akashi et al., 1999; Weissman, 2000). This paradigm has shifted in the last 5 years, with several studies reporting the transcriptomes of thousands of single hematopoietic cells, isolated by cell surface markers, in the mouse model and in adult humans (Paul et al., 2015; Velten et al., 2017). These reports showed that progenitor populations, thought previously to be homogeneous, are actually very heterogeneous on the transcriptional level.

The mechanisms underlying early fate decisions in HSCs are largely unknown. It has been postulated that the stochastic expression of lineage-specific transcription factors (TFs) above the noise threshold can "lock" a cell into a distinct cell fate (Graf and Enver, 2009). In line with this, co-expression of genes associated with antagonistic lineages, including key TFs, have been observed in multipotent hematopoietic cells, albeit at low levels (Hu et al., 1997; Miyamoto et al., 2002). This points toward the presence of sub-populations of cells within the multipotent

 © 2020 The Authors. Published by Elsevier Inc.
This is an open access article under the CC BY license (http://creativecommons.org/licenses/by/4.0/).

compartment that are permissive for opposing cell fates prior to their lineage commitment, a phenomenon referred to as priming (Nimmo et al., 2015). More recently, single-cell RNA sequencing (scRNA-seq) of human HSPCs introduced a different concept of priming. Studies of adult bone marrow and fetal liver hematopoiesis have identified sub-populations of HSCs and multipotent progenitors (MPPs) with coordinated expression of marker genes, specific for distinct unilineage differentiation programs, that gradually increase along all differentiation branches (Velten et al., 2017; Popescu et al., 2019). In addition, there are some indications that lineage priming in the HSC compartment might be happening not only on the transcriptional but also at the epigenetic level (Nimmo et al., 2015). Data from single-cell assay for transposase-accessible chromatin sequencing (scATAC-seq) of phenotypic HSPCs from adult human bone marrow show that phenotypic MPPs have variations in chromatin accessibility consistent with a bias toward erythroid and lymphoid lineages (Buenrostro et al., 2018).

Here we performed an integrative analysis of scRNA-seq and scATAC-seq of more than 8,000 immunophenotypic HSPCs from 17–22 pcw human fetal liver, femur, and hip to define transcriptional and epigenetic changes during blood differentiation. We explored lineage priming at the transcriptional and chromatin levels in HSCs/MPPs and refined the sorting strategy for isolation of a highly enriched HSC/MPP population.

RESULTS

Single-Cell Transcriptome of the Hematopoietic Compartment in Human Fetal Liver and Bone Marrow

To capture the full repertoire of hematopoietic cells during fetal development, we single-cell-sorted phenotypically defined blood populations from matched (i.e., from the same individual) fetal livers, femora, and hip (iliac) bones between 17 and 22 pcw (Figure 1A). Cells from the liver, hip, and femur were sorted and processed independently in all experiments. Thus, each cell can be traced back to the fetus and organ it came from. We used a hierarchical approach where we first isolated non-committed (Lin− [CD3, CD8, CD11b, CD14, CD19, and CD56] CD34+ CD38−) progenitors that contain all immature hematopoietic populations and are present at a frequency of less than 0.1% of the total fetal bone marrow (Golfier et al., 2000), followed by a more restrictive panel to capture differentiated and mature cell types. We next isolated committed (Lin−, CD34+ CD38+) progenitors as well as phenotypic HSCs, MPPs, common myeloid progenitors (CMPs), megakaryocyte-erythroid progenitors (MEPs), granulocyte-monocyte progenitors (GMPs), and common lymphoid progenitors (CLPs). In addition, based on broad phenotypic markers, we sorted T cells, natural killer (NK) cells, innate lymphoid cells (ILCs), monocytes, dendritic cells, mast cells, basophils, neutrophils, eosinophils, erythroid progenitors, erythrocytes, immature megakaryocytes (MKs), mature MKs, progenitor B cells (pro-B cells), precursor B cells (pre-B cells), mature B cells, and endothelial cells (Table S1; Figure S1).

Single cells from 15 fetuses were processed for scRNA-seq using the SmartSeq2 protocol (Picelli et al., 2014) (Figure 1A). Overall, 4,504 cells passed quality control (QC) (Table S2) with an average of ~3,600 genes per cell and ~670,000 reads per cell (Figures S2A–S2C, S2K, and S2L). To exclude technical batch effects, we merged the datasets from all samples and tissues using autoencoders (AEs) and applied the batch-balanced *k* nearest neighbors (BBKNN) approach (Polański et al., 2020; Luecken et al., 2020) to the latent space (Tangherloni et al., 2019; Figure S2O). We applied the graph-based Leiden clustering algorithm (Traag et al., 2019) to the batch-corrected neighborhood graph. Based on differential expression (DE) analysis and the top 20 marker genes (Figure 1B) ranked by significance of standardized expression, we manually annotated 23 distinct populations. Within the hematopoietic progenitor compartment, we annotated clusters as HSCs/MPPs, cycling HSCs/MPPs (HSCs/MPPs-Cycle), lymphoid-myeloid progenitors (LMPs), MK-erythroid-mast progenitors (MEMPs), cycling MEMPs (MEMPs-Cycle), granulocytic progenitors (GPs), as well as numerous mature blood cell types, as shown in the uniform manifold approximation and projection (UMAP) space (Becht et al., 2018; Figure 1D).

Of the mature blood cell types, we identified clear transcriptional signatures of erythroid cells (expressing *HBG1*, *HBA1*, *GYPA*, and *ALAS2*), MKs (expressing *FLI1*, *ITGA2B*, and *GP9*), monocyte progenitors and monocytes (expressing *CD14*, *MPEG1*, and *CD33*), CD4+ monocytes, mast cells (expressing *CD63*, *GATA2*, and *HDC*), plasmacytoid dendritic cells (pDCs; expressing *IL3RA*, *IRF8*, *MPEG1*, and *JCHAIN*) with an additional cluster of highly cycling pDCs (expressing pDC and proliferation markers; e.g., *MKI67*), and granulocytes 1, 2, and 3 (expressing *AZU1*, *MPO*, and *PRTN3*) (Figure 1B; Figure S3). Although granulocytes were present in our dataset, we could not clearly distinguish neutrophils, basophils, and eosinophils because of the mixed expression signatures. In the lymphoid compartment, we identified NK cells (expressing *CD3D*, *IL2RB*, and *CD96*) and B cells (expressing *CD19* and *CD79B*) (Figure 1B; Figure S3). The B cell lineage included pro-B cells, which showed expression of *IGLL1* and *RAG1*, and pre-B cells, expressing high levels of *CD79B*, *VPREB1*, and *CD24* (Figure 1B). Finally, we identified a cluster of mature B cells expressing high levels of *IGHM* and decreased levels of *IGLL1* compared with pro/pre-B cell clusters (Figure 1B). We did not detect any T cells or ILCs in the liver or femur despite sorting phenotypic T cells and ILCs using broad cell surface markers for these populations. Unlike B cells, which mature in the BM, T cells derive from lymphoid progenitors that migrate from the BM to the thymus, where they complete their maturation. The development of ILCs is less understood, but there have been suggestions that ILC precursors migrate early from BM into non-hematopoietic tissues; e.g., gut (Cichocki et al., 2019). Because we only sorted BM and not thymus or gut, we might have captured only progenitors but not T cells and ILCs. By using a deep neural network (DNN) (LeCun et al., 2015) and the top 30 marker genes for each cluster, we were able to correctly classify the cells to the prospective clusters with 90.46% accuracy, confirming that our manual annotation of clusters well separated the distinct cell types/states (STAR Methods; Figure S4A).

In the last decade, human HSCs and other progenitor populations have been isolated and used in functional assays based on specific sets of cell surface markers. It has been suggested that the fetal hematopoietic progenitor compartment differs substantially from its adult counterpart (Notta et al., 2016). Our approach allowed us to compare the extent to which the phenotypic

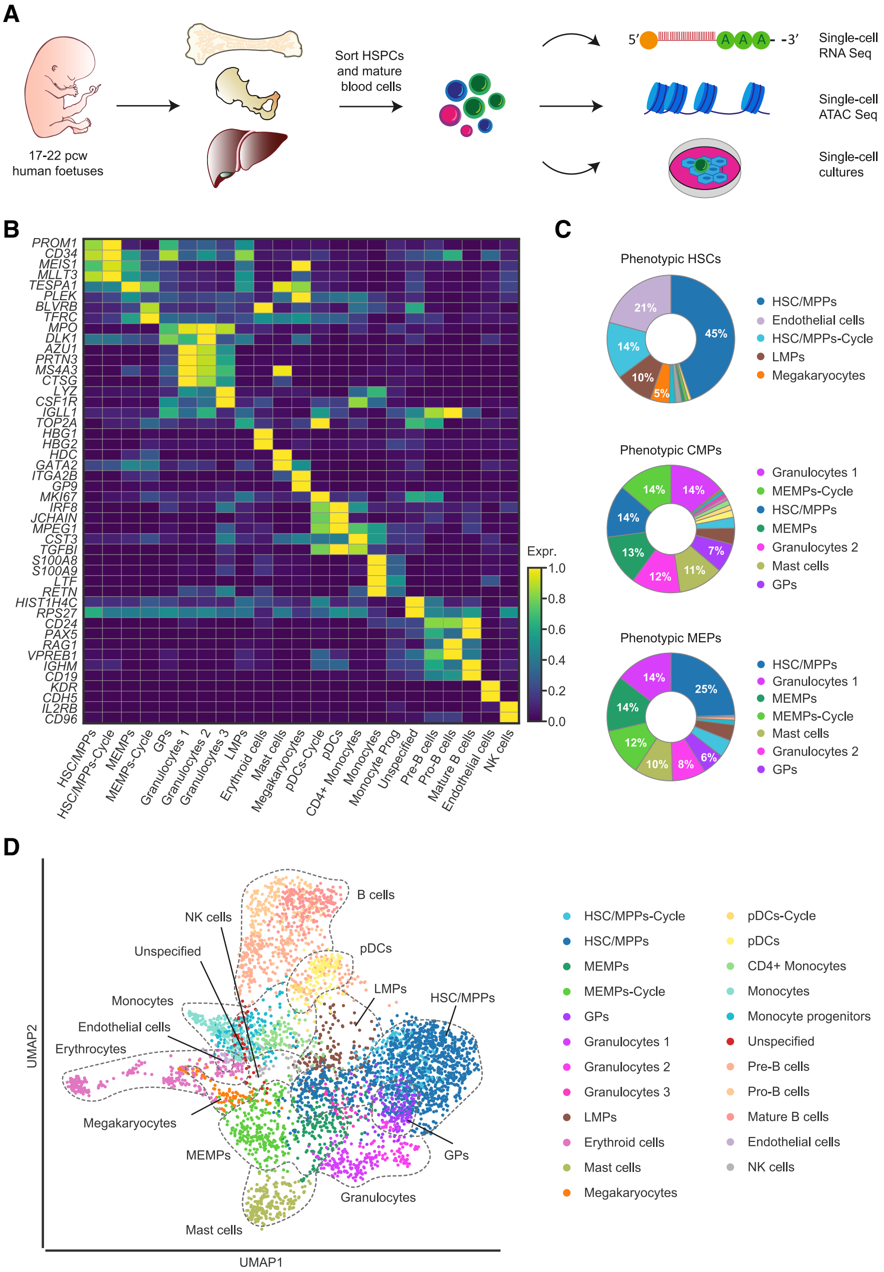

Figure 1. Single-Cell Transcriptome Analysis of Human Fetal Hematopoiesis

(A) Schematic overview of the experimental workflow. From each fetus (17–22 pcw), phenotypically defined HSPCs and mature blood cells were sorted from bone marrow (femur and hip) and liver and processed for scRNA-seq (n = 15), scATAC-seq (n = 3), and single-cell *in vitro* differentiation assays (n = 4).

(B) Heatmap of the mean expression value of two manually selected marker genes for each cell type. The expression of the genes is standardized between 0 and 1. For each gene, the minimum value is subtracted, and the result is divided by the maximum. The standardized expression level is indicated by color intensity.

(C) Donut plots showing the percentage of transcriptionally defined (i.e., manually curated) cell populations in each of the phenotypically defined stem and progenitor populations. The colors correspond to the identified cell types.

(D) UMAP visualization of hematopoietic cells from liver and bone marrow, colored by cell type. HSCs/MPPs-Cycle, cycling hematopoietic stem cells/multipotent progenitors; MEMPs, MK-Ery-mast progenitors; MEMPs-Cycle, cycling MEMPs; GPs, granulocytic progenitors; LMPs, Ly-My progenitors; pDCs, plasmacytoid dendritic cells; pDCs-Cycle, cycling pDCs.

identity of cell populations (as defined by CD markers) matched their transcriptional state (i.e., our manually curated clusters) and, thus, to critically examine use of CD markers in the context of fetal bone marrow hematopoiesis.

Single-cell analysis revealed substantial transcriptional heterogeneity within all immunophenotypically defined stem and progenitor populations, with some phenotypic progenitor populations (such as HSCs, MPPs, CMPs, GMPs, MEPs, and CLPs)

being comprised of more than 10 different transcriptionally defined populations. (Figure 1C; Figure S4B). This observation is in agreement with recent research showing a high level of heterogeneity of the progenitor compartment of human cord blood (Knapp et al., 2018). Our comparative analysis shows that currently used cell-surface markers are a poor predictor of the transcriptional state of human fetal hematopoietic progenitors.

Inference of Differentiation Trajectories during Fetal Hematopoiesis

Next we used a force-directed graph drawing algorithm, ForceAtlas2, to infer the differentiation trajectory of hematopoietic cells during human fetal development (Jacomy et al., 2014). We initialized a ForceAtlas2 layout with partition-based approximate graph abstraction (PAGA) coordinates from our annotated cell types (Wolf et al., 2019). This initialization generated interpretable single-cell embedding that is faithful to the global topology. The obtained global topology revealed HSCs/MPPs at the tip of the trajectory (Figures 2A and 2B; Figure S3). HSCs/MPPs showed high expression of *MLLT3*, a crucial regulator of human HSC maintenance (Calvanese et al., 2019); *HLF*, a TF involved in preserving quiescence in HSCs (Komorowska et al., 2017); and *MEIS1*, a TF involved in limiting oxidative stress in HSCs, which is necessary for quiescence (Unnisa et al., 2012; Wang et al., 2018). Cells in this cluster also expressed high levels of surface markers of HSPCs, such as *CD34* (Morisot et al., 2006), *SELL* (Ivanovs et al., 2017), and *PROM1* (de Wynter et al., 1998; Saha et al., 2020; Figures 1B and 2C). Downstream of HSCs/MPPs, we identified three distinct, highly proliferative, oligopotent progenitor populations. We used Scanpy's dpt function to infer progression of the cells through geodesic distance along the graph. Then we used Scanpy's paga_path function to show how the gene expression and annotation changes along the three main paths (MEMPs, GPs, and LMPs) present in the abstracted graph (Figure 2C).

MEMPs connected HSCs/MPPs with MKs, erythroid, and mast cells. In line with this, differentially regulated genes in the HSC/MPP transition to MEMPs included MK/erythroid/mast cell lineage-specific genes such as *GATA1*, *ITGA2B, PLEK*, *KLF1*, *HDC,* and *MS4A3* (Figures 1B and 2C; Figure S5B). The presence of MEMPs in our dataset is consistent with studies in mouse models proposing a common trajectory between erythroid, megakaryocytic, and mast cell lineages (Franco et al., 2010). This concept was more recently supported by a study in human fetal liver showing a shared progenitor of MKs, erythroid cells, and mast cells (Popescu et al., 2019). In addition, we identified a proliferative population of MEMPs-Cycle of which ~92% were in G2M/S phase compared with 65% of MEMPs (Figure 2E). The MEMPs-Cycle population further upregulated erythroid-specific genes such as *KLF1*, *BLVRB*, and *TFRC* compared with MEMPs, suggesting their gradual commitment toward the erythroid lineage (Figure S5C).

GPs connected the HSC/MPP cluster with granulocyte clusters. Cells in this cluster differentially expressed myeloid lineage-specific genes (e.g., *AZU1*, *LYZ*, and *MPO*) compared with HSCs/MMPs (Figure 2C; Figure S5D) and were highly cycling, with 73% of cells in G2M/S phase (Figure 2E). Finally, our data pointed toward the existence of a common progenitor population for B cells, monocytes, pDCs, and NK cells, here annotated as LMPs. Cells in this cluster expressed genes specific to those lineages, including *IGLL1*, *HMGB2*, and *CD79B* (lymphoid) (Figure 2C; Figure S3), and upregulated lymphoid genes such as *CD81*, *IGLL1*, and *HMGN2* compared with the HSC/MPP cluster (Figure S5E). Again, this was a highly proliferative population of cells with ~89% of cells in G2M/S phase (Figure 2E).

Our findings support previous studies of early lymphoid commitment in human cord blood *in vitro* and *in vivo* that identified a shared lineage progenitor between lymphoid, NK, B, and T cells; monocytes; and dendritic cells (Doulatov et al., 2010; Collin et al., 2011). Interestingly, the LMP cluster had higher expression of MPP-related genes such as *SPINK2*, *CD52*, and *SELL* compared with MEMPs, suggesting that these progenitors represent a more immature population compared with MEMPs (Figure S5F).

Next we used the Python implementation of single-cell regulatory network inference and clustering (SCENIC) (Aibar et al., 2017; Van de Sande et al., 2020) to identify master regulators and gene-regulatory networks (GRNs) in HSPCs and mature blood cells across differentiation trajectories. We found 162 regulons, some of which some were enriched across many different cell types, often as a part of the particular differentiation branch, and some were cell type specific (Figure 2D). We identified HLF and HOXA9 as main regulons in HSCs/MPPs, whereas GATA1, GATA2, and TAL1 were identified in the MEMP branch of the hematopoietic tree (Figure 2D). FOXO3 was highly specific for erythroid cells and EOMES, OLIG2, and IRF8 for NK cells, monocytes, and pDCs, respectively. Importantly, the regulons confirmed the inferred differentiation trajectory.

To further explore heterogeneity within the HSC/MPP population, we examined whether HSCs/MPPs simultaneously primed several different lineage-affiliated programs of gene activity. Although HSCs/MPPs sporadically expressed lymphoid, myeloid, or MK-erythroid differentiation genes, we did not observe consistent expression of antagonistic lineage-affiliated genes in individual cells. In addition, after further sub-clustering the HSCs/MPPs, there was no evident consolidation of lineage-affiliated transcriptional programs in any of the sub-populations (Figure S6). Our scRNA-seq data thus do not support recently reported transcriptional lineage priming in the fetal HSC/MPP compartment (Popescu et al., 2019) and suggest that, transcriptionally, our HSC/MPP cluster represents a highly immature population of cells.

DE analysis between HSCs/MPPs-Cycle and HSCs/MPPs revealed upregulation of genes involved in cell cycle regulation (*FOS*, *PTP4A1*, *MCL1*, and *PKN2*) in HSCs/MPPs-Cycle (Figure S5A), confirming that they are indeed a population of cycling stem and multipotent cells. In line with this, cell cycle analysis confirmed that ~36% of HSCs/MPPs were cycling compared with ~53% of HSCs/MPPs-Cycle (Figure 2E). HSCs/MPPs-Cycle had increased expression of genes involved in glycolysis, a feature commonly found in proliferating cells (Ito and Suda, 2014; Figure S5G). However, there were no other transcriptional differences between HSCs/MPPs and HSCs/MPPs-Cycle, excluding the presence of transcriptional priming in the HSCs/MPPs-Cycle cluster.

Previous research showed that, contrary to adult blood progenitors that are mainly unilineage, fetal liver blood progenitors maintain

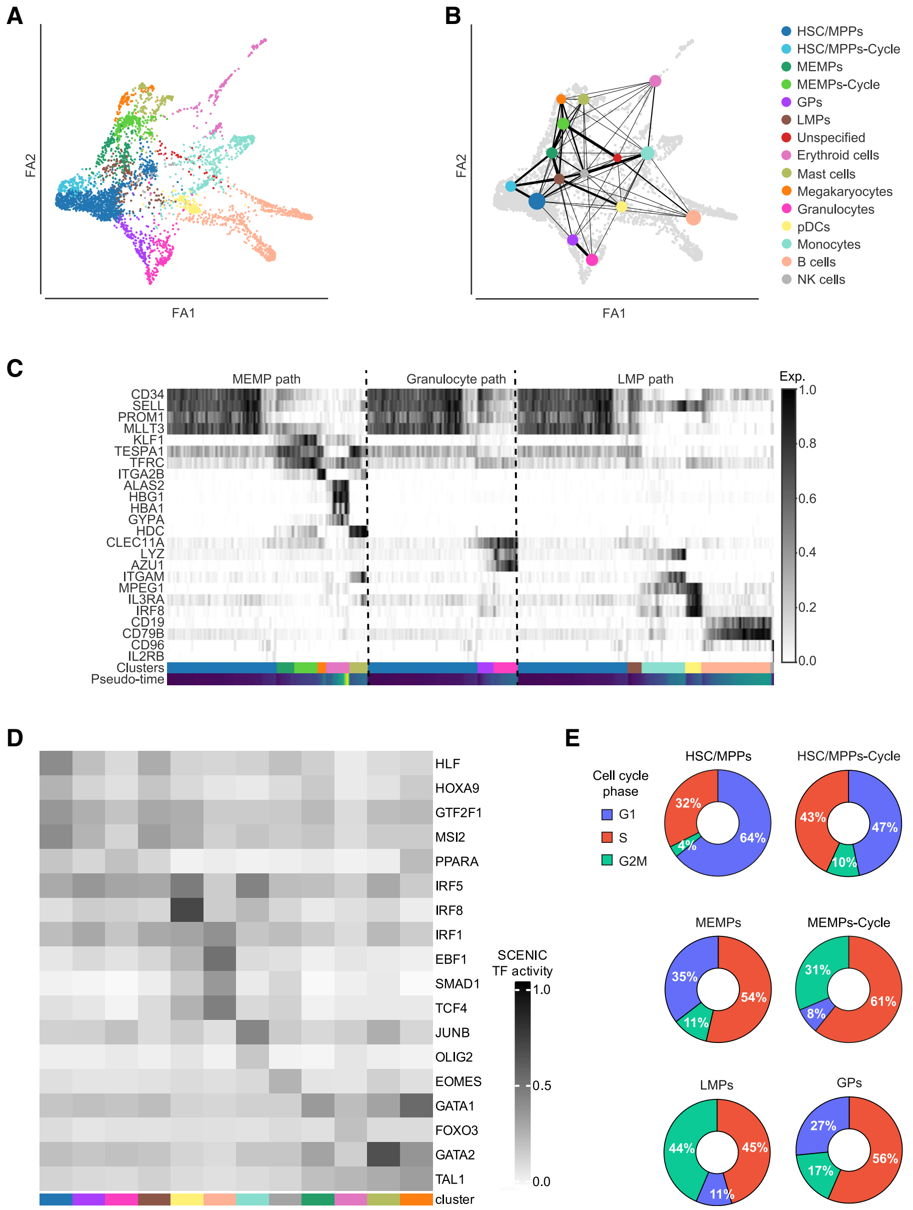

Figure 2. Differentiation Trajectory of Human Fetal Hematopoietic Cells

(A) Force-directed graph (FDG) visualization of the differentiation trajectory of hematopoietic cells from Figure 1D.

(B) PAGA trajectory model imposed on the FDG visualization of the differentiation trajectory. The size of the dots is proportional to the number of cells in the clusters.

(C) Heatmap showing dynamic expression of lineage-specific genes along the three differentiation paths (MEMP, granulocyte, and LMP path). Cluster colors match those of (A) and (B). Expression is standardized between 0 and 1, and the level is indicated by the grayscale color intensity.

(D) Heatmap of the normalized area under the curve (AUC) score of selected TFs for each cell type obtained by pySCENIC. Cluster colors match those of (A) and (B). The AUC score is standardized between 0 and 1 and indicated by the grayscale color intensity.

(E) Donut plots showing the percentages of cells in G1, S, and G2M phase in HSCs/MPPs, HSCs/MPPs-Cycle, MEMPs, MEMPs-Cycle, LMPs, and GPs. FA2, ForceAtlas2.

multilineage potential (Notta et al., 2016). Our data are consistent with this observation and point toward the existence of three oligopotent progenitor populations downstream of the HSC/MPP compartments: MEMPs giving rise to erythroid cells, MKs, and mast cells; GPs differentiating into granulocytes; and LMPs generating lymphoid cells, monocytes, and dendritic cells.

scATAC-Seq of Fetal Non-committed Progenitors (CD34+ CD38–)

Detection of low-abundance transcripts, such as TFs, might be difficult in scRNA-seq data because of technical limitations of the approach, leading to false negatives (so-called dropouts). The activity of these TFs can be inferred, however, from

CellPress
OPEN ACCESS

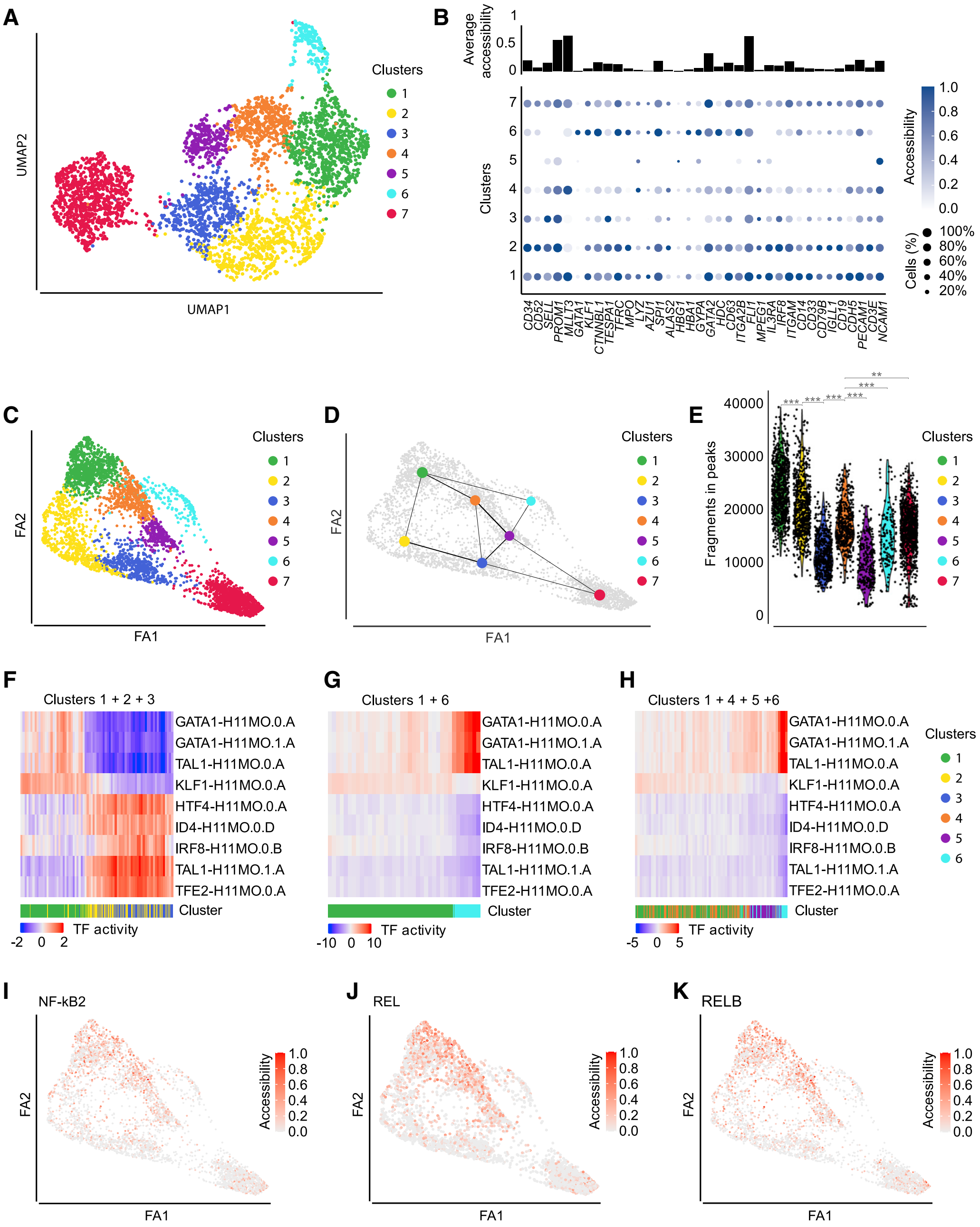

Figure 3. Single-Cell Chromatin Accessibility Analysis of Human Fetal Hematopoiesis

(A) UMAP visualization of the scATAC-seq dataset (3,611 nuclei from CD34+ CD38– cells from the liver and bone marrow), colored by cluster.

(B) Top: bar plot showing the average accessibility of 36 selected marker genes from our scRNA-seq data considering all cells. Bottom: dot plot of the standardized accessibility of the marker genes (gene body ± 3 kb) in each of the seven clusters. For each gene, the minimum value of its accessibility is subtracted, and the result is divided by the maximum value of its accessibility. The dot size indicates the percentage of cells in each cluster in which the gene of interest is accessible. The standardized accessibility level is indicated by color intensity.

(C) FDG visualization of the differentiation trajectory of hematopoietic cells from (A).

(D) PAGA trajectory model imposed on the FDG visualization of the differentiation trajectory of hematopoietic cells from (A). The size of the dots is proportional to the number of cells in the clusters.

(E) Violin plots showing the chromatin accessibility in different clusters. $p_{1,2} < 2 \times 10^{-16}$, $p_{2,3} < 2 \times 10^{-16}$, $p_{3,4} < 2 \times 10^{-16}$, $p_{4,5} < 2 \times 10^{-16}$, $p_{4,6} = 1.4E{-}07$, $p_{4,7} = 0.00235$. ***$p < 0.001$; $0.001 <$ **$p < 0.01$; $0.01 <$ *$p < 0.05$; ns, not significant; $p \geq 0.05$.

(F–H) Heatmap showing the activity of lineage-specific TFs along differentiation trajectories.

(legend continued on next page)

chromatin accessibility, emphasizing the importance of approaches integrating scRNA-seq and scATAC-seq data. In addition, chromatin accessibility at regulatory regions might precede gene activity and, thus, have predictive value for future transcription of a gene. Therefore, to further investigate the regulatory events in very immature cell populations, we examined the single-cell chromatin accessibility landscape (using scATAC-seq) of human fetal Lin− CD34+ CD38− cells (STAR Methods). We sequenced 4,001 cells from liver and femur of three fetuses, 18, 20, and 21 pcw (STAR Methods). Based on our scRNA-seq data, we expected that 90% of captured cells would be associated with one of the six populations: HSCs/MPPs, HSCs/MPPs-Cycle, MEMPs, MEMPs-Cycle, GPs, and LMPs, with HSCs/MPPs(Cycle) constituting the majority (Figure S4B).

To capture peaks that are present in less abundant cell types, such as MEMPs, MEMPs-Cycle, GPs, and LMPs, we employed an iterative peak-calling approach. We first defined open chromatin regions by pooling all data and calling peaks in the pooled samples. Following dimensionality reduction with diffusion maps (Haghverdi et al., 2015) and clustering using the Louvain community detection algorithm (Blondel et al., 2008), we performed a second round of peak calling in clusters with more than 50 cells. Of the initial ~474,000 reads, after preprocessing steps (Figures S2D–S2F), on average, we detected ~32,400 fragments per cell, and 56% of those mapped to peaks (Figures S2G, S2H, and S2M). Following filtering steps (Figures S2I, S2J, and S2N), 3,611 cells passed QC with 152,282 distinct peaks.

Motif Accessibility Dynamics along the Inferred Differentiation Trajectories

To merge samples and remove the batch effects, we applied Harmony (Korsunsky et al., 2019; Luecken et al., 2020) to the first 50 latent semantic indexing (LSI) components, excluding the first one because it was highly correlated with sequencing depth (Figures S2P and S2Q). By using a shared nearest neighbor (SNN) modularity optimization-based clustering algorithm, we obtained seven distinct clusters of differentially accessible peaks (Figure 3A).

To explore the chromatin accessibility profiles across the seven clusters, we examined the accessibility of selected marker genes from our scRNA-seq data (Figure 3B). We observed higher accessibility of marker genes associated with stem cells (e.g., *MLLT3*, *PROM1*, *FLI1*, and *GATA2*) and lower accessibility of genes associated with distinct lineages (e.g., *MPO*, *ALAS2*, *MPEG1*, and *CD19*), keeping in line with the undifferentiated nature of sorted cells (Figure 3B). Interestingly, we observed clear separation of clusters in terms of their overall accessibility of marker genes, with clusters 1, 2, 4, and 7 being more accessible and clusters 3 and 5 being less accessible. Cluster 6 had a mixed signature (Figure 3B).

Extensively open chromatin in multipotent cells has been associated previously with a permissive state to which multiple programs of gene regulation may be applied upon differentiation and is considered important for maintenance of pluripotency (Gaspar-Maia et al., 2011). To further investigate whether there were global dynamic changes in accessibility patterns associated with differentiation of fetal HSCs/MPPs, we inferred differentiation pseudotime from our scATAC-seq data using the same approach as with scRNA-seq described above. Briefly, we built a force-directed graph from our seven scATAC-seq clusters by initializing a ForceAtlas2 layout with PAGA coordinates (Figures 3C and 3D). The generated trajectory revealed two branches with a clear trend between chromatin accessibility and differentiation in each branch (Figures 3D and 3E). We observed the highest accessibility in clusters 1, 2, and 4, which decreased gradually toward the tips of the two branches (i.e., clusters 1, 2, and 3 on one side and 1, 4, 5, and 6 and 1 and 6 on the other; Figure 3E). This result is compatible with the notion that clusters 1, 2, and 4 represent an HSC/MPP population.

Control of gene expression is a dynamic process that involves cell-type-specific expression of TFs and establishment of an accessible chromatin state that permits binding of TFs to a defined motif. Thus, to assess regulatory programs that are active in HSPCs, we used chromVAR (Schep et al., 2017) to calculate the most variable accessible TF sequence motifs in different clusters and examine their activity along the differentiation trajectory. Along the two branches identified by the trajectory inference, we observed dynamic changes in the accessibility of lineage-specific hematopoietic TF motifs such as GATA1, TAL1, KLF1, HTF4, ID4, IRF8, and TFE2 (Figures 3F–3H).

GATA1 activity (Figures 3G and 3H) and gene body accessibility (Figure 3B) were enriched in cluster 6. GATA1 is known to be an important regulator of erythroid, megakaryocytic, and mast cells differentiation (Katsumura et al., 2017) and was expressed exclusively in the MEMP cluster in our scRNA-seq dataset. Thus, the identified trajectories between clusters 1 and 6 and clusters 1, 4, 5, and 6 most likely represent the MEMP differentiation paths (Figure 3D). Interestingly, in cluster 6, compared with clusters 2 and 3, we detected opposing patterns of motif accessibility for the two different TAL1 binding sites (TAL1.0.A and TAL1.1.A, respectively) (Figures 3F–3H). Substantial changes in occupancy by TAL1 during differentiation have been observed that are dependent on its binding partners (Wu et al., 2014). It has been reported previously that TAL1.0.A is co-occupied by TAL1 and GATA1 (Kassouf et al., 2010) and TAL1.1.A by TAL1 and TCF3 (Hsu et al., 1994). Our analysis revealed that the two different TAL1 binding motifs are active in distinct hematopoietic progenitor populations during fetal hematopoiesis (Figures 3F–3H).

Clusters 2 and 3 also showed increased activity of CEBPD and IRF8, crucial for myeloid and dendritic cell differentiation, and of

(F) Clusters 1, 2, and 3.
(G) Clusters 1–6.
(H) Clusters 1, 4, 5, and 6.
(I–K) FDG visualization of min-max normalized TF motif accessibility along the differentiation trajectory.
(I) NF-κB2.
(J) REL.
(K) RELB. The standardized accessibility level is indicated by color intensity.

ID4 and HTF4, involved in establishment of the lymphoid lineage (Miyazaki et al., 2017; Figures 3F–3H). This points toward clusters 1, 2, and 3 forming a common initial trajectory between the myeloid and lymphoid fate, consistent with our observations in scRNA-seq data. Clusters 1 and 4 were characterized by a high level of activity of TFs of the nuclear factor κB (NF-κB) pathway (i.e., NF-κB2, REL, and RELB) (Figures 3I–3K), known to be involved in regulation of HSC maintenance and self-renewal (Zhao et al., 2012; Espín-Palazón and Traver, 2016).

Integrating scRNA-Seq and scATAC-Seq Data

Next we wanted to map the cells from our scATAC-seq data to specific cell types. Because currently no chromatin accessibility maps are available for human fetal HSPCs, we chose a strategy to integrate our scRNA-seq and scATAC-seq by mapping cells based on their gene body accessibility. We used a recently developed method that identifies pairwise correspondences (called "anchors") between single cells across two different types of datasets and their transformation into the shared space (Stuart et al., 2019). This approach allowed us to transfer scRNA-seq-derived annotations, learned by a classifier, onto scATAC-seq data (STAR Methods).

We trained the classifier on CD34+ CD38− cells from the scRNA-seq experiment using the six most abundant cell types (STAR Methods). Overall, ~57% of scATAC-seq cells were assigned to the HSC/MPP cluster, ~18% to HSCs/MPPs-Cycle, ~5% to MEMPs, ~7% to MEMPs-Cycle, ~7% to GPs, and ~3% to LMPs. Cells with a prediction score lower than 40% were labeled as unclassified (~5%) (Figure 4A).

The frequencies of assigned cell types in the scATAC-seq dataset were highly concordant with the ones from scRNA-seq data (Figure S4B), suggesting that, overall, the two modalities (i.e., chromatin accessibility and transcriptome) are correlated. To validate the cell type assignment of scATAC-seq cells, we examined the accessibility of selected lineage-specific TF motifs in each of the annotated cell types (Figure 4B). In line with the predicted annotations, the GATA1 motif showed the highest accessibility in MEMPs and MEMPs-Cycle, whereas TEF2 (known to play a role in myeloid and lymphoid differentiation; Miyamoto et al., 2002) was most active in GPs and LMPs. Confirming our earlier observation, two distinct TAL1 motifs had anti-correlated accessibility. TAL1.0.A was preferentially active in MEMPs and MEMPs-Cycle and TAL1.1.A in GPs and LMPs (Figure 4B).

The Force Atlas representation of the classified scATAC-seq cells revealed, however, considerable intermixing of different cell types across the trajectory, with enrichment of MEMPs/MEMPs-Cycle in cluster 6 and, to a lesser extent, of GPs and LMPs in clusters 2 and 3 (Figure 4C). HSCs/MPPs(-Cycle) were distributed across all seven clusters. This wide distribution of HSCs/MPPs(-Cycle) across multiple clusters within scATAC-seq data suggested that, even though chromatin accessibility and the transcriptional state of fetal HSCs/MPPs are correlated, there is extensive chromatin priming in the HSC/MPP population that results in their heterogeneity.

Next, we compared the accessibility of selected lineage-specific TF motifs in HSCs/MPPs across the seven clusters (Figures 4D–4G). We observed a low level of activity of all examined TFs in cluster 1, followed by a statistically significant increase of HTF4, ID4, and TFE2 and decrease of GATA1 in HSCs/MPPs in clusters 2 and 3. GATA1 activity, however, increased in HSCs/MPPs in cluster 6. Our data suggest that, within the transcriptionally homogeneous population of HSCs/MPPs, there are significant differences in the activity of specific TFs that may precede gene expression and mark initial priming of HSCs/MPPs prior to their commitment to the specific lineage. To further explore this "time lag" between chromatin accessibility and gene expression during differentiation, we examined scRNA-seq and scATAC-seq data for the top GATA1-regulon target genes (ranked based on the AUCell score) identified by pySCENIC (Figure 5). We looked at the accessibility of both gene promoters (±3 kb from the transcriptional start site [TSS]) and distal regulatory regions (±50 kb from the TSS) as well as the expression levels of the selected target genes along the MEMP differentiation trajectory (Figure 5A). We observed that promoters of GATA1-regulon target genes were often open in HSCs/MPPs prior to any noticeable gene expression (Figure 5A). Thus, in line with our previous observation, chromatin accessibility in HSCs/MPPs preceded transcriptional changes that were only present in more differentiated cells. Interestingly, promoter accessibility of GATA1 target genes was overall lower in cluster 6 (MEMPs) compared with cluster 1 (HSCs/MPPs) (Figures 5B, 5D, and 5E) and coincided with lower promoter co-accessibility of the antagonistic genes (i.e., genes that are specific for distinct lineages) (Figure 5F). In contrast, the accessibility of distal regulatory elements/enhancers was higher in cluster 6 compared with cluster 1 (Figure 5C). This may indicate that GATA-regulon genes may be primed at promoters, whereas the enhancers contribute cell-type-specific expression.

Validation of HSC/MPP Identity and Their Differentiation Capacity

Given the observed limitation of commonly used sorting markers to isolate pure progenitor populations, we devised a new fluorescence-activated cell sorting (FACS) strategy for HSCs/MPPs based on cell surface markers selected from the top 20 marker genes for this cluster in our scRNA-seq dataset. The refined panel for HSCs/MPPs included Lin− CD34+ CD38− CD52+ CD62L+ CD133+ (hereafter called CD-REF; Figure 6A).

We sorted cells by FACS from femur BM using the CD-REF panel and profiled them again by scRNA-seq and single-cell *in vitro* differentiation assays. CD-REF cells, on average, accounted for 40% (±13%, n = 4) of Lin− CD34+ CD38− cells in the femur, based on FACS analysis. The scRNA-seq analysis of cells sorted with the refined panel showed that ~88% of CD-REF cells labeled HSCs/MPPs and HSCs/MPPs-Cycle clusters combined (Figure 6B) compared with commonly used CD panels for HSCs (Lin− CD34+ CD38− CD45RA− CD90+ CD49f+) and MPPs (Lin− CD34+ CD38− CD90− CD45RA− CD49f− CD10− CD7−), where ~59% and ~73%, respectively, of sorted cells had a transcriptional signature of our most immature cell population (Figure 1C; Figure S4B).

To assess the differentiation potential and robustness of the lineage output of CD-REF cells, we sorted individual cells from three fetuses on a mouse MS5 feeder layer or on more physiologically relevant, primary human fetal mesenchymal stem cells (fMSCs) (Figure 6C; STAR Methods). After 2 weeks, 80% of cells sorted on MS5 and 85% of cells sorted on human fMSCs

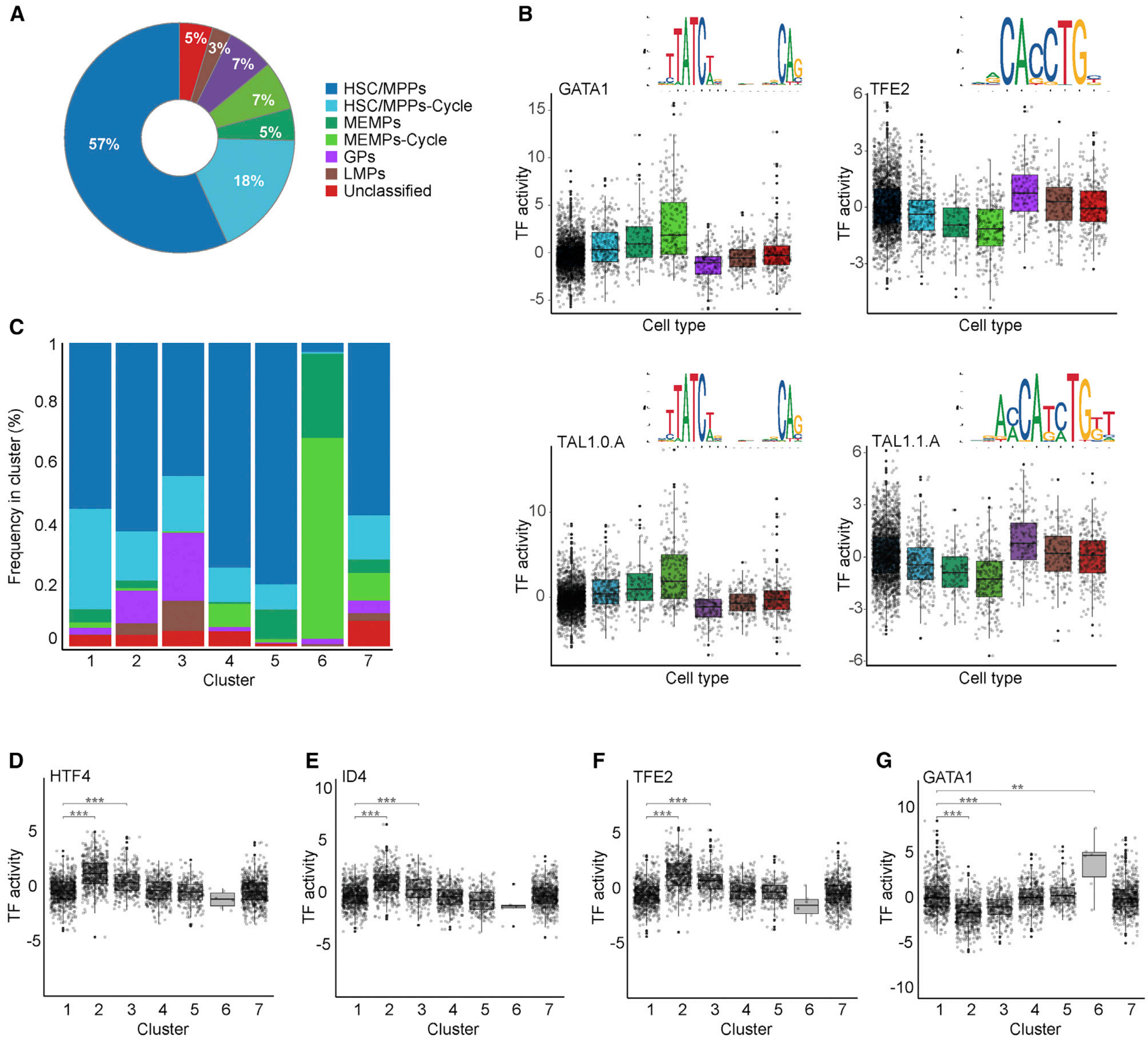

Figure 4. Integration of scRNA-Seq and scATAC-Seq Data

(A) Donut plot showing the percentage of scATAC-seq cells automatically assigned to different cell types.

(B) Boxplot showing the accessibility of GATA1, TFE2, TAL1.0.A, and TAL1.1.A motifs in the annotated cell types. On the top right of each boxplot, the TF sequence logos from the JASPAR database similar to the analyzed motifs are shown. Cluster colors match those of (A).

(C) Barplot showing the percentage of cells within each cluster assigned to the annotated cell types. Cluster colors match those in (A).

(D–G) Boxplots showing the accessibility of lineage-specific TF motifs in HSCs/MPPs across the seven clusters.

(D) HTF4 ($p_{1,2} < 2 \times 10^{-16}$, $p_{1,3} < 2 \times 10^{-16}$).

(E) ID4 ($p_{1,2} < 2 \times 10^{-16}$, $p_{1,3} = 10^{-13}$).

(F) TFE2 ($p_{1,2} < 2 \times 10^{-16}$, $p_{1,3} < 2 \times 10^{-16}$).

(G) GATA1 ($p_{1,2} < 2 \times 10^{-16}$, $p_{1,3} < 2 \times 10^{-16}$, $p_{1,6} = 0.0071$). ***$p < 0.001$; $0.001 <$ **$p < 0.01$; $0.01 <$ *$p < 0.05$; ns, $p \geq 0.05$.

generated colonies. In total, we analyzed 201 colonies for their size and lineage output (erythroid [Ery], myeloid [My], MK, and lymphoid [Ly]) using FACS (STAR Methods; Figure S7A). Our FACS analysis revealed that 7% of colonies on MS5 and 8% on fMSCs were quadri-lineage, 43% and 31% were tri-lineage, 29% and 25% were bilineage, 20% and 28% were unilineage, and 1% and 8% were undifferentiated colonies (Figure 6D).

Next we sorted individual CD-REFs and immunophenotypic HSCs (CD34+CD34−CD90+CD45RA−CD49f+/−) from the bone marrow and liver of the same fetus (n = 2) on an MS5 feeder layer and assessed 324 cells in total for their lineage output. Our analysis showed that liver- and femur-derived CD-REF cells had comparable efficacy of colony formation and lineage output, suggesting that CD-REF enriches for the population of cells

CellPress
OPEN ACCESS

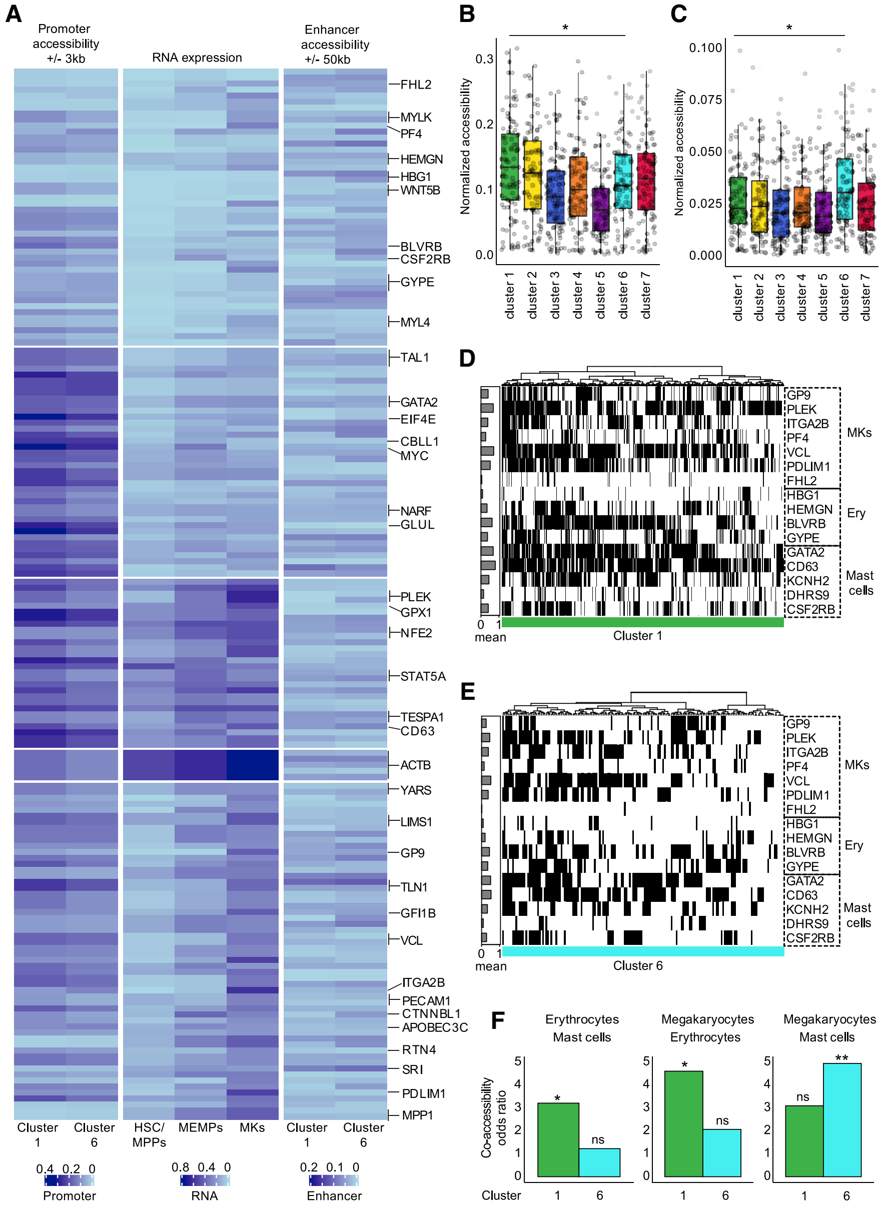

Figure 5. Chromatin Accessibility and Expression Dynamics of GATA1-Regulon Target Genes

(A) Heatmap showing the chromatin accessibility changes for promoters (left) and related distal regulatory elements (right) as well as RNA expression (center) for the target genes of GATA1-regulon obtained from pySCENIC. Only target genes with importance higher than 4 were considered. We normalized the expression of the set of GATA1 target genes into the range [0, 1]. For the promoters, the reads that overlapped the TSS regions ± 3 kb were extracted, normalized, and scaled into the range [0, 1] for each cell. To identify the enhancers for each gene, we took peaks around TSS ± 50 kb (excluding a ± 3 kb region), which have predicted GATA1 binding sites within them. We used normalized values for such peaks and scaled values from 0 to 1. The resulting values were summarized using mean per cluster. For visualization purposes, we pooled all data and clustered with 5 centroids using k-means. The standardized value levels are indicated by color intensity.

(B and C) Boxplot showing the difference in chromatin accessibility for GATA1-regulon genes for all identified scATAC-seq clusters. Values were obtained following similar criteria as described in (A).

(B) Promoters. Significant p values: $p_{1,3} = 7.7 \times 10^{-5}$, $p_{1,4} = 0.005$, $p_{1,5} = 3 \times 10^{-9}$, $p_{1,6} = 0.026$, $p_{2,3} = 0.001$, $p_{2,4} = 0.047$, $p_{2,5} = 1.8 \times 10^{-7}$, $p_{3,5} = 0.023$, $p_{3,6} = 0.034$, $p_{3,7} = 0.018$, $p_{4,5} = 3.4 \times 10^{-4}$, $p_{5,6} = 8.7 \times 10^{-6}$, $p_{5,7} = 5.5 \times 10^{-6}$.

(C) Enhancers. Significant p values: $p_{1,6} = 0.017$, $p_{3,6} = 0.006$, $p_{3,6} = 2.9 \times 10^{-4}$, $p_{4,6} = 9.1 \times 10^{-4}$, $p_{5,6} = 1.3 \times 10^{-4}$, $p_{6,7} = 0.004$.

(D and E) Heatmaps of the binarized chromatin accessibility in cluster 1 (D) and cluster 6 (E) for the promoters (±3 kb from TSS) of the selected marker genes of megakaryocytes (MKs), erythrocytes (Erys), and mast cells. Barplots on the left side of the heatmaps show the mean accessibility of the gene promoter for each cluster.

(F) Barplots showing the co-accessibility of promoters of lineage-specific marker genes. Fisher's exact test was used to check whether binarized accessibilities of promoters from different marker genes are associated with each other. Odds ratios of Fisher's exact tests are reported on the y axes. p (mast-Ery: cluster 1) = 0.03149, p (mast-Ery: cluster 6) = 0.6379; p (MK-Ery: cluster 1) = 0.01026; p (MK-Ery: cluster 6) = 0.1734; p (MK-mast: cluster 1) = 0.3007; p (MK-mast: cluster 6) = 0.005428.

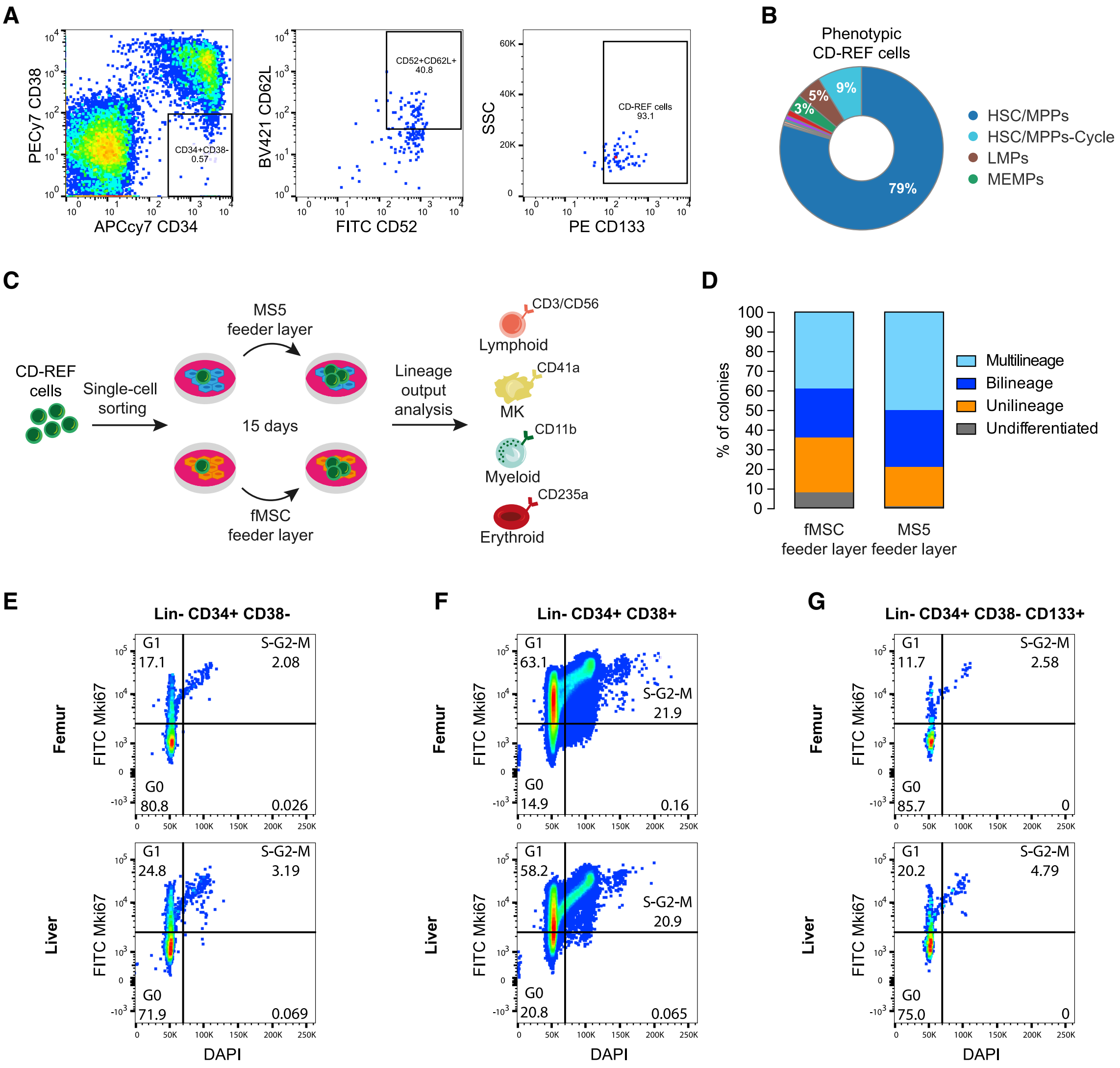

Figure 6. Refining the Sorting Strategy to Isolate Fetal HSCs/MPPs

(A) Novel FACS panel (CD-REF panel) designed to increase the purity of the sorted HSC/MPP population. After excluding debris, doublets, and Lin+ cells, CD34+ CD38− CD52+ CD62L+ CD133+ were sorted.

(B) Donut plots showing the percentage of transcriptionally defined (i.e., manually curated) cell populations in the phenotypically defined CD-REF population. The colors correspond to the identified cell types.

(C) Schematic overview of the single-cell *in vitro* differentiation assay. Single CD-REF cells were sorted in liquid culture with a mouse stromal cell line (MS5) or a human fetal primary feeder layer (fMSCs). After 15 days of culture, lineage output was assessed by expression of the lineage markers CD41a (MK), CD235a (Ery), CD3/CD56 (Ly), and CD11b (My) by flow cytometry.

(D) Percentage of colonies derived from single CD-REF cells characterized by quadrilineage, trilineage, bilineage, unilineage, and undifferentiated lineage output on two different feeder layers (n = 201 colonies, n = 2 fetuses per feeder layer).

(E–G) Representative flow cytometry images of cell cycle analysis by Mki67/DAPI co-staining of CD34+CD38− (E), CD34+CD38+ (F), and CD34+CD38−CD133+ (G) in matched fetal liver and femur.

with multilineage output in fetal liver and bone marrow. Similarly, the lineage output of CD-REF cells and immunophenotypic HSCs was comparable; however, the efficacy of colony formation appeared to be higher in CD-REF versus phenotypic HSCs isolated from the femur (Figure S7). Our finding that CD-REF cells indeed have multipotent potential and lineage output comparable with phenotypic HSCs is in line with our observation that these cells sit at the tip of the differentiation trajectories. We computationally and functionally confirmed that CD-REF represents a highly enriched population of HSC/MPPs.

Comparative Analysis of HSCs/MPPs from Different Hematopoietic Organs

Cells in the HSC/MPP cluster originated from the liver, femur, and hip. This provided a unique opportunity to assess potential qualitative and quantitative differences in the HSC/MPP

CellPress
OPEN ACCESS

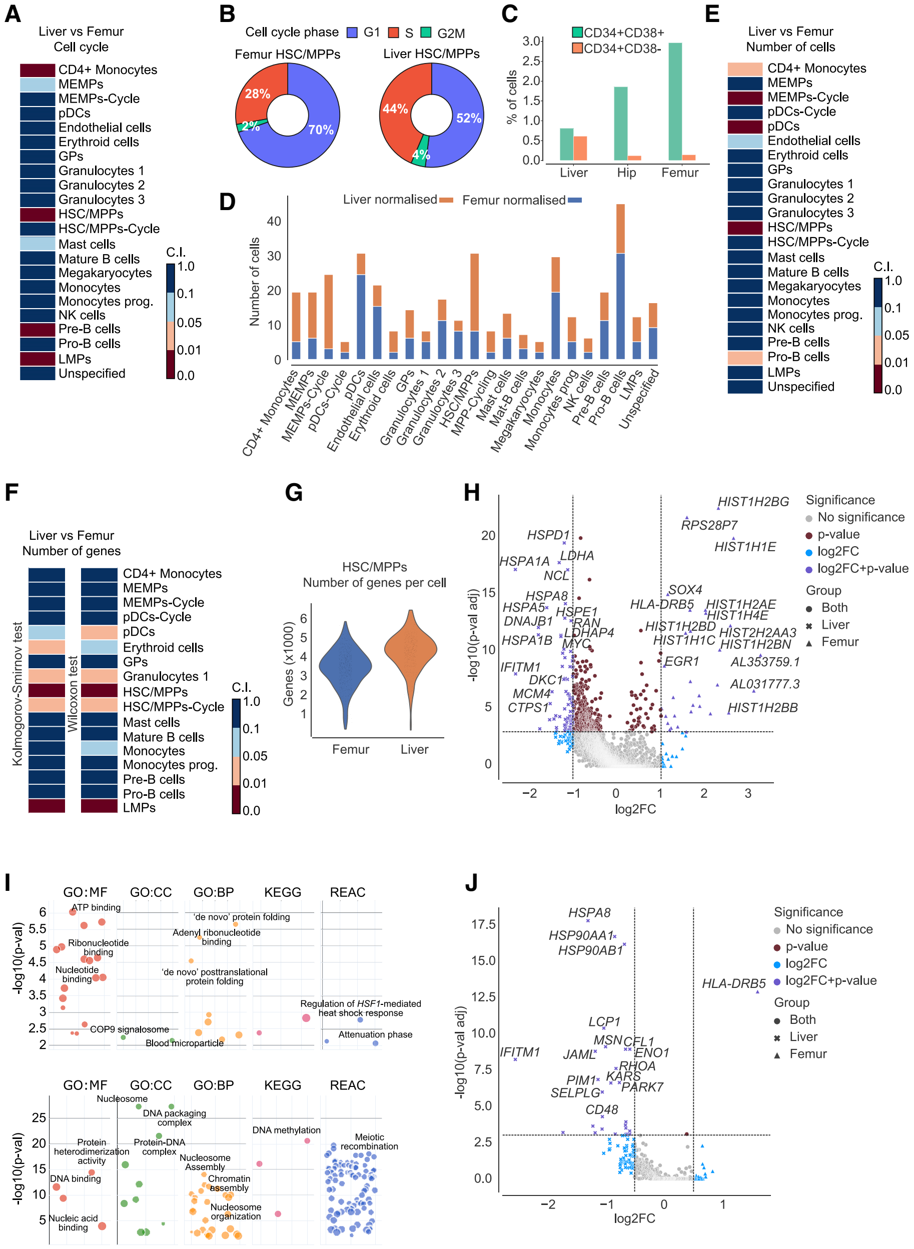

Figure 7. Statistically Significant Differences between Femur and Liver Cells across Cell Types

(A) Heatmap showing the confidence interval of Fisher's exact test on the normalized number of different hematopoietic cell types sorted from the liver and femur in G2M/S phase compared with G1 phase. The confidence interval is divided in four distinct levels, and each color identifies a statistical difference level.

(B) Donut plots displaying the percentage of cells in G1, G2M, and S phase in HSCs/MPPs sorted from femur or liver.

(C) Bar plot representing the proportion of CD34+ CD38− and CD34+ CD38+ cells of total live cells present in the liver and bone marrow (femur and hip); n = 15.

(D) Bar plot of the normalized distributions of the number of cells in each cell type sorted from liver or femur.

(E) Heatmap showing the confidence interval of Fisher's exact test on the normalized number of cells in each cell type collected from liver or femur. The confidence interval is divided in four distinct levels, and each color identifies a statistical difference level.

(F) Heatmaps depicting the confidence interval of the KS (left) and MWW (right) test on the number of expressed genes in each cell type collected from femur or liver cells. All confidence intervals are split into 4 subintervals (i.e., [0, 0.01], strong statistically significant difference; (0.01, 0.05], statistically significant difference; (0.05, 0.1], marginal statistically significant difference; (0.1, 1], no statistically significant difference).

(G) Violin plot of the number of expressed genes in HSCs/MPPs collected from the femur (blue) or liver (orange).

(H) Volcano plot showing differentially expressed genes (DEGs) in HSCs/MPPs collected from femur or liver cells. The x axis shows the $\log_2$ fold change (magnitude of change), whereas the y axis shows the −log10-adjusted p value (statistical significance). We used the Wilcoxon rank-sum test with Benjamini-Hochberg correction. Colors represent the significance of the genes in terms of p value and $\log_2$ fold change.

(legend continued on next page)

population that originated from fetal liver or bone marrow. We first applied Fisher's exact test to the number of liver and femur cells in the different cell cycle states to determine whether there are non-random associations between the cycle state and the organ of origin (see STAR Methods for further details). Interestingly, there was a statistically significant difference ($p = 4.25 \times 10^{-9}$) in the cell cycle state of cells in the HSC/MPP cluster between femur and liver (Figure 7A). Cells in the femur were predominantly in G1 (~70% of cells) compared with the same population in the liver (~52%) (Figure 7B). These data suggest that HSCs/MPPs become more quiescent as they migrate from the liver to the bone marrow during the second trimester of human development. In line with this, HSCs/MPPs were significantly less frequent in the femur compared with the liver (Figure 7D), as confirmed by Fisher's exact test on the total number of liver and femur cells (Figure 7E). This is in agreement with the increased proportion of phenotypic non-committed progenitors (CD34+ CD38−) found in the liver compared with the bone marrow (Figure 7C). Using Mki67 and DAPI staining, we quantified the proportion of cells in different stages of the cell cycle—G0 (Mki67−DAPI−), G1 (Mki67+DAPI−), and S-G2-M (Mki67+DAPI+)—as described previously (Kim and Sederstrom, 2015). Our analysis showed that the CD34+CD38− population is less cycling in the fetal liver and femur compared with the CD34+CD38+ population (Figures 6E and 6F). We further showed that the vast majority of CD-REF cells are in G0/G1 in the femur and liver but that nearly twice as many cells are in S-G2-M in the liver compared with the femur (Figure 6G).

To evaluate whether there is a statistically significant difference in the number of expressed genes between HSCs/MPPs collected from the liver and femur, we used the Kolmogorov-Smirnov (KS) and Mann-Whitney-Wilcoxon (MWW) test. We applied a subsampling strategy to downsample the cluster with more cells and balance the two distributions (STAR Methods). KS and MWW tests revealed a statistically significant decrease in the number of expressed genes in HSCs/MPPs in the femur compared with the liver (Figures 7F and 7G). Gene set enrichment analysis, using pathway databases, of differentially expressed genes between the liver and femur revealed that HSCs/MPPs in the femur upregulate genes involved in nucleosome assembly, chromatin assembly, and DNA packaging, such as *HIST1H1E* and *HIST1H2BN*, possibly marking their entry into quiescence (Figure 7H). Interestingly, DE analysis of genes that encode membrane proteins revealed statistically significant upregulation of genes related to actin cytoskeleton remodeling, cell adhesion, and migration (e.g., *JAML*, *SELPLG*, *LCP1*, *MSN*, and *RHOA*) in HSCs/MPPs in the liver compared with the femur (Figure 7I). This would be in line with the higher propensity of liver HSCs/MPPs to migrate to other tissues, such as bone marrow. In addition, we detected higher expression of interferon-induced gene *IFITM1* in fetal liver, known to play a role in transduction of antiproliferative and adhesion signals (Figure 7J). This shift of HSCs/MPPs from highly proliferative to quiescent as well as downregulation of genes involved in actin cytoskeleton remodeling as they migrate from fetal liver to bone marrow signifies the role of the niche in modulation of HSC/MPP behavior.

DISCUSSION

Here we present an integrative analysis of the single-cell transcriptome and chromatin accessibility of human fetal HSPCs. Our strategy involved plate-based sorting of well-defined immunophenotypic HSPCs from matched fetal liver and bone marrow. This approach enabled us to go beyond cataloging heterogeneity of cellular states during fetal hematopoiesis and to(1) examine the extent to which phenotypic markers used over the last decade coincided with the true nature of the sorted fetal blood populations, (2) refine the sorting strategy for HSCs/MPPs, (3) identify cell cycle and gene expression differences between HSCs/MPPs from fetal liver and bone marrow, (4) infer the HSPC differentiation trajectory, and (5) explore lineage priming within the HCS/MPP population.

In doing so, we observed a striking level of heterogeneity in all immunophenotypic HSPCs, with more than 10 transcriptionally defined cell populations identified in each of the progenitor populations. Although this is consistent with previous studies of human adult and cord blood hematopoiesis (Knapp et al., 2018), it further emphasized the need for refining the sorting strategy for human fetal HSPCs. Our CD-REF panel achieved nearly 90% enrichment of HSCs/MPPs, which we validated using single-cell *in vitro* differentiation assays and scRNA-seq. CD-REF cells comprised 40% of all CD34+ CD38− cells in the fetal bone marrow, with the majority of HSCs/MPPs not cycling. The shift from a highly proliferative state to quiescence coincided with the migration of HSCs/MPPs from the fetal liver to bone marrow, suggesting an important role of the niche in modulation of HSC/MPP behavior. This is remarkably different from previous studies in mice, where extensive proliferation of HSPCs in the bone marrow continued up to 3 weeks after birth (Bowie et al., 2006).

Downstream of HSCs/MPPs, we identified three highly proliferative oligopotent progenitor populations (MEMPs, LMPs, and GPs). Integrative scRNA-seq and scATAC-seq analysis of HSCs/MPPs and all main progenitor populations revealed a correlation between chromatin accessibility and gene expression but also pointed out that, within transcriptionally homogeneous HSCs/MPPs, there are multiple subpopulations that differ in their overall chromatin accessibility as well as lineage-specific TF activity. This indicates that, within the HSC/MPP population, regulatory programs permissive for different fates are being primed on the chromatin level, prior to their commitment to a specific lineage. The higher coordination of transcription and chromatin accessibility only occurred along commitment of HSCs/MPPs toward MEMPs, implying a hierarchy of different levels of

(I) Bubble plots showing the top Gene Ontology (GO) terms (MF, molecular function; CC, cellular component; BP, biological process), Kyoto Encyclopedia of Genes and Genomes, and Reactome, calculated by using the DEGs in HSCs/MPPs collected from liver (top) versus femur (bottom) cells.

(J) Volcano plot showing DEGs in HSCs/MPPs collected from femur or liver cells by considering only genes that encode plasma membrane proteins. The x axis shows the $\log_2$ fold change (magnitude of change), whereas the y axis shows the −log10-adjusted p value (statistical significance). We used the Wilcoxon rank-sum test with Benjamini-Hochberg correction. Colors represent the significance of the genes in terms of p value and $\log_2$ fold change.

commitment in the fetal progenitor compartment, with the MEMP population being the most committed (compared with LMPs and GPs).

Our study provides a high-resolution transcriptional and chromatin accessibility map of fetal HSPCs from the liver and bone marrow that will be essential for further exploration of HSCs/MPPs in the context of blood pathologies and for the purpose of regenerative medicine.

Limitations of Study

In this study, we characterized human fetal liver and bone marrow hematopoiesis using a combination of single-cell transcriptomics/epigenetics and *in vitro* single-cell differentiation assays. To avoid perturbations caused by freezing and thawing cycles, all experiments were performed on freshly isolated tissues. This experimental design and the nature of analyzed tissues come with a few limitations: (1) samples are rare, and (2) the cellularity varies significantly between different stages of development and individual fetuses, especially in the bone marrow. As a result, the number of cells available for analysis was limited. For this reason, we were not able to obtain enough cells to perform xenotransplantation experiments to confirm the stem cell identity and self-renewing potential of our CD-REF cells collected from bone marrow. Instead, we used single-cell *in vitro* assays as an alternative but not optimal readout of the multilineage potential of a cell. In addition, we could only collect a limited number of distinct phenotypically defined populations from individual fetuses; therefore, for any given population, the number of analyzed samples was relatively low.

STAR★METHODS

Detailed methods are provided in the online version of this paper and include the following:

- KEY RESOURCES TABLE
- RESOURCE AVAILABILITY
  - Lead Contact
  - Materials Availability
  - Data and Code Availability
- EXPERIMENTAL MODEL AND SUBJECT DETAILS
  - Ethics and Tissue acquisition
- METHOD DETAILS
  - Tissue processing
  - Fluorescence-activated cell sorting
  - Library preparation
  - Sequencing
  - Upstream analysis of scRNA-Seq data
  - Downstream analysis of scRNA-Seq data
  - Dimensionality reduction of scRNA-Seq data
  - Trajectory analysis of scRNA-Seq data
  - Differential expression analysis
  - Differentiation pathway analysis
  - Cell type classification
  - Upstream analysis of scATAC-Seq data
  - Downstream analysis of scATAC-Seq data
  - Dimensionality reduction of scATAC-Seq data
  - Trajectory analysis of scATAC-Seq data
  - Integration of scRNA-Seq and scATAC-Seq data
  - Transcription factor regulons prediction
  - Isolation of human fetal MSCs
  - Single-cell *in vitro* culture
  - Cell cycle analysis
- QUANTIFICATION AND STATISTICAL ANALYSIS
  - Differences across cell types

SUPPLEMENTAL INFORMATION

Supplemental Information can be found online at https://doi.org/10.1016/j.stem.2020.11.015.

ACKNOWLEDGMENTS

The authors would like to thank the WTSI Cytometry Core Facility for help with single-cell index sorting and the WTSI DNA pipelines and the CRUK Cambridge Institute Genomics Core Facility for contributions when sequencing the data. We would also like to thank the CellGen bioinformatics team for help with pre-processing of scRNA-seq data and Jana Eliasova for precious support and help with the illustrations. We would like to thank Alice Martin for help with pySCENIC analysis. Finally, we would like to thank the Human Developmental Biology Resource (HDBR) for providing samples. The study was supported by European Research Council Project 677501 – ZF_Blood (to A.C. and A.M.R.), an EMBO Young Investigator Award (to A.C.), and a core support grant from the Wellcome Trust and MRC to the Wellcome Trust – Medical Research Council Cambridge Stem Cell Institute (203151/Z/16/Z).

AUTHOR CONTRIBUTIONS

A.C. and A.M.R. conceived the study. A.M.R. performed all experiments with help from B.M., P.M.S., J.X., and E.P. A.T. carried out computational analysis of the scRNA-seq data. S.G.R. carried out parts of the computational analysis under supervision of A.T. I.B. performed analysis of the scATAC-seq data and integrative analysis of scATAC-seq and scRNA-seq under supervision of J.B.Z. I.M. carried out statistical analysis of *in vitro* experiments. A.C., A.M.R., and A.T. designed the figures and wrote the manuscript with input from the other authors. All authors approved the final version of the manuscript.

DECLARATION OF INTERESTS

The authors declare no competing interests.

Received: May 14, 2020
Revised: September 18, 2020
Accepted: November 19, 2020
Published: December 21, 2020

REFERENCES

Abadi, M., Agarwal, A., Barham, P., Brevdo, E., Chen, Z., Citro, C., Corrado, G.S., Davis, A., Dean, J., Devin, M., et al. (2016). TensorFlow: Large-Scale Machine Learning on Heterogeneous Distributed Systems. arXiv, arXiv:1603.04467 https://arxiv.org/abs/1603.04467.

Aibar, S., González-Blas, C.B., Moerman, T., Huynh-Thu, V.A., Imrichova, H., Hulselmans, G., Rambow, F., Marine, J.-C., Geurts, P., Aerts, J., et al. (2017). SCENIC: single-cell regulatory network inference and clustering. Nat. Methods *14*, 1083–1086.

Akashi, K., Kondo, M., Cheshier, S., Shizuru, J., Gandy, K., Domen, J., Mebius, R., Traver, D., and Weissman, I.L. (1999). Lymphoid development from stem cells and the common lymphocyte progenitors. Cold Spring Harb. Symp. Quant. Biol. *64*, 1–12.

Becht, E., McInnes, L., Healy, J., Dutertre, C.-A., Kwok, I.W.H., Ng, L.G., Ginhoux, F., and Newell, E.W. (2018). Dimensionality reduction for visualizing single-cell data using UMAP. Nat. Biotechnol. Published online December 3, 2018. https://doi.org/10.1038/nbt.4314.

Blondel, V.D., Guillaume, J.-L., Lambiotte, R., and Lefebvre, E. (2008). Fast unfolding of communities in large networks. J. Stat. Mech. *2008*, P10008.

Bowie, M.B., McKnight, K.D., Kent, D.G., McCaffrey, L., Hoodless, P.A., and Eaves, C.J. (2006). Hematopoietic stem cells proliferate until after birth and show a reversible phase-specific engraftment defect. J. Clin. Invest. *116*, 2808–2816.

Buenrostro, J.D., Corces, M.R., Lareau, C.A., Wu, B., Schep, A.N., Aryee, M.J., Majeti, R., Chang, H.Y., and Greenleaf, W.J. (2018). Integrated Single-Cell Analysis Maps the Continuous Regulatory Landscape of Human Hematopoietic Differentiation. Cell *173*, 1535–1548.e16.

Butler, A., Hoffman, P., Smibert, P., Papalexi, E., and Satija, R. (2018). Integrating single-cell transcriptomic data across different conditions, technologies, and species. Nat. Biotechnol. *36*, 411–420.

Calvanese, V., Nguyen, A.T., Bolan, T.J., Vavilina, A., Su, T., Lee, L.K., Wang, Y., Lay, F.D., Magnusson, M., Crooks, G.M., et al. (2019). MLLT3 governs human haematopoietic stem-cell self-renewal and engraftment. Nature *576*, 281–286.

Chen, X., Miragaia, R.J., Natarajan, K.N., and Teichmann, S.A. (2018). A rapid and robust method for single cell chromatin accessibility profiling. Nat. Commun. *9*, 5345.

Cichocki, F., Grzywacz, B., and Miller, J.S. (2019). Human NK Cell Development: One Road or Many? Front Immunol *10*, 2078. Published online August 29, 2019. https://doi.org/10.3389/fimmu.2019.02078.

Collin, M., Bigley, V., Haniffa, M., and Hambleton, S. (2011). Human dendritic cell deficiency: the missing ID? Nat. Rev. Immunol. *11*, 575–583.

de Wynter, E.A., Buck, D., Hart, C., Heywood, R., Coutinho, L.H., Clayton, A., Rafferty, J.A., Burt, D., Guenechea, G., Bueren, J.A., et al. (1998). CD34+AC133+ cells isolated from cord blood are highly enriched in long-term culture-initiating cells, NOD/SCID-repopulating cells and dendritic cell progenitors. Stem Cells *16*, 387–396.

Dobin, A., Davis, C.A., Schlesinger, F., Drenkow, J., Zaleski, C., Jha, S., Batut, P., Chaisson, M., and Gingeras, T.R. (2013). STAR: ultrafast universal RNA-seq aligner. Bioinformatics *29*, 15–21.

Doulatov, S., Notta, F., Eppert, K., Nguyen, L.T., Ohashi, P.S., and Dick, J.E. (2010). Revised map of the human progenitor hierarchy shows the origin of macrophages and dendritic cells in early lymphoid development. Nat. Immunol. *11*, 585–593.

Efremova, M., Vento-Tormo, M., Teichmann, S.A., and Vento-Tormo, R. (2020). CellPhoneDB: inferring cell-cell communication from combined expression of multi-subunit ligand-receptor complexes. Nat. Protoc. *15*, 1484–1506.

Espín-Palazón, R., and Traver, D. (2016). The NF-κB family: Key players during embryonic development and HSC emergence. Exp. Hematol. *44*, 519–527.

Fang, R., Preissl, S., Hou, X., Lucero, J., Wang, X., Motamedi, A., Shiau, A.K., Mukamel, E.A., Zhang, Y., Behrens, M.M., et al. (2019). SnapATAC: A Comprehensive Analysis Package for Single Cell ATAC-seq. bioRxiv. Published Online August 17, 2020. https://doi.org/10.1101/615179.

Franco, C.B., Chen, C.-C., Drukker, M., Weissman, I.L., and Galli, S.J. (2010). Distinguishing mast cell and granulocyte differentiation at the single-cell level. Cell Stem Cell *6*, 361–368.

Gaspar-Maia, A., Alajem, A., Meshorer, E., and Ramalho-Santos, M. (2011). Open chromatin in pluripotency and reprogramming. Nat. Rev. Mol. Cell Biol. *12*, 36–47.

Golfier, F., Bárcena, A., Harrison, M.R., and Muench, M.O. (2000). Fetal bone marrow as a source of stem cells for in utero or postnatal transplantation. Br. J. Haematol. *109*, 173–181.

Graf, T., and Enver, T. (2009). Forcing cells to change lineages. Nature *462*, 587–594, 10.1038/nature08533.

Haghverdi, L., Buettner, F., and Theis, F.J. (2015). Diffusion maps for high-dimensional single-cell analysis of differentiation data. Bioinformatics *31*, 2989–2998.

Hsu, H.L., Huang, L., Tsan, J.T., Funk, W., Wright, W.E., Hu, J.S., Kingston, R.E., and Baer, R. (1994). Preferred sequences for DNA recognition by the TAL1 helix-loop-helix proteins. Mol. Cell. Biol. *14*, 1256–1265.

Hu, M., Krause, D., Greaves, M., Sharkis, S., Dexter, M., Heyworth, C., and Enver, T. (1997). Multilineage gene expression precedes commitment in the hemopoietic system. Genes Dev. *11*, 774–785.

Ito, K., and Suda, T. (2014). Metabolic requirements for the maintenance of self-renewing stem cells. Nat. Rev. Mol. Cell Biol. *15*, 243–256.

Ivanovs, A., Rybtsov, S., Ng, E.S., Stanley, E.G., Elefanty, A.G., and Medvinsky, A. (2017). Human haematopoietic stem cell development: from the embryo to the dish. Development *144*, 2323–2337.

Jacomy, M., Venturini, T., Heymann, S., and Bastian, M. (2014). ForceAtlas2, a continuous graph layout algorithm for handy network visualization designed for the Gephi software. PLoS ONE *9*, e98679.

Kassouf, M.T., Hughes, J.R., Taylor, S., McGowan, S.J., Soneji, S., Green, A.L., Vyas, P., and Porcher, C. (2010). Genome-wide identification of TAL1's functional targets: insights into its mechanisms of action in primary erythroid cells. Genome Res. *20*, 1064–1083.

Katsumura, K.R., and Bresnick, E.H.; GATA Factor Mechanisms Group (2017). The GATA factor revolution in hematology. Blood *129*, 2092–2102.

Kim, K.H., and Sederstrom, J.M. (2015). Assaying cell cycle status using flow cytometry. Curr. Protoc. Mol. Biol. *111*, 28.6.1–28.6.11.

Kingma, D.P., and Ba, J. (2014). Adam: A Method for Stochastic Optimization. arXiv, arXiv:1412.6980 https://arxiv.org/abs/1412.6980.

Knapp, D.J.H.F., Hammond, C.A., Hui, T., van Loenhout, M.T.J., Wang, F., Aghaeepour, N., Miller, P.H., Moksa, M., Rabu, G.M., Beer, P.A., et al. (2018). Single-cell analysis identifies a CD33$^+$ subset of human cord blood cells with high regenerative potential. Nat. Cell Biol. *20*, 710–720.

Komorowska, K., Doyle, A., Wahlestedt, M., Subramaniam, A., Debnath, S., Chen, J., Soneji, S., Van Handel, B., Mikkola, H.K.A., Miharada, K., et al. (2017). Hepatic Leukemia Factor Maintains Quiescence of Hematopoietic Stem Cells and Protects the Stem Cell Pool during Regeneration. Cell Rep. *21*, 3514–3523.

Korsunsky, I., Millard, N., Fan, J., Slowikowski, K., Zhang, F., Wei, K., Baglaenko, Y., Brenner, M., Loh, P.-R., and Raychaudhuri, S. (2019). Fast, sensitive and accurate integration of single-cell data with Harmony. Nat. Methods *16*, 1289–1296.

Kulakovskiy, I.V., Vorontsov, I.E., Yevshin, I.S., Sharipov, R.N., Fedorova, A.D., Rumynskiy, E.I., Medvedeva, Y.A., Magana-Mora, A., Bajic, V.B., Papatsenko, D.A., et al. (2018). HOCOMOCO: towards a complete collection of transcription factor binding models for human and mouse via large-scale ChIP-Seq analysis. Nucleic Acids Res. *46* (D1), D252–D259.

LeCun, Y., Bengio, Y., and Hinton, G. (2015). Deep learning. Nature *521*, 436–444.

Li, H., and Durbin, R. (2009). Fast and accurate short read alignment with Burrows-Wheeler transform. Bioinformatics *25*, 1754–1760.

Li, H., Handsaker, B., Wysoker, A., Fennell, T., Ruan, J., Homer, N., Marth, G., Abecasis, G., and Durbin, R.; 1000 Genome Project Data Processing Subgroup (2009). The Sequence Alignment/Map format and SAMtools. Bioinformatics *25*, 2078–2079.

Luecken, M.D., and Theis, F.J. (2019). Current best practices in single-cell RNA-seq analysis: a tutorial. Mol. Syst. Biol. *15*, e8746.

Luecken, M.D., Büttner, M., Chaichoompu, K., Danese, A., Interlandi, M., Mueller, M.F., Strobl, D.C., Zappia, L., Dugas, M., Colomé-Tatché, M., et al. (2020). Benchmarking atlas-level data integration in single-cell genomics. bioRxiv. https://doi.org/10.1101/2020.05.22.111161.

Macaulay, I.C., Svensson, V., Labalette, C., Ferreira, L., Hamey, F., Voet, T., Teichmann, S.A., and Cvejic, A. (2016). Single-Cell RNA-Sequencing Reveals a Continuous Spectrum of Differentiation in Hematopoietic Cells. Cell Rep. *14*, 966–977.

Mikkola, H.K.A., and Orkin, S.H. (2006). The journey of developing hematopoietic stem cells. Development *133*, 3733–3744.

Miyamoto, T., Iwasaki, H., Reizis, B., Ye, M., Graf, T., Weissman, I.L., and Akashi, K. (2002). Myeloid or lymphoid promiscuity as a critical step in hematopoietic lineage commitment. Dev. Cell *3*, 137–147.

Miyazaki, M., Miyazaki, K., Chen, K., Jin, Y., Turner, J., Moore, A.J., Saito, R., Yoshida, K., Ogawa, S., Rodewald, H.-R., et al. (2017). The E-Id Protein Axis

Specifies Adaptive Lymphoid Cell Identity and Suppresses Thymic Innate Lymphoid Cell Development. Immunity *46*, 818–834.e4.

Morisot, S., Georgantas, R.W., and Civin, C.I. (2006). 345. Hematopoietic Stem-Progenitor Cells Express CD52 mRNA and Membrane Protein. Mol. Ther. *13*, S131–S132.

Nimmo, R.A., May, G.E., and Enver, T. (2015). Primed and ready: understanding lineage commitment through single cell analysis. Trends Cell Biol. *25*, 459–467.

Notta, F., Zandi, S., Takayama, N., Dobson, S., Gan, O.I., Wilson, G., Kaufmann, K.B., McLeod, J., Laurenti, E., Dunant, C.F., et al. (2016). Distinct routes of lineage development reshape the human blood hierarchy across ontogeny. Science *351*, aab2116.

Paul, F., Arkin, Y., Giladi, A., Jaitin, D.A., Kenigsberg, E., Keren-Shaul, H., Winter, D., Lara-Astiaso, D., Gury, M., Weiner, A., et al. (2015). Transcriptional heterogeneity and lineage commitment in myeloid progenitors. Cell *163*, 1663–1677.

Pedregosa, F., Varoquaux, G., Gramfort, A., Michel, V., Thirion, B., Grisel, O., Blondel, M., Prettenhofer, P., Weiss, R., Dubourg, V., et al. (2011). Scikit-learn: Machine Learning in Python. J. Mach. Learn. Res. *12*, 2825–2830.

Perpétuo, I.P., Bourne, L.E., and Orriss, I.R. (2019). Isolation and generation of osteoblasts. Methods Mol. Biol. *1914*, 21–38.

Picelli, S., Faridani, O.R., Björklund, A.K., Winberg, G., Sagasser, S., and Sandberg, R. (2014). Full-length RNA-seq from single cells using Smart-seq2. Nat. Protoc. *9*, 171–181.

Polański, K., Young, M.D., Miao, Z., Meyer, K.B., Teichmann, S.A., and Park, J.-E. (2020). BBKNN: fast batch alignment of single cell transcriptomes. Bioinformatics *36*, 964–965.

Popescu, D.M., Botting, R.A., Stephenson, E., Green, K., Webb, S., Jardine, L., Calderbank, E.F., Polanski, K., Goh, I., Efremova, M., et al. (2019). Decoding human fetal liver haematopoiesis. Nature *574*, 365–371.

Quinlan, A.R. (2014). BEDTools: The Swiss-Army Tool for Genome Feature Analysis. Curr. Protoc. Bioinformatics *47*, 11.12.1–11.12.34.

Raudvere, U., Kolberg, L., Kuzmin, I., Arak, T., Adler, P., Peterson, H., and Vilo, J. (2019). g:Profiler: a web server for functional enrichment analysis and conversions of gene lists (2019 update). Nucleic Acids Res. *47* (W1), W191–W198.

Saelens, W., Cannoodt, R., Todorov, H., and Saeys, Y. (2019). A comparison of single-cell trajectory inference methods. Nat. Biotechnol. *37*, 547–554.

Saha, S.K., Islam, S.M.R., Kwak, K.-S., Rahman, M.S., and Cho, S.-G. (2020). PROM1 and PROM2 expression differentially modulates clinical prognosis of cancer: a multiomics analysis. Cancer Gene Ther. *27*, 147–167.

Schep, A.N., Wu, B., Buenrostro, J.D., and Greenleaf, W.J. (2017). chromVAR: inferring transcription-factor-associated accessibility from single-cell epigenomic data. Nat. Methods *14*, 975–978.

Stuart, T., Butler, A., Hoffman, P., Hafemeister, C., Papalexi, E., Mauck, W.M., 3rd, Hao, Y., Stoeckius, M., Smibert, P., and Satija, R. (2019). Comprehensive Integration of Single-Cell Data. Cell *177*, 1888–1902.e21.

Tangherloni, A., Ricciuti, F., Besozzi, D., Lio, P., and Cvejic, A. (2019). scAEspy: a unifying tool based on autoencoders for the analysis of single-cell RNA sequencing data. bioRxiv. https://doi.org/10.1101/727867.

Traag, V.A., Waltman, L., and van Eck, N.J. (2019). From Louvain to Leiden: guaranteeing well-connected communities. Sci. Rep. *9*, 5233.

Unnisa, Z., Clark, J.P., Roychoudhury, J., Thomas, E., Tessarollo, L., Copeland, N.G., Jenkins, N.A., Grimes, H.L., and Kumar, A.R. (2012). Meis1 preserves hematopoietic stem cells in mice by limiting oxidative stress. Blood *120*, 4973–4981.

Van de Sande, B., Flerin, C., Davie, K., De Waegeneer, M., Hulselmans, G., Aibar, S., Seurinck, R., Saelens, W., Cannoodt, R., Rouchon, Q., et al. (2020). A scalable SCENIC workflow for single-cell gene regulatory network analysis. Nat. Protoc. *15*, 2247–2276.

Velten, L., Haas, S.F., Raffel, S., Blaszkiewicz, S., Islam, S., Hennig, B.P., Hirche, C., Lutz, C., Buss, E.C., Nowak, D., et al. (2017). Human haematopoietic stem cell lineage commitment is a continuous process. Nat. Cell Biol. *19*, 271–281.

Virtanen, P., Gommers, R., Oliphant, T.E., Haberland, M., Reddy, T., Cournapeau, D., Burovski, E., Peterson, P., Weckesser, W., Bright, J., et al.; SciPy 1.0 Contributors (2020). SciPy 1.0: fundamental algorithms for scientific computing in Python. Nat. Methods *17*, 261–272.

Wang, H., Liu, C., Liu, X., Wang, M., Wu, D., Gao, J., Su, P., Nakahata, T., Zhou, W., Xu, Y., et al. (2018). MEIS1 regulates hemogenic endothelial generation, megakaryopoiesis, and thrombopoiesis in human pluripotent stem cells by targeting TAL1 and FLI1. Stem Cell Reports *10*, 447–460.

Weissman, I.L. (2000). Stem cells: units of development, units of regeneration, and units in evolution. Cell *100*, 157–168.

Wolf, F.A., Angerer, P., and Theis, F.J. (2018). SCANPY: large-scale single-cell gene expression data analysis. Genome Biol. *19*, 15.

Wolf, F.A., Hamey, F.K., Plass, M., Solana, J., Dahlin, J.S., Göttgens, B., Rajewsky, N., Simon, L., and Theis, F.J. (2019). PAGA: graph abstraction reconciles clustering with trajectory inference through a topology preserving map of single cells. Genome Biol. *20*, 59.

Wu, W., Morrissey, C.S., Keller, C.A., Mishra, T., Pimkin, M., Blobel, G.A., Weiss, M.J., and Hardison, R.C. (2014). Dynamic shifts in occupancy by TAL1 are guided by GATA factors and drive large-scale reprogramming of gene expression during hematopoiesis. Genome Res. *24*, 1945–1962.

Zhang, Y., Liu, T., Meyer, C.A., Eeckhoute, J., Johnson, D.S., Bernstein, B.E., Nusbaum, C., Myers, R.M., Brown, M., Li, W., and Liu, X.S. (2008). Model-based analysis of ChIP-Seq (MACS). Genome Biol. *9*, R137.

Zhao, C., Xiu, Y., Ashton, J., Xing, L., Morita, Y., Jordan, C.T., and Boyce, B.F. (2012). Noncanonical NF-κB signaling regulates hematopoietic stem cell self-renewal and microenvironment interactions. Stem Cells *30*, 709–718.

STAR★METHODS

KEY RESOURCES TABLE

REAGENT or RESOURCE	SOURCE	IDENTIFIER
**Antibodies**		
CD3 AF700	Biolegend	OKT3; RRID:AB_493739
CD8 AF700	Biolegend	SK1; RRID:AB_2562789
CD11b AF700	Biolegend	CBRM1/5; RRID:AB_1963564
CD14 AF700	Thermo Fisher	61D3; RRID:AB_2574497
CD19 AF700	Biolegend	HIB19; RRID:AB_493750
CD56 AF700	BD Biosciences	B159; RRID:AB_396940
CD34 APC-Cy7	Biolegend	581; RRID:AB_1877168
CD38 PE-Cy7	BD Biosciences	HB7; RRID:AB_2868688
CD45RA BV785	Biolegend	HI100; RRID:AB_2563816
CD90 PE	BD Biosciences	5E10; RRID:AB_763533
CD49f PE-Cy5	BD Biosciences	GoH3; RRID:AB_394062
CD10 BUV737	BD Biosciences	HI10a; RRID:AB_2869630
CD7 FITC	BD Biosciences	MT701; RRID:AB_10895583
CD123 BV421	BD Biosciences	9F5; RRID:AB_2739392
CD45 FITC	Thermo Fisher	HI30; RRID:AB_10371768
CD52 FITC	Biolegend	HI186; RRID:AB_389276
CD62L BV421	Biolegend	DREG-56; RRID:AB_10896429
CD133 PE	Biolegend	Clone7; RRID:AB_2632879
CD127 PE	Biolegend	A019D5; RRID:AB_10719960
CD33 PE-Cy5	Biolegend	WM53; RRID:AB_314350
CD203c PE	Biolegend	NP4D6; RRID:AB_756043
CD63 APC	Biolegend	H5C6; RRID:AB_10916393
CD15 PE	Thermo Fisher	HI98; RRID:AB_11219674
CD16 APC	Biolegend	3G8; RRID:AB_314211
CD36 PE	Biolegend	5271; RRID:AB_1575032
CD71APC	Biolegend	CY1G4; RRID:AB_10916388
CD235a APC	BD Biosciences	GAR2; RRID:AB_10894583
CD41 FITC	BD Biosciences	HIP8; RRID:AB_10718234
CD42 PE	BD Biosciences	HIP1; RRID:AB_395865
CD61 APC	Dako	Y251; RRID:AB_578603
CD31 FITC	Biolegend	WM59; RRID:AB_314329
CD144 PE	Biolegend	BV9; RRID:AB_2260350
CD309 APC	Biolegend	7D46; RRID:AB_2565927
CD11b PE	Biolegend	ICRF44; RRID:AB_314157
**Biological Samples**		
Human fetal liver and bone marrow	Human Developmental Biology Resource (HDBR)	https://www.hdbr.org/
**Critical Commercial Assays**		
Nextera XT Library Prep Kit	Illumina	15032354
Nextera XT Index Kit v2	Illumina	15052164
KAPA Library Quant Kit (Illumina)	Roche	07960140001
**Deposited Data**		
Single-cell RNA Sequencing - raw data	This paper	ArrayExpress: E-MTAB-9067
Single-cell ATAC Sequencing - raw data	This paper	ArrayExpress: E-MTAB-9068

(*Continued on next page*)

Continued

REAGENT or RESOURCE	SOURCE	IDENTIFIER
Analyzed data	This paper	https://gitlab.com/cvejic-group/integrative-scrna-scatac-human-foetal#data
Human reference genome NCBI GRCh38	Genome Reference Consortium	https://www.ncbi.nlm.nih.gov/projects/genome/assembly/grc/human/
Experimental Models: Cell Lines		
MS5 cell line	DSMZ	ACC 441
Software and Algorithms		
Python	Python Programming Language	https://www.python.org/
R	The R Project for Statistical Computing	https://www.r-project.org/
STAR	Dobin et al., 2013	https://github.com/alexdobin/STAR
Bedtools	Quinlan, 2014	https://bedtools.readthedocs.io/en/latest/
Samtools	Li et al., 2009	http://www.htslib.org/
BWA	Li and Durbin, 2009	http://bio-bwa.sourceforge.net/
Picard	Broad Institute	http://broadinstitute.github.io/picard/
SnapATAC	Fang et al., 2019	https://github.com/r3fang/SnapATAC
Macs2	Zhang et al., 2008	https://github.com/macs3-project/MACS
Scanpy and dependencies	Wolf et al., 2018	https://scanpy.readthedocs.io/en/stable/
PAGA and dependencies	Wolf et al., 2019	https://github.com/theislab/paga
BBKNN	Polański et al., 2020	https://github.com/Teichlab/bbknn
Python version of g:Profiler	Raudvere et al., 2019	https://pypi.org/project/gprofiler-official/
scAEspy	Tangherloni et al., 2019	https://gitlab.com/cvejic-group/scaespy
Tensorflow	Abadi et al., 2016	https://www.tensorflow.org/
Keras	Chollet Lab	https://keras.io
Scikit-learn	Pedregosa et al., 2011	https://scikit-learn.org/stable/
Seurat and dependencies	Butler et al., 2018; Stuart et al., 2019	https://satijalab.org/seurat/
Signac	Satija Lab	https://satijalab.org/signac/
chromVAR	Schep et al., 2017	https://greenleaflab.github.io/chromVAR/
Harmony	Korsunsky et al., 2019	https://github.com/immunogenomics/harmony
Dynverse	Saelens et al., 2019	https://dynverse.org/
SCENIC and dependencies	Aibar et al., 2017	https://github.com/aertslab/SCENIC
pySCENIC and dependencies	Van de Sande et al., 2020	https://pyscenic.readthedocs.io/en/latest/
CellPhoneDB	Efremova et al., 2020	https://www.cellphonedb.org/
HOCOMOCO	Kulakovskiy et al., 2018	https://hocomoco11.autosome.ru/
Custom Python and R functions/scripts	This paper	https://gitlab.com/cvejic-group/integrative-scrna-scatac-human-foetal

RESOURCE AVAILABILITY

Lead Contact

Further information and requests for resources and reagents should be directed to and will be fulfilled by the Lead Contact, Dr Ana Cvejic (as889@cam.ac.uk).

Materials Availability

This study did not generate new unique reagents.

Data and Code Availability

The raw RNA-Seq data (i.e., fastq files) and cell assignment have been deposited to ArrayExpress: E-MTAB-9067, while the raw ATAC-Seq data (i.e., fastq files) and cell assignment have been deposited to ArrayExpress: E-MTAB-9068.

All scripts, functions, and Jupyter Notebook developed for this study are freely available on GitLab: https://gitlab.com/cvejic-group/integrative-scrna-scatac-human-foetal. The repository also contains the gene expression and fragment matrices.

EXPERIMENTAL MODEL AND SUBJECT DETAILS

Ethics and Tissue acquisition

Human fetal bone and liver samples were obtained from 33 fetuses aged 17-22 pcw, following termination of pregnancy and informed written consent. The human fetal material was provided by the Joint MRC/Wellcome Trust (Grant MR/R006237/1) Human Developmental Biology Resource (https://www.hdbr.org/), in accordance with ethical approval by the NHS Research Health Authority, REC Ref: 18/LO/0822.

METHOD DETAILS

Tissue processing

Tissues were kept in cold DMEM medium (Invitrogen) until dissection and processed on the same day of collection. Single-cell suspensions were generated from matched fetal liver and bone tissues after rinsing them with cold PBS (GIBCO). Liver samples were passed through a 70 μm strain into a falcon tube prefilled with cold PBS. Bone marrow from long bones was isolated by flushing cold PBS into the diaphysis and collected into a falcon tube. Bone marrow from hip bone was collected by dissecting the bone with a sterile scalpel and flushing cold PBS in the marrow cavity into a falcon tube. The suspension obtained from long bones and hip bones was then passed through a 70 μm strain into a new falcon tube. Cells were then centrifuged for 5 minutes at 300 g, 4°C and the pellet was resuspended into the RBC lysis buffer (eBioscience) for 2 minutes at room temperature, after which 20 mL of cold PBS were added to stop the lysis reaction. RBC step was not performed when sorting erythroid cells. Live cell enrichment was performed using MACS columns (Miltenyi Biotec - 130-090-101) following the manufacturer's instructions. When sorting CD34+ or CD45+ cells, column enrichment was performed using MACS columns (Miltenyi Biotec - 130-046-702 and 130-045-801 respectively for CD34+ and CD45+ cells), following the manufacturer's instructions.

Fluorescence-activated cell sorting

Cells were stained with antibody cocktails in a total volume of 100 μl 5% FBS (GIBCO) in PBS for 30 minutes at 4°C, centrifuged for 5 minutes at 300 g, 4°C, resuspended in a final volume of 500 μl of 5% FBS in PBS and subsequently filtered into polypropylene FACS tubes (ThermoFisher). For scRNA-Seq experiments, single cells were index sorted using a BD Influx Sorter into wells of 96-well plates (4titude) prefilled with 2 μl of lysis buffer consisting of 0.2% Triton X-100 (Sigma) and 1 U/μl RNase inhibitor (Life Technologies) in nuclease-free water (Invitrogen). For scATAC-Seq experiments, 5,000 - 20,000 cells were sorted using a BD Influx machine into 1.5 mL tubes (Eppendorf). Following bulk tagmentation with Tn5 (Chen et al., 2018), single nuclei were index sorted in wells of 384-well plates (Eppendorf) prefilled with 2 μl of lysis buffer consisting of 0.2% SDS, 20 μg/ml proteinase K (Ambion), 50 mM Tris-HCl (GIBCO) and 50mM NaCl (Sigma) in nuclease-free water.

Library preparation

The Smart-Seq2 method (Picelli et al., 2014) was used for library preparation for the scRNA-Seq experiments, with some modifications as described in Macaulay et al. (2016). The quality of libraries was evaluated with Bioanalyzer (Agilent). Good-quality libraries were subsequently quantified with KAPA Library Quantification Kit (Roche) and submitted for sequencing. Library preparation for the scATAC-Seq experiments was performed using a recently described method (Chen et al., 2018). Library traces were evaluated using Bioanalyzer.

Sequencing

Libraries for scRNA-Seq experiments were multiplexed using Nextera Index sets A, B, C, and D (v.2, Illumina) and sequenced on HiSeq4000 and NovaSeq6000 (Illumina) in pair-end mode, with an interquartile range (IQR) of 697,427 uniquely mapped reads (average: 666,632; standard deviation: 557,274). Libraries for scATAC-Seq experiments were sequenced on HiSeq4000 in pair-end mode, with a mean read count of 473,886 and IQR 341,210.

Upstream analysis of scRNA-Seq data

Smart-Seq2 sample demultiplex fastq files were quality checked, aligned and quantified by using the scRNA-Seq pipeline. This pipeline is based on STAR with default parameters (v.2.5.4a) (Dobin et al., 2013) index and annotation from the Ensembl release 91 of the GRCh38 human reference genome. Transcript and gene counts were quantified using the option *quantMode GeneCounts* provided by STAR. Since we used different sets of well-defined antibodies to isolate different cell types, we applied specific thresholds for each sample to filter out both the cells and genes (Table S3). We detected on average 3,642 genes per cell (IQR: 2,239; standard deviation: 1,621).

Downstream analysis of scRNA-Seq data

In what follows, for each function that we applied, we only report the parameter settings we modified. All other parameter settings of the functions are the default ones provided by the used computational libraries.We performed the downstream analysis of scRNA-Seq using the Python (v.3.6.9) package SCANPY (v.1.4.5.1) (Wolf et al., 2018). Our pipeline included: 1) a QC step (number of identified counts and number of expressed genes using the *filter_cells* function, and the fraction of mitochondrial genes). We obtained the 4,504 cells that were used in the next steps (Table S2), 2) removing the genes expressed in less than 10 cells (*filter_genes* function), 3) data normalization (*normalize_per_cell* function with scaling factor 10,000 and *log1p* function), 4) detection of the top 1,000 highly variable genes (HVGs) (*highly_variable_genes* function, in which the HVGs were selected separately within each batch and then merged, where each batch corresponds to a specific sample), 5) scaling of the features to unit variance and zero mean (*scale* function with max_value equal to 10), 6) application of scAEspy on the HVG space by considering the raw expression (i.e., counts) (Tangherloni et al., 2019), 7) batch correction by sample applying BBKNN algorithm (v.1.3.6, *bbkkn* function with use_faiss equal to false, approx equal to false and the Euclidean distance) to the latent space (16 components) generated by the used AE, 8) Leiden algorithm (*leiden* function with resolution equal to 2.2) applied to the neighborhood graph generated by BBKNN. The 27 obtained clusters were manually annotated by considering the merged data using well-known cell-type specific genes and the Differentially Expressed Genes (DEGs). DEGs were computed by using *rank_genes_groups* function (Wilcoxon rank-sum with adjusted p values for multiple testing with the Bonferroni correction), which compares each cluster to the union of the rest of the clusters. The clusters that either did not express specific cell type genes or expressed marker genes of different cell types had been iteratively subclustered. Specifically, we applied the Leiden algorithm (*leiden* function with resolution equal to 0.5) to subcluster Endothelial cells, obtaining four distinct clusters: the first two clusters have been annotated as Monocytes 2, the third as NK cells and the fourth as Endothelial cells. Finally, we used the Leiden algorithm (*leiden* function with resolution equal to 0.5) to cluster the Unspecified cluster getting four clusters. We merged three clusters with the HSC/MPP cluster while one was annotated as Unspecified.

Dimensionality reduction of scRNA-Seq data

After the detection of the first 1,000 HVGs, we applied scAEspy to HVG space by setting alpha and lambda equal to 0 and 2, respectively, in order to obtain the Gaussian Mixture Maximum Mean Discrepancy Variational AE (GMMMDVAE) (Tangherloni et al., 2019). We run GMMMDVAE for 100 epochs with a batch size equal to 100, one hidden layer of 64 neurons, a latent space of 16 neurons, 15 Gaussian distributions, learnable prior distribution, constrained Poisson loss function, and sigmoid activation function. Then, we applied BBKNN to the latent space (16 components) to generate the neighborhood graph by identifying top neighbors of each cell in each batch separately. We applied UMAP (v.0.3.10, SCANPY *umap* function with random_state equal to 8 and n_components equal to 3) to the obtained neighborhood graph.

Trajectory analysis of scRNA-Seq data

In order to perform a detailed comparison among different trajectory modeling tools, the Dynverse tool (Saelens et al., 2019) was used. Based on the scoring system provided by Dynverse and following a careful inspection of the generated trajectories, we applied PAGA and Force-Directed Graph (FDG) to infer the development trajectories. We removed the endothelial cells and recalculated the neighborhood graph (*neighbors* function with n_neighbors equal to 30) on the latent space (16 components) to exploit the data before batch correction (Luecken and Theis, 2019). We computed the PAGA graph (*paga* function with model equal to v1.2) and the Force-Atlas2 (FA2) using PAGA-initialization (*draw_graph* function, which exploits the FA2 class from fa2 (v.0.3.5) Python package, using the HSC/MPP cluster as root and maxiter equal to 1,000).

Differential expression analysis

Following cluster annotation, we performed biologically-relevant pairwise DE tests between pairs of clusters to identify DEGs and to examine the quantitative changes in the expression levels between the clusters. Specifically, we tested MPPs against MPPs-Cycle, MEMPs against MEMPs-Cycle, MPPs against MEMPs, MPPs against LMPs, MPPs against GPs, and MEMPs against LMPs. In order to cope with the unbalanced distributions between two groups of cells, due to the different number of cells in each cluster, we used the following subsampling strategy. Given two groups of cells, the biggest group was randomly subsampled taking a number of cells equal to the number of cells composing the smallest group. For each gene, a two-sided t test for the means of two independent samples (i.e., biggest group and subsampled one) was applied. We used the *ttest_ind* function (equal_var equal to false) provided by the Python SciPy (Virtanen et al., 2020) package (v.1.4.1). Since we did not assume that the two groups have identical variances, the Welch's t test was automatically applied. Then, we calculated the median of the p values of these t tests. We applied this subsampling strategy 1001 times and calculated the median of the medians to select the subset of the biggest group to run the DE analysis.

For a given subset of cells from the biggest group and the smallest one, we calculated the DEGs by applying the *rank_genes_groups* function (Wilcoxon rank-sum with adjusted p values for multiple testing with the Benjamini-Hochberg correction). Then, we filtered out the obtained DEGs by using the *filter_rank_genes_groups* function (min_in_group_fraction equal to 0.3 and max_out_group_fraction equal to 1, so that a gene is expressed in at least 30% of the cells in one of the two tested groups; min_-

fold_change equal to 0). Following the aforementioned workflow, we compared cells from the liver and femur from the same cluster. Finally, we analyzed HSC/MPPs and HSC/MPPs-Cycle to see which genes contributed to the observed difference between cells from femur and liver.

We also carried out a DE test to compare the expression of cell surface proteins in HSC/MPPs from femur and liver cells. As a first step, we selected 1) genes that encode CD molecules, 2) transmembrane genes available in CellPhoneDB (Efremova et al., 2020), and 3) genes that encode plasma membrane proteins from Uniprot (key KW-1003). Then, we applied the subsampling strategy comparing HSC/MPPs from femur and liver cells. Finally, we calculated the DEGs by considering only the genes that are expressed in at least 30% of the cells in one of the two tested groups.

Differentiation pathway analysis

We performed a gene-set enrichment analysis, using pathway databases, comparing liver and femur HSC/MPP cells. First, we calculated DEGs by comparing liver and femur applying the strategy described above. Then, we used g:Profiler (Raudvere et al., 2019) focusing on Gene Ontology (GO) terms, Kyoto Encyclopedia of Genes and Genomes (KEGG), and Reactome. Specifically, we applied the profile function provided by the Python *GProfiler* package for both liver and femur cells. As a query set, we used the liver (or femur) DEGs while as background we used the genes that are expressed in at least 30% of liver (or femur) cells. We also set the following parameters required by the *profile* function: *organism* equal to *Homo sapiens*; *sources* equal GO terms, KEGG, and Reactome; *domain_scope* equal to custom_annotated; *significance_threshold_method* (i.e., the correction method for the p values) equal to bonferroni; *user_threshold* (i.e., the threshold for the corrected p values) equal to 0.01.

Cell type classification

We trained both a Random Forest classifier (Pedregosa et al., 2011) and a DNN to predict the cell types by considering the top 5, 10, 20, 30, 50, and 100 marker genes for each cluster using the *log*-normalized expression. Since some marker genes are shared among the clusters, we considered them only once to avoid duplicated columns in the feature matrices. We merged the following clusters: HSC/MPPs and HSC/MPPs-Cycle as HSC/MPPs, MEMPs and MEMPs-Cycle as MEMPs, Granulocytes 1, Granulocytes 2, and Granulocytes 3 as Granulocytes; pDCs and pDCs-Cycle as pDCs; CD4+ Monocytes, Monocytes, and Monocyte Prog as Monocytes; Pre-B cells, Pro-B cells, and Mature B cells as B cells. Thus, we obtained 14 distinct clusters.

We used the *RandomForestClassifier* (n_estimators equal to 100 and Gini criterion) provided by Scikit-learn (Pedregosa et al., 2011) (v.0.21.2). We developed the DNN by using Keras (v.2.2.4; https://keras.io) with Tensorflow (Abadi et al., 2016) (v.1.12.0) as backend. The network is composed of 2 dense hidden layers of 64 and 32 neurons, respectively. We added a dropout (50%) layer before the first layer as well as a dropout (30%) layer before the second layer. We trained the DNN for 1,000 epochs using the Adam optimizer (Kingma and Ba, 2014) by minimizing the categorical cross-entropy loss function. We also set an early stopping with 100 epochs as patience to avoid overfitting.

We applied a stratified 10-fold cross-validation (Scikit-learn *StratifiedKFold* function) resampling procedure to evaluate both the Random Forest and DNN. The Random Forest achieved the best result when the top 30 marker genes per cluster were used (mean accuracy equal to 88.54% and standard deviation equal to 1.03%), while the DNN considering the top 30 (mean accuracy equal to 90.40% standard deviation equal to 1.31%) and 50 marker genes per cluster (90.25% and standard deviation equal to 0.98%).

As a further test, we evaluated the ability of our DNN to generalize on unseen data. We split the dataset into a train set (80%) and a test set (20%) (Scikit-learn *train_test_split* function with test_size equal to 0.2). We then divided the train set into a train set (85%) and a validation set (15%, (*train_test_split* function with test_size equal to 0.15). We trained our DNN with the train set, validating it using the validation set. When we took into account the top 30 marker genes, we achieved an accuracy equal to 91.50% on the validation set. When considering the top 50 marker genes the accuracy was 91.68%. Finally, we predicted the labels of the test set by obtaining an accuracy equal to 90.46% (30 marker genes) and 90.23% (50 marker genes).

Upstream analysis of scATAC-Seq data

We performed the upstream analysis using the samtools (Li et al., 2009) (v1.9), bedtools (Quinlan, 2014) (v2.27.1), Picard tools (v2.9.0; http://broadinstitute.github.io/picard/) and BWA (Li and Durbin, 2009) (v0.7.17). First, we aligned fastq files to the GRCh38 reference genome (average 473,886 reads per cell), followed by marking duplicates with *MarkDuplicates* function from Picard tools and removing duplicates using samtools *view* with -F 1804 parameter per each cell. Overall with average duplicates rate 77% we obtained 91,554 reads per cell after removing duplicates. Next, we transformed bam files to bed files using *bamtobed* bedtools function in bedpe mode and kept only fragments that are no bigger than 1000 bp using a custom script. We called peaks (for the clusters with more than 50 cells) using the SnapATAC approach (Fang et al., 2019) with macs2 (Zhang et al., 2008) parameters "–nomodel–shift 100–ext 200–qval 5e-2 -B" and obtained 152,283 peaks. Importantly, for the downstream analysis in R, we binarized counts per cell using Signac (Stuart et al., 2019; https://github.com/timoast/signac/) *BinarizeCounts* function, resulting in 32,217 fragments per cell on average.

Downstream analysis of scATAC-Seq data

The downstream analysis was done in R 3.6.1 applying Seurat (Butler et al., 2018; Stuart et al., 2019) (v3.1.4), Signac (v0.2.4), chromVAR (Schep et al., 2017) (v.1.8) and Harmony (Korsunsky et al., 2019) (v1.0). The pipeline included a QC step (duplicates removal, number of fragments, fragments per peak, fraction of reads mapping to blacklist regions, nucleosome signal, and transcriptional start site (TSS) enrichment), application of LSI dimensionality reduction to the three samples independently (*RunTFIDF* function with method equal to 2, *FindTopFeatures* function setting min.cutoff to q0, and *RunSVD* function using the peaks as assay), batch correction by sample, lane, and organ applying Harmony on the first 50 LSI components, excluding the first one, (*RunHarmony* function setting assay.use to peaks, max.iter.harmony to 20, max.iter.cluster to 200, sigma to 0.25, and theta to 2, 4, 4 in order to weight more the batch related to samples). TF activities on the ATAC-seq data were calculated using the Signac implementation of chromVAR using the *RunChromVAR* function taking as tested motifs dataset from HOCOMOCO (Kulakovskiy et al., 2018) v11 human TF binding models database (769 TFs).

Dimensionality reduction of scATAC-Seq data

We applied the UMAP algorithm to the first 50 LSI components corrected by Harmony (*RunUMAP* function with umap.method equal to uwot and n.neighbors equal to 10, *FindNeighbors* function setting annoy.metric to cosine). We identified seven distinct clusters by using the Seurat function *FindClusters* (resolution equal to 0.5).

Trajectory analysis of scATAC-Seq data

We inferred the development trajectories by applying PAGA and FDG. We recalculated the neighborhood graph using the SCANPY *neighbors* functions (n_neighbors equal to 30) on the 50 LSI components corrected by Harmony. We computed the PAGA graph (SCANPY *paga* function with model equal to v1.0) and used it to initialise the FA2 algorithm (SCANPY *draw_graph* function using cluster 1 as root and maxiter equal to 1,000).

Integration of scRNA-Seq and scATAC-Seq data

We integrated scRNA-Seq and scATAC-Seq data using a recently developed method by Stuart et al. (2019). Namely, we used our scRNA-Seq data as reference dataset to train the classifier and automatically assign a cell type to each scATAC-Seq cell. The training of the classifier was performed using 511 CD34+ CD38- cells from our scRNA-Seq experiment. In order to have a suitable number of cells for each cell type to train the classifier, we considered scRNA-Seq clusters with at least 20 cells (i.e., HSC/MPPs, HSC/MPPs-Cycle, MEMPs, MEMPs-Cycle, GPs, and LMPs). We generated a gene expression matrix from our scATAC-Seq dataset by assigning each peak to the gene by considering the genome coordinates of the gene body ± 3 kb. We applied the Seurat function *FindTransferAnchors* (query.assay equal to RNA_promoter, features equal to the counts of the RNA_promoter, and k.anchor equal to 6) on the Canonical Correlation Analysis (CCA) space because it was more suitable, compared to the LSI space, for capturing the shared feature correlation structure between scRNA-Seq and scATAC-Seq data. We assigned the cell types to the scATAC-Seq cells by applying the Seurat *TransferData* on the first 50 LSI components corrected by Harmony considering the calculated anchors (refdata equal to the six scRNA-Seq clusters). In order to avoid assignments based on a low score, all cells with the prediction score lower than 40% (the value of a uniform distribution of six clusters is 16,67%) were labeled as unknown.

Transcription factor regulons prediction

To run SCENIC workflow on our raw scRNA-Seq data, we used an in-house constructed Snakemake pipeline via combining Arboreto package GRNBoost2 and SCENIC algorithms with default parameters. To predict transcription factor regulons, we used human v9 motif collection, as well as both *hg38__refseq-r80__10kb_up_and_down_tss.mc9nr.feather* and *hg38__refseq-r80__500bp_up_and_100bp_down_tss.mc9nr.feather* databases from the cisTarget (https://resources.aertslab.org/cistarget/). The resulting AUC scores per each cell and adjacency matrix were used for downstream analysis and visualization.

Isolation of human fetal MSCs

Human primary fMSCs were isolated from the femur of a 19 pcw sample following an established protocol used for mouse bones (Perpétuo et al., 2019). Briefly, the bone was rinsed in PBS and the bone epiphyses cut with a scalpel. The bone marrow was flushed with 50 mL PBS, centrifuged at 300 g for 5 minutes, resuspended in alphaMEM medium (Thermo Fisher Scientific) supplemented with 2 mM L-glutamine (Thermo Fisher Scientific), 100 U/ml penicillin/streptomycin (Thermo Fisher Scientific) and 10% fetal bovine serum (Sigma) at a concentration of $5x10^6$ cells/ml and cultured at 37°C at 5% CO_2. After 24 hours, floating cells were removed by washing twice with PBS and medium was changed twice a week until the culture was 70% confluent. Cells were cryopreserved until use.

Single-cell *in vitro* culture

Single Lin- CD34+ CD38- CD62L+ CD52+ CD133+ cells, isolated from the fetal bone marrow of three different fetuses (20-22 pcw), were index-sorted into 96-well plates seeded with fMSCs or MS5 (obtained by DSMZ) and supplemented with cytokines as previously described (Velten et al., 2017). Cells were cultured for 15 days at 37°C at 5% CO2. At the end of the culture, colonies were filtered to exclude feeder layer cells, and their lineage output was assessed by the expression of CD41a (megakaryocytic-Mk), CD235a (erythroid-Ery), CD3/CD56 (lymphoid-Ly), and CD11b (myeloid-My) by flow cytometry using a BD LSR-Fortessa analyzer. Colonies were considered positive for a lineage if $\geq$ 30 cells were detected in the relative gate.

Cell cycle analysis

Cells from the fetal liver and the bone marrow were stained with cell-surface antibodies, fixed and permeabilised for 20 minutes at 4°C using the Cytofix/cytoperm kit (BD Biosciences). Cells were then stained with FITC-MKi67 antibody (BD Biosciences) overnight at 4°C and finally with DAPI prior to flow cytometry acquisition. Cell cycle phases were defined as follows: G0 (Mki67-DAPI-), G1 (Mki67+DAPI-), S-G2-M (Mki67+DAPI+).

QUANTIFICATION AND STATISTICAL ANALYSIS

Differences across cell types

In order to assess qualitative and quantitative differences between the hematopoietic cells collected from the liver and femur, we implemented different statistical tests. For each cluster, we calculated if there is a statistically significant difference in the number of cells (Test 1), the number of expressed genes per cell (Test 2), and the cell cycle state of blood cells collected from liver and femur (Test 3).

Test 1. Since we used different gates to sort cells and we sorted a different number of cells in each experiment, we first normalized the number of cells from liver and femur. We selected only the matched gates (i.e., the gates where we sorted hematopoietic cells from both liver and femur). Then, we selected cells from the liver (or femur) from each gate in each of the clusters. For each cluster, we normalized the number of cells inside the cluster in the range [0, 100] by dividing the number of cells for the total number of cells of the gate in order to obtain a number of cells equal to 100. Next, for each cluster, we calculated the median of the cells in the liver (or femur) among the different gates. In order to evaluate if there is a statistically significant difference between the number of cells in the liver and femur considering all the clusters, we applied the ChiSq test by normalizing the distributions (i.e, the median of the gates of each cluster) of the cells from liver and femur among the clusters. We applied the *chi2_contingency* function provided by the Python SciPy. Since the obtained p value is equal to 1.02×10^{-4}, we applied Fisher's exact test (SciPy *fisher_exact* function) to each cluster to find which clusters contributed to the difference.

Test 2. In this test, we evaluated the number of expressed genes between cells collected from femur and liver. In order to remove possible technical effects for each cell, we divided the number of expressed genes by the number of reads uniquely mapped against the reference genome. For each cluster, we applied both the KS test (SciPy *ks_2samp* function) and the MWW test (SciPy *mannwhitneyu* function). Since the number of cells from femur and liver is very different in any given cluster (giving rise to unbalanced distributions) we used a subsampling strategy similar to that used for the DE analysis. We randomly subsampled the biggest group 1,001 times taking a number of cells equal to the number of cells composing the smallest group. We applied the KS (and MWW) test comparing the smallest group to the subsampled ones obtaining a distribution of p values. Finally, we calculated the median of this distribution of p values to evaluate if there is a statistically significant difference between the number of expressed genes in the cells from the liver and femur. Note that we excluded the clusters where the number of the cells from femur or liver was lower than 20.

Test 3. For each cluster, we compared G2M/S and G1 states by normalizing the number of cells from the liver and femur in the two states. We applied Fisher's exact test (SciPy *fisher_exact* function) to each cluster to find a possible statistically significant difference between the number of cells in G2M/S and G1 states in the liver and femur.

Cell Stem Cell, Volume *28*

Supplemental Information

Integrative Single-Cell RNA-Seq and ATAC-Seq Analysis of Human Developmental Hematopoiesis

Anna Maria Ranzoni, Andrea Tangherloni, Ivan Berest, Simone Giovanni Riva, Brynelle Myers, Paulina M. Strzelecka, Jiarui Xu, Elisa Panada, Irina Mohorianu, Judith B. Zaugg, and Ana Cvejic

Integrative Single-cell RNA-Seq and ATAC-Seq Analysis of Human Developmental Haematopoiesis

Anna Maria Ranzoni, Andrea Tangherloni, Ivan Berest,
Simone Giovanni Riva, Brynelle Myers, Paulina M. Strzelecka,
Jiarui Xu, Elisa Panada, Irina Mohorianu,
Judith B. Zaugg, Ana Cvejic

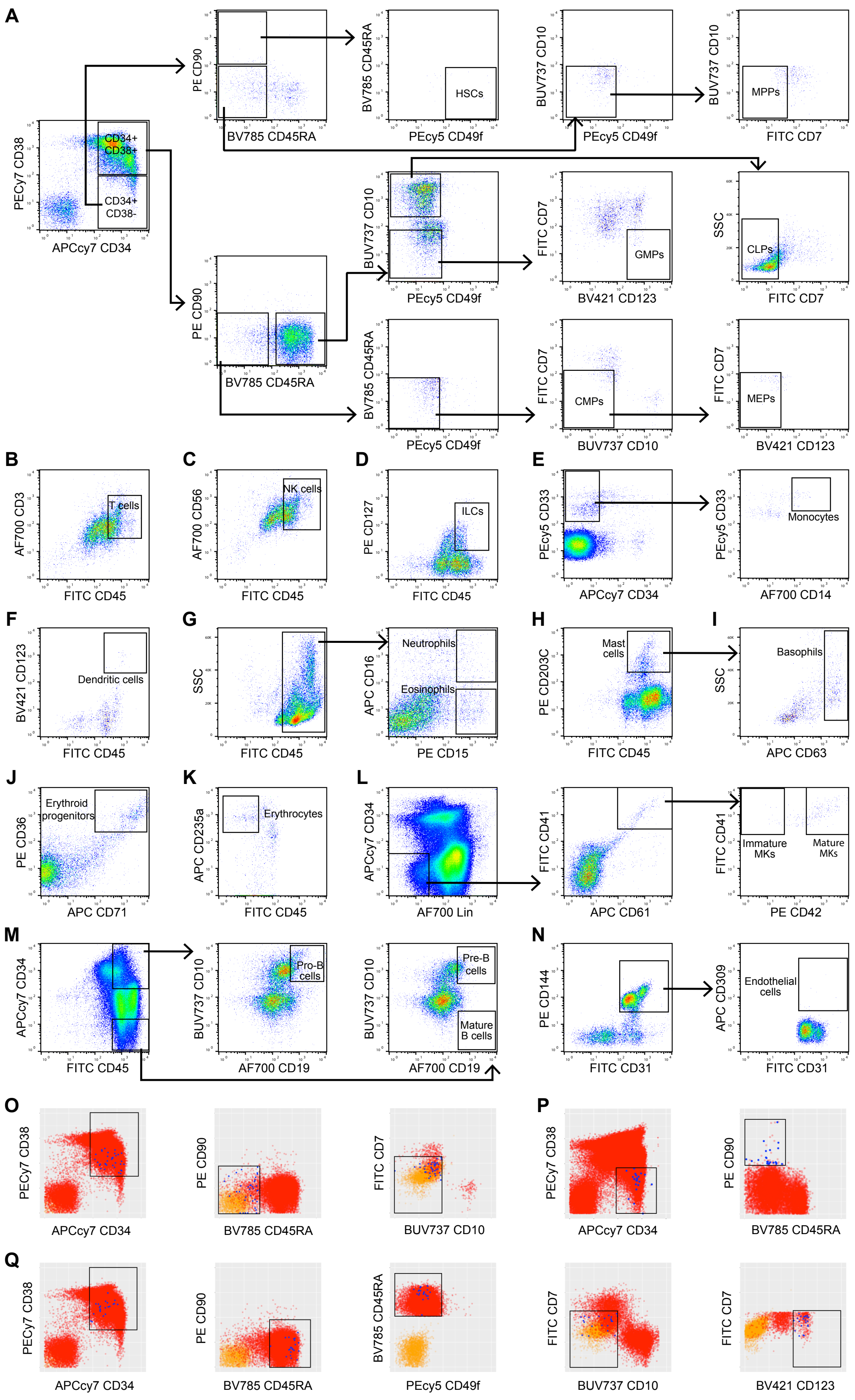
A
CD34+ CD38+
CD34+ CD38-
PECy7 CD38
APCcy7 CD34
PE CD90
BV785 CD45RA
HSCs
PEcy5 CD49f
BUV737 CD10
MPPs
FITC CD7
GMPs
BV421 CD123
SSC
CLPs
CMPs
MEPs
B
AF700 CD3
T cells
FITC CD45
C
AF700 CD56
NK cells
D
PE CD127
ILCs
E
PEcy5 CD33
Monocytes
AF700 CD14
F
BV421 CD123
Dendritic cells
G
Neutrophils
Eosinophils
APC CD16
PE CD15
H
PE CD203C
Mast cells
I
Basophils
APC CD63
J
PE CD36
Erythroid progenitors
APC CD71
K
APC CD235a
Erythrocytes
L
APCcy7 CD34
AF700 Lin
FITC CD41
APC CD61
Immature MKs
Mature MKs
PE CD42
M
Pro-B cells
Pre-B cells
Mature B cells
AF700 CD19
N
PE CD144
FITC CD31
APC CD309
Endothelial cells
O
P
Q

Supplementary figure 1 – Sorting panels, Related to Figure 1, related to Figure 1. A-N. FACS sorting panel and gating strategy for the isolation of phenotypically defined cell types: **A.** Committed and non-committed haematopoietic progenitors, HSCs, MPPs, CMPs, GMPs, MEPs, and CLPs. **B.** T cells. **C.** NK cells. **D.** ILCs. **E.** Monocytes. **F.** Dendritic cells. **G.** Neutrophils and eosinophils. **H.** Mast cells. **I.** Basophils. **J.** Erythroid progenitors. **K.** Erythrocytes. **L.** Immature and mature MKs. **M.** Pro-B cells, pre-B cells, and mature B cells. **N.** Endothelial cells. **O-Q.** Index sorting data FACS plots showing sorted cells (blue), total gated population (red), and unstained population (yellow) for defined cell types: **O.** CMPs. **P.** HSCs. **Q.** GMPs. HSCs - haematopoietic stem cells, MPPs - multipotent progenitors, CMPs - common myeloid progenitors, GMPs - granulocyte-monocyte progenitors, MEPs - megakaryocyte-erythroid progenitors and CLPs - common lymphoid progenitors, NK cells - natural killer cells, ILCs - innate lymphoid cells, MKs - megakaryocytes.

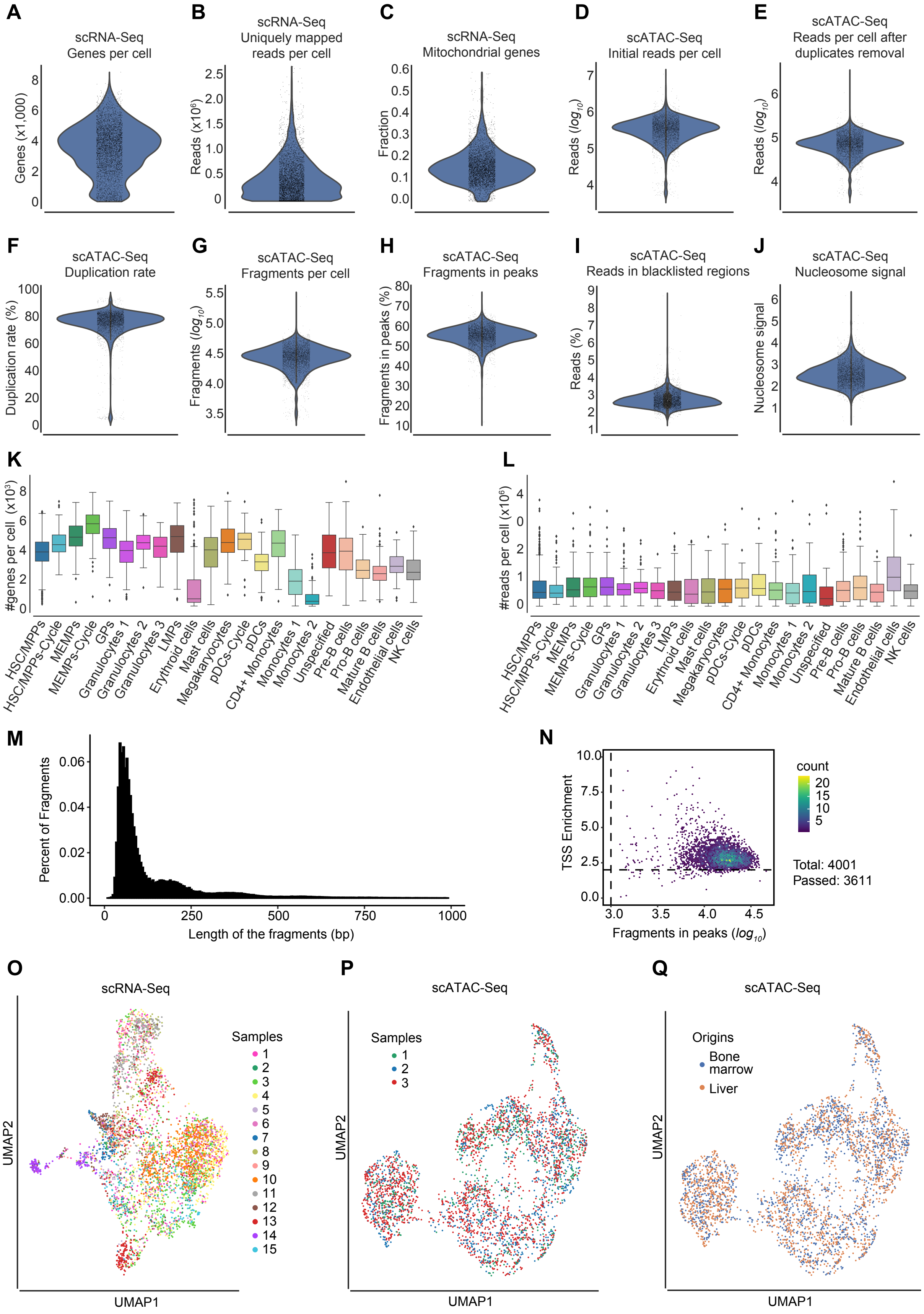
A
scRNA-Seq
Genes per cell
Genes (x1,000)
B
scRNA-Seq
Uniquely mapped
reads per cell
Reads (x10⁶)
C
scRNA-Seq
Mitochondrial genes
Fraction
D
scATAC-Seq
Initial reads per cell
Reads (log10)
E
scATAC-Seq
Reads per cell after
duplicates removal
Reads (log10)
F
scATAC-Seq
Duplication rate
Duplication rate (%)
G
scATAC-Seq
Fragments per cell
Fragments (log10)
H
scATAC-Seq
Fragments in peaks
Fragments in peaks (%)
I
scATAC-Seq
Reads in blacklisted regions
Reads (%)
J
scATAC-Seq
Nucleosome signal
Nucleosome signal
K
#genes per cell (x10³)
L
#reads per cell (x10⁶)
HSC/MPPs
HSC/MPPs-Cycle
MEMPs
MEMPs-Cycle
GPs
Granulocytes 1
Granulocytes 2
Granulocytes 3
LMPs
Erythroid cells
Mast cells
Megakaryocytes
pDCs-Cycle
pDCs
CD4+ Monocytes
Monocytes 1
Monocytes 2
Unspecified
Pre-B cells
Pro-B cells
Mature B cells
Endothelial cells
NK cells
M
Percent of Fragments
Length of the fragments (bp)
N
TSS Enrichment
Fragments in peaks (log10)
count
Total: 4001
Passed: 3611
O
scRNA-Seq
Samples
UMAP1
UMAP2
P
scATAC-Seq
Samples
Q
scATAC-Seq
Origins
Bone marrow
Liver

Supplementary figure 2 – Quality control and batch effects correction in scRNA-Seq and scATAC-Seq data, related to Figures 1 and 3. A. Violin plots showing the number of expressed genes per cell in scRNA-Seq data. **B.** Violin plots showing the number of uniquely mapped reads against the reference genome per cell in scRNA-Seq data. **C.** Violin plots showing the fraction of mitochondrial genes compared to all genes per cell in scRNA-Seq data. **D.** Violin plots showing the number of reads per cell, prior to duplicates removal, in scATAC-Seq data. The y-axis is in log_{10} scale. **E.** Violin plots showing the number of reads per cell after duplicates removal in scATAC-Seq data. The y-axis is in log_{10} scale. **F.** Violin plots showing the duplicate rate in scATAC-Seq data. **G.** Violin plots showing the number of fragments per cell in scATAC-Seq data. The y-axis is in log_{10} scale. **H.** Violin plots showing the percentage of fragments per peak in scATAC-Seq data. **I.** Violin plots showing the percentage of reads mapping to the blacklist regions in scATAC-Seq data. **J.** Violin plots showing the nucleosome signal per cell in scATAC-Seq data. **K.** Box plot showing the number of genes per cell in each identified cell type in scRNA-Seq data. **L.** Box plot showing the number of uniquely mapped reads per cell in each identified cell type in scRNA-Seq data. M. Histogram showing the length of the fragments in terms of base pairs (200 bins). **N.** Scatterplot showing the fragments in peaks with respect to TSS enrichment. The colour intensity represents the number of counts. The x-axis is in log_{10} scale. **O.** UMAP visualization of the scRNA-Seq samples ($n = 15$) after the batch effect correction with BBKNN. Each colour represents a different sample. **P.** UMAP visualization of the scATAC-Seq samples ($n = 3$) after the batch effect correction with Harmony. Each colour represents a different sample. **Q.** UMAP visualization of the scATAC-Seq bone marrow (blue) and liver (orange) CD34+ CD38- cells after the batch effect correction with Harmony.

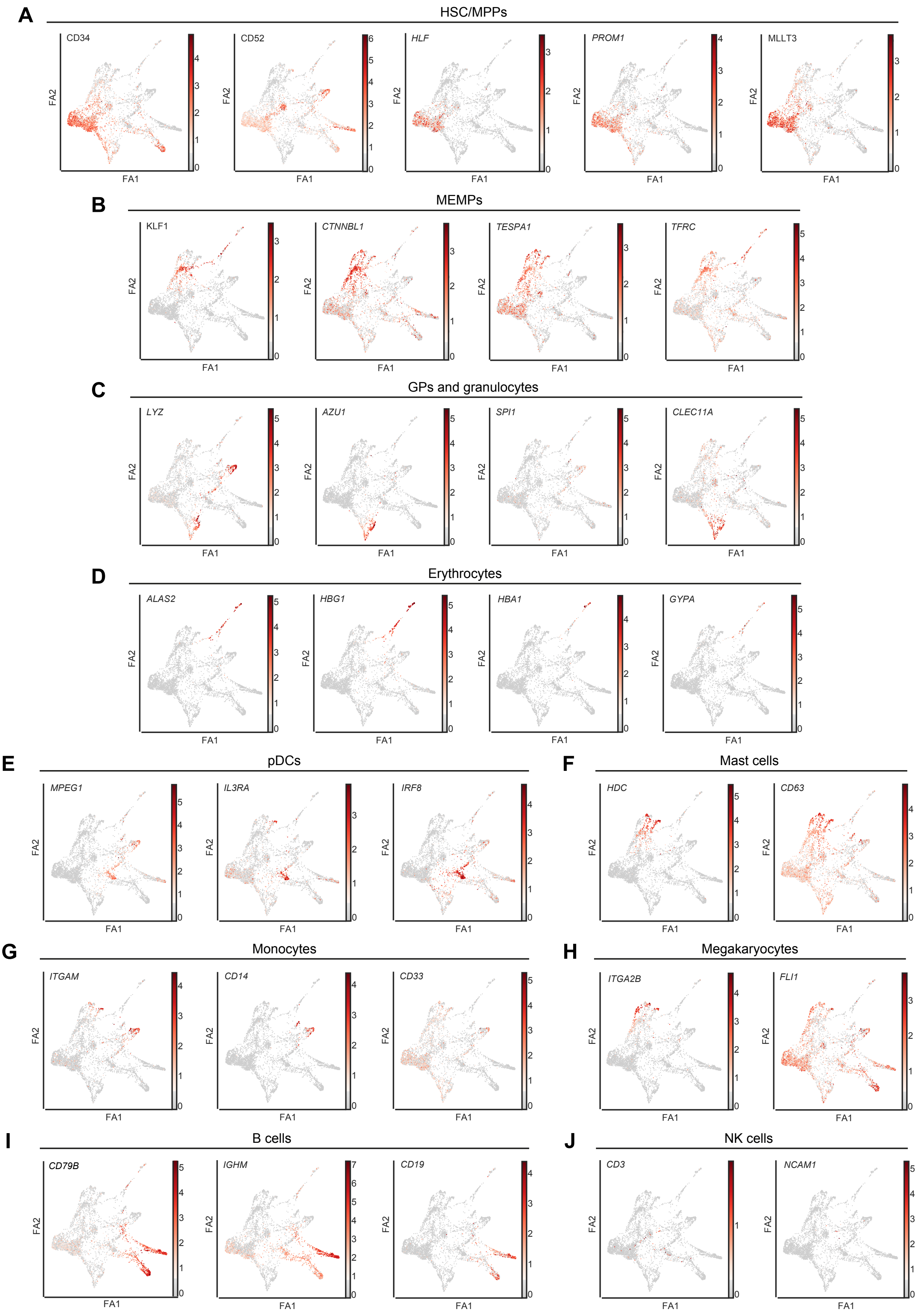

A
HSC/MPPs
CD34
CD52
HLF
PROM1
MLLT3
B
MEMPs
KLF1
CTNNBL1
TESPA1
TFRC
C
GPs and granulocytes
LYZ
AZU1
SPI1
CLEC11A
D
Erythrocytes
ALAS2
HBG1
HBA1
GYPA
E
pDCs
MPEG1
IL3RA
IRF8
F
Mast cells
HDC
CD63
G
Monocytes
ITGAM
CD14
CD33
H
Megakaryocytes
ITGA2B
FLI1
I
B cells
CD79B
IGHM
CD19
J
NK cells
CD3
NCAM1
FA1
FA2

Supplementary figure 3 – Expression of top marker genes along the differentiation trajectory, related to Figure 2. (A-J) FDG visualisation of the log-normalised gene expression of marker genes along the differentiation trajectory. **A.** HSC/MPPs (*CD34*, *CD52*, *HLF*, *PROM1*, and *MLLT3*). **B.** MEMPs (*KLF1*, *CTNNBL1*, *TESPA1*, and *TFRC*). **C.** GPs and granulocytes (*LYZ*, *AZU1*, *SPI1*, and *CLEC11A*). **D.** Erythrocytes (*ALAS2*, *HBG1*, *HBA1*, and *GYPA*). **E.** pDCs (*MPEG1*, *IL3RA*, and *IRF8*). **F.** Mast cells (*HDC* and *CD63*). **G.** Monocytes (*ITGAM*, *CD14*, and *CD33*). **H.** Megakaryocytes (*ITGA2B* and *FLI1*). **I.** B cells (*CD79B*, *IGHM*, and *CD19*). **J.** NK cells (*CD3* and *NCAM1*). Force-Directed Graph - FDG; ForceAtlas2 - FA2; HSC/MPPs - haematopoietic stem cells/multipotent progenitors; MEMPs - megakaryocyte-erythroid-mast progenitors; GPs - granulocytic progenitors; pDCs-Cycle - cycling plasmacytoid dendritic cells.

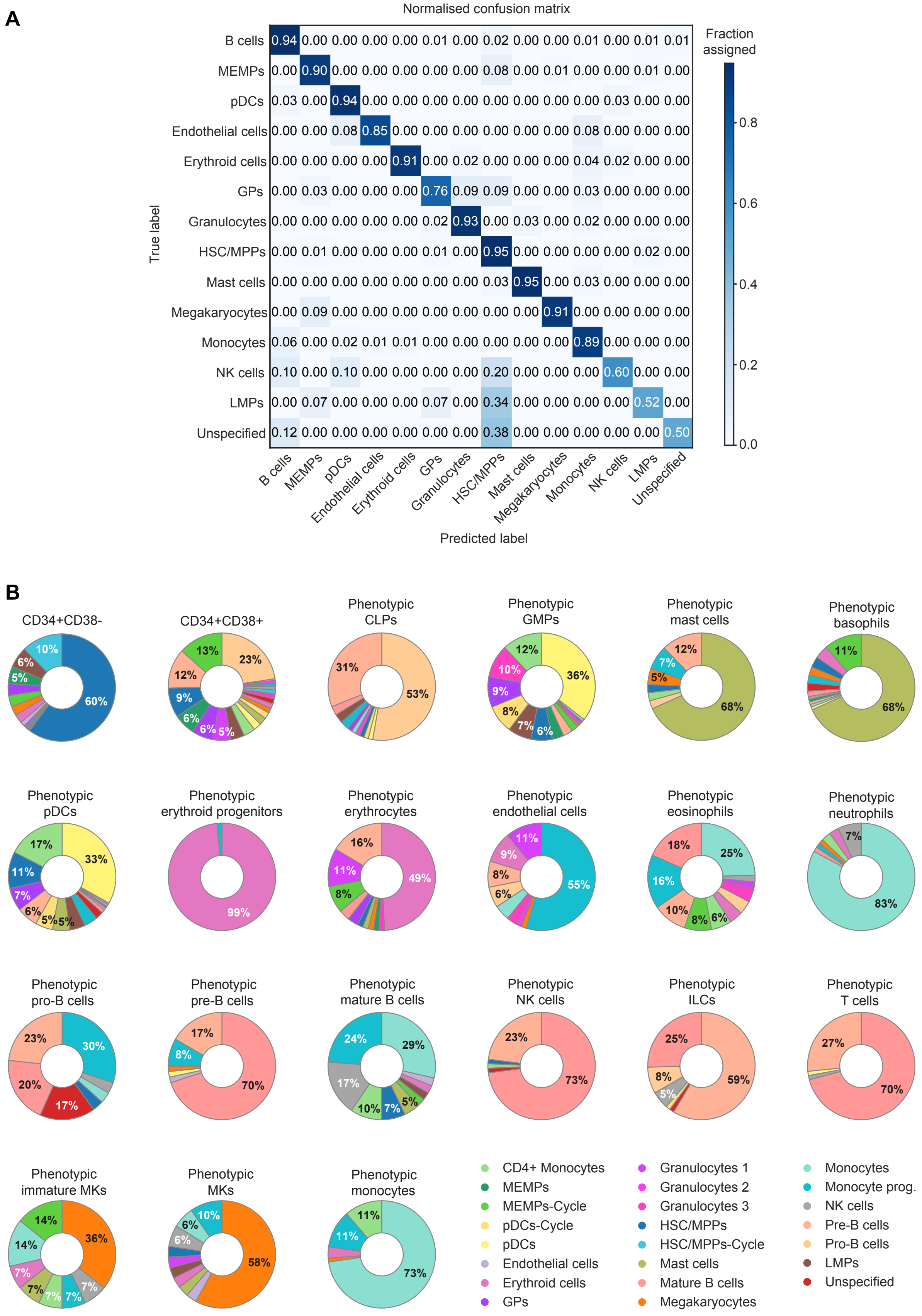
A
Normalised confusion matrix
B cells 0.94 0.00 0.00 0.00 0.00 0.01 0.00 0.02 0.00 0.00 0.01 0.00 0.01 0.01
MEMPs 0.00 0.90 0.00 0.00 0.00 0.00 0.00 0.08 0.00 0.01 0.00 0.00 0.01 0.00
pDCs 0.03 0.00 0.94 0.00 0.00 0.00 0.00 0.00 0.00 0.00 0.00 0.03 0.00 0.00
Endothelial cells 0.00 0.00 0.08 0.85 0.00 0.00 0.00 0.00 0.00 0.00 0.08 0.00 0.00 0.00
Erythroid cells 0.00 0.00 0.00 0.00 0.91 0.00 0.02 0.00 0.00 0.00 0.04 0.02 0.00 0.00
GPs 0.00 0.03 0.00 0.00 0.00 0.76 0.09 0.09 0.00 0.00 0.03 0.00 0.00 0.00
Granulocytes 0.00 0.00 0.00 0.00 0.00 0.02 0.93 0.00 0.03 0.00 0.02 0.00 0.00 0.00
HSC/MPPs 0.00 0.01 0.00 0.00 0.00 0.01 0.00 0.95 0.00 0.00 0.00 0.00 0.02 0.00
Mast cells 0.00 0.00 0.00 0.00 0.00 0.00 0.00 0.03 0.95 0.00 0.03 0.00 0.00 0.00
Megakaryocytes 0.00 0.09 0.00 0.00 0.00 0.00 0.00 0.00 0.00 0.91 0.00 0.00 0.00 0.00
Monocytes 0.06 0.00 0.02 0.01 0.01 0.00 0.00 0.00 0.00 0.00 0.89 0.00 0.00 0.00
NK cells 0.10 0.00 0.10 0.00 0.00 0.00 0.00 0.20 0.00 0.00 0.00 0.60 0.00 0.00
LMPs 0.00 0.07 0.00 0.00 0.00 0.07 0.00 0.34 0.00 0.00 0.00 0.00 0.52 0.00
Unspecified 0.12 0.00 0.00 0.00 0.00 0.00 0.00 0.38 0.00 0.00 0.00 0.00 0.00 0.50
True label
Predicted label
B cells
MEMPs
pDCs
Endothelial cells
Erythroid cells
GPs
Granulocytes
HSC/MPPs
Mast cells
Megakaryocytes
Monocytes
NK cells
LMPs
Unspecified
Fraction assigned
0.0
0.2
0.4
0.6
0.8
B
CD34+CD38-
CD34+CD38+
Phenotypic CLPs
Phenotypic GMPs
Phenotypic mast cells
Phenotypic basophils
Phenotypic pDCs
Phenotypic erythroid progenitors
Phenotypic erythrocytes
Phenotypic endothelial cells
Phenotypic eosinophils
Phenotypic neutrophils
Phenotypic pro-B cells
Phenotypic pre-B cells
Phenotypic mature B cells
Phenotypic NK cells
Phenotypic ILCs
Phenotypic T cells
Phenotypic immature MKs
Phenotypic MKs
Phenotypic monocytes
CD4+ Monocytes
MEMPs
MEMPs-Cycle
pDCs-Cycle
pDCs
Endothelial cells
Erythroid cells
GPs
Granulocytes 1
Granulocytes 2
Granulocytes 3
HSC/MPPs
HSC/MPPs-Cycle
Mast cells
Mature B cells
Megakaryocytes
Monocytes
Monocyte prog.
NK cells
Pre-B cells
Pro-B cells
LMPs
Unspecified

Supplementary figure 4 – Validation of the cell type assignment and transcriptional heterogeneity of phenotypically defined cell populations, related to Figures 1 and 3. A. Confusion matrix showing the cell type assignment achieved by the DNN on the test set (901 cells), considering the top 30 marker genes per cell type (14 distinct cell types). The colour intensity represents the fraction of the assigned cells per cell type. **B.** Donut plots showing the percentage of transcriptionally defined (i.e., manually curated) cell populations in each of the phenotypically defined populations (Expanded from Figure 1C). Each colour represents a different cell type. HSC/MPPs-Cycle - cycling haematopoietic stem cells/multipotent progenitors; HSC/MPPs - haematopoietic stem cells/multipotent progenitors; MEMPs - megakaryocyte-erythroid-mast progenitors; MEMPs-Cycle - cycling megakaryocyte-erythroid-mast progenitors; GPs - granulocytic progenitors; LMPs - lympho-myeloid progenitors; pDCs-Cycle - cycling plasmacytoid dendritic cells; pDCs - plasmacytoid dendritic cells.

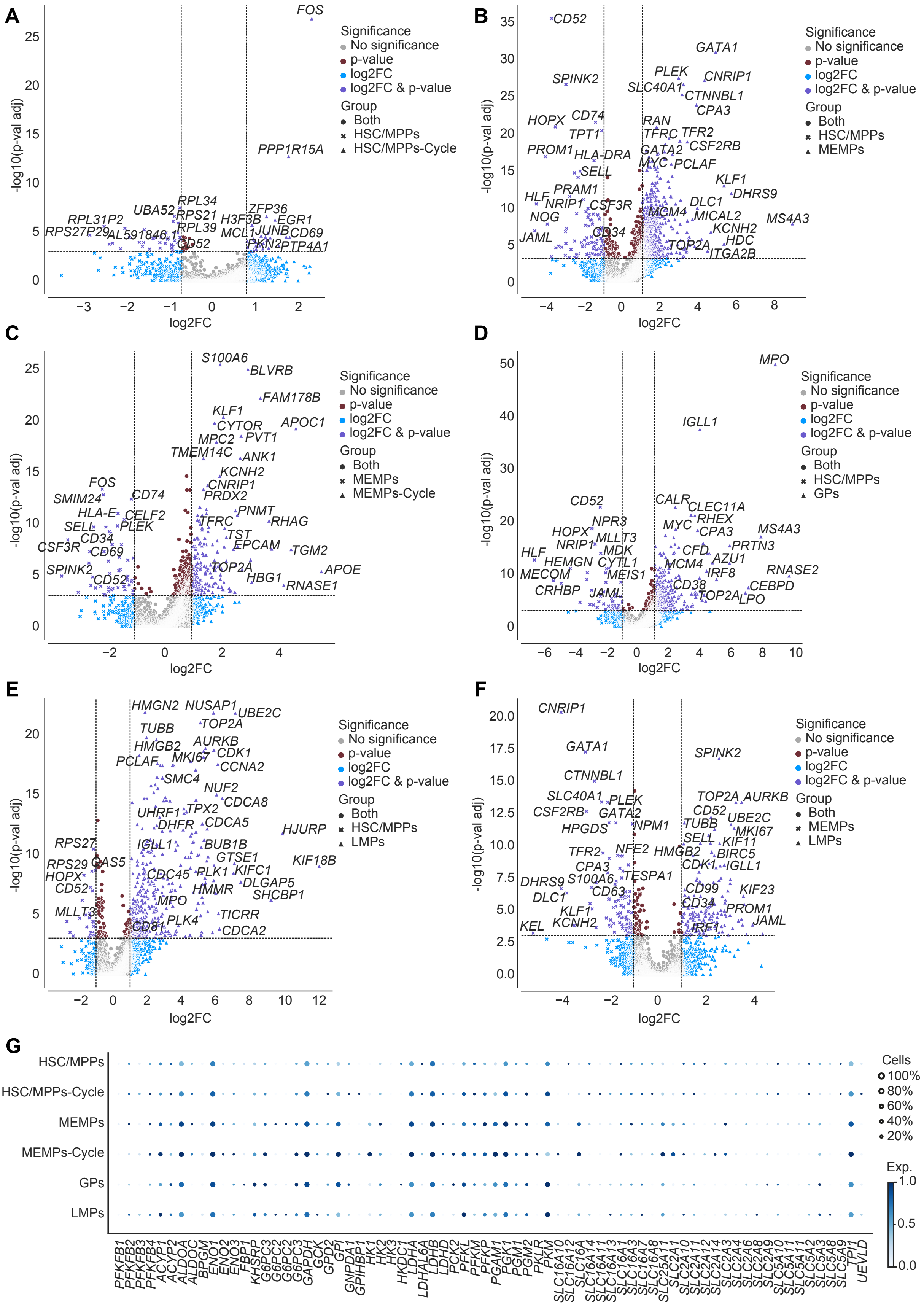

A
FOS
PPP1R15A
RPL34
RPS21
RPL39
UBA52
RPL31P2
RPS27
RPS29
AL591846.1
CD52
H3F3B
ZFP36
EGR1
MCL1
JUNB
CD69
PKN2
PTP4A1
-log10(p-val adj)
log2FC
Significance
No significance
p-value
log2FC
log2FC & p-value
Group
Both
HSC/MPPs
HSC/MPPs-Cycle
B
CD52
GATA1
SPINK2
PLEK
CNRIP1
SLC40A1
CTNNBL1
CPA3
HOPX
CD74
RAN
TPT1
TFRC
TFR2
PROM1
HLA-DRA
GATA2
CSF2RB
MYC
PCLAF
SELL
KLF1
PRAM1
DHRS9
HLF
NRIP1
CSF3R
DLC1
MCM4
MICAL2
NOG
MS4A3
KCNH2
JAML
CD34
HDC
TOP2A
ITGA2B
-log10(p-val adj)
log2FC
Significance
No significance
p-value
log2FC
log2FC & p-value
Group
Both
HSC/MPPs
MEMPs
C
S100A6
BLVRB
FAM178B
KLF1
CYTOR
APOC1
MPC2
PVT1
TMEM14C
ANK1
KCNH2
FOS
CNRIP1
PRDX2
SMIM24
CD74
PNMT
HLA-E
CELF2
RHAG
SELL
PLEK
TFRC
CD34
TST
CSF3R
CD69
EPCAM
TGM2
SPINK2
TOP2A
APOE
CD52
HBG1
RNASE1
-log10(p-val adj)
log2FC
Significance
No significance
p-value
log2FC
log2FC & p-value
Group
Both
MEMPs
MEMPs-Cycle
D
MPO
IGLL1
CD52
CALR
CLEC11A
NPR3
RHEX
HOPX
MLLT3
MYC
CPA3
MS4A3
NRIP1
MDK
CFD
PRTN3
HLF
HEMGN
CYTL1
AZU1
MCM4
RNASE2
MECOM
MEIS1
IRF8
CEBPD
CRHBP
JAML
CD38
TOP2A
LPO
-log10(p-val adj)
log2FC
Significance
No significance
p-value
log2FC
log2FC & p-value
Group
Both
HSC/MPPs
GPs
E
HMGN2
NUSAP1
UBE2C
TUBB
TOP2A
AURKB
HMGB2
CDK1
PCLAF
MKI67
CCNA2
SMC4
NUF2
CDCA8
UHRF1
TPX2
CDCA5
DHFR
HJURP
RPS27
IGLL1
BUB1B
RPS29
CASP5
GTSE1
KIF18B
HOPX
CDC45
PLK1
KIFC1
CD52
DLGAP5
HMMR
SHCBP1
MPO
MLLT3
PLK4
TICRR
CD81
CDCA2
-log10(p-val adj)
log2FC
Significance
No significance
p-value
log2FC
log2FC & p-value
Group
Both
HSC/MPPs
LMPs
F
CNRIP1
GATA1
SPINK2
CTNNBL1
SLC40A1
PLEK
TOP2A
AURKB
CSF2RB
GATA2
CD52
UBE2C
NPM1
TUBB
HPGDS
MKI67
SELL
KIF11
TFR2
NFE2
HMGB2
BIRC5
CPA3
CDK1
IGLL1
DHRS9
S100A6
TESPA1
CD99
KIF23
DLC1
CD63
CD34
KLF1
PROM1
KEL
KCNH2
IRF1
JAML
-log10(p-val adj)
log2FC
Significance
No significance
p-value
log2FC
log2FC & p-value
Group
Both
MEMPs
LMPs
G
HSC/MPPs
HSC/MPPs-Cycle
MEMPs
MEMPs-Cycle
GPs
LMPs
Cells
100%
80%
60%
40%
20%
Exp.
1.0
0.5
0.0
PFKFB1
PFKFB2
PFKFB3
PFKFB4
ACYP1
ACYP2
ALDOA
ALDOC
BPGM
ENO1
ENO2
ENO3
FBP1
KHSRP
G6PC2
G6PC3
G6PC
GAPDH
GCK
GPD2
GPI
GNPDA1
GPIHBP1
HK1
HK2
HK3
HKDC1
LDHA
LDHAL6A
LDHB
LDHD
PCK2
PFKL
PFKM
PFKP
PGAM1
PGK1
PGM1
PGM2
PKLR
PKM
SLC16A10
SLC16A12
SLC16A14
SLC16A13
SLC16A1
SLC16A3
SLC16A7
SLC16A8
SLC25A1
SLC2A10
SLC2A11
SLC2A12
SLC2A14
SLC2A3
SLC2A4
SLC2A6
SLC2A8
SLC2A9
SLC5A10
SLC5A11
SLC5A2
SLC5A3
SLC5A8
SLC5A9
TPI1
UEVLD

Supplementary figure 5 – Differential expression analysis of the progenitor compartment, related to the STAR Methods section. (A-F) Volcano plot showing DEGs between two cell types of interest. **A.** HSC/MPPs and HSC/MPPs-Cycle. **B.** HSC/MPPs and MEMPs. **C.** MEMPs and MEMPs-Cycle. **D.** HSC/MPPs and GPs. **E.** HSC/MPPs and LMPs. **F.** MEMPs and LMPs. The x-axes show the log_2 fold-change (magnitude of change), while the y-axes show the $-log_{10}$ adjusted p-value (statistical significance). We used the Wilcoxon rank-sum with the Benjamini-Hochberg correction. Colours represent the significance of the genes, both in terms of p-value and log_2 fold-change. **G.** Dot plot of the expression of metabolic genes involved in glycolysis in the identified progenitor compartment. The expression of the genes is standardised between 0 and 1. For each gene, the minimum value is subtracted and the result is divided by the maximum. The spot size indicates the percentage of cells that express the gene of interest within each cell type. The colour intensity represents the standardised expression level. HSC/MPPs-Cycle - cycling haematopoietic stem cells/multipotent progenitors; HSC/MPPs - haematopoietic stem cells/multipotent progenitors; MEMPs - megakaryocyte-erythroid-mast progenitors; MEMPs-Cycle - cycling megakaryocyte-erythroid-mast progenitors; GPs - granulocytic progenitors; LMPs - lympho-myeloid progenitors.

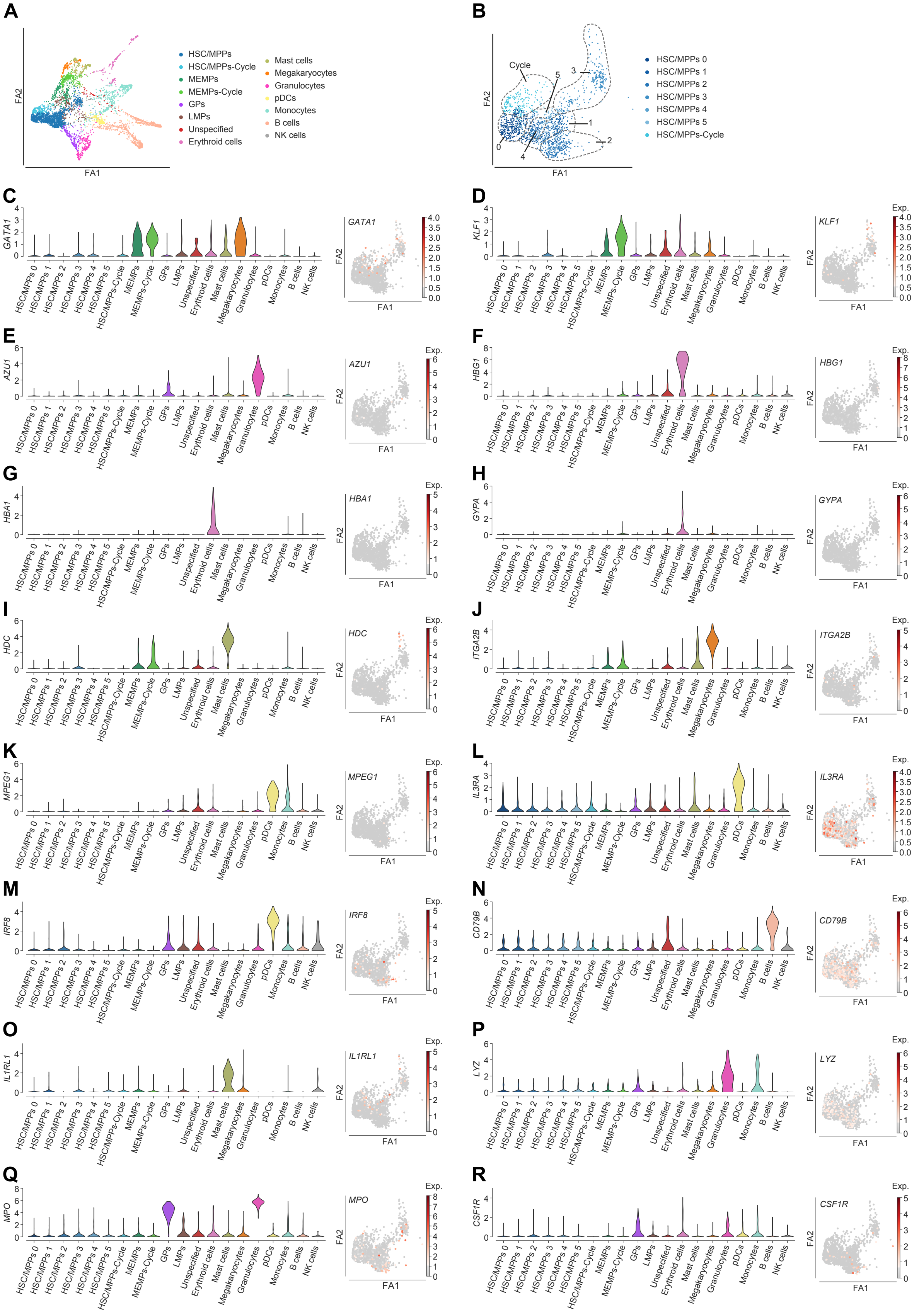

A
FA2
FA1
HSC/MPPs
HSC/MPPs-Cycle
MEMPs
MEMPs-Cycle
GPs
LMPs
Unspecified
Erythroid cells
Mast cells
Megakaryocytes
Granulocytes
pDCs
Monocytes
B cells
NK cells
B
Cycle
HSC/MPPs 0
HSC/MPPs 1
HSC/MPPs 2
HSC/MPPs 3
HSC/MPPs 4
HSC/MPPs 5
HSC/MPPs-Cycle
C
GATA1
D
KLF1
E
AZU1
F
HBG1
G
HBA1
H
GYPA
I
HDC
J
ITGA2B
K
MPEG1
L
IL3RA
M
IRF8
N
CD79B
O
IL1RL1
P
LYZ
Q
MPO
R
CSF1R
Exp.

Supplementary figure 6 – Expression of lineage-specific marker genes in HSC/MPP sub-populations, related to 2. **A.** FDG visualisation of the identified differentiation trajectory. **B.** FDG visualisation of the HSC/MPP sub-populations. **(C-R)** Left panels - violin plots showing the log-normalised median gene expression of lineage-specific marker genes in the HSC/MPP sub-populations and more differentiated haematopoietic cells. Right panels - FDG visualisations of the log-normalised gene expression of the same genes as in violin plots along the differentiation trajectory, considering only the HSC/MPP sub-populations. **C.** *GATA1*. **D.** *KLF1*. **E.** *AZU1*. **F.** *HBG1*. **G.** *HBA1*. **H.** *GYPA*. **I.** *HDC*. **J.** *ITGA2B*. **K.** *MPEG1*. **L.** *IL3RA*. **M.** *IRF8*. **N.** *CD79B*. **O.** *IL1RL1*. **P.** *LYZ*. **Q.** *MPO*. **R.** *CSF1R*. Force-Directed Graph - FDG; ForceAtlas2 - FA2; HSC/MPPs - haematopoietic stem cells/multipotent progenitors; HSC/MPPs-Cycle - cycling haematopoietic stem cells/multipotent progenitors; MEMPs - megakaryocyte-erythroid-mast progenitors; MEMPs-Cycle - cycling megakaryocyte-erythroid-mast progenitors; GPs - granulocytic progenitors; LMPs - lympho-myeloid progenitors; pDCs - plasmacytoid dendritic cells.

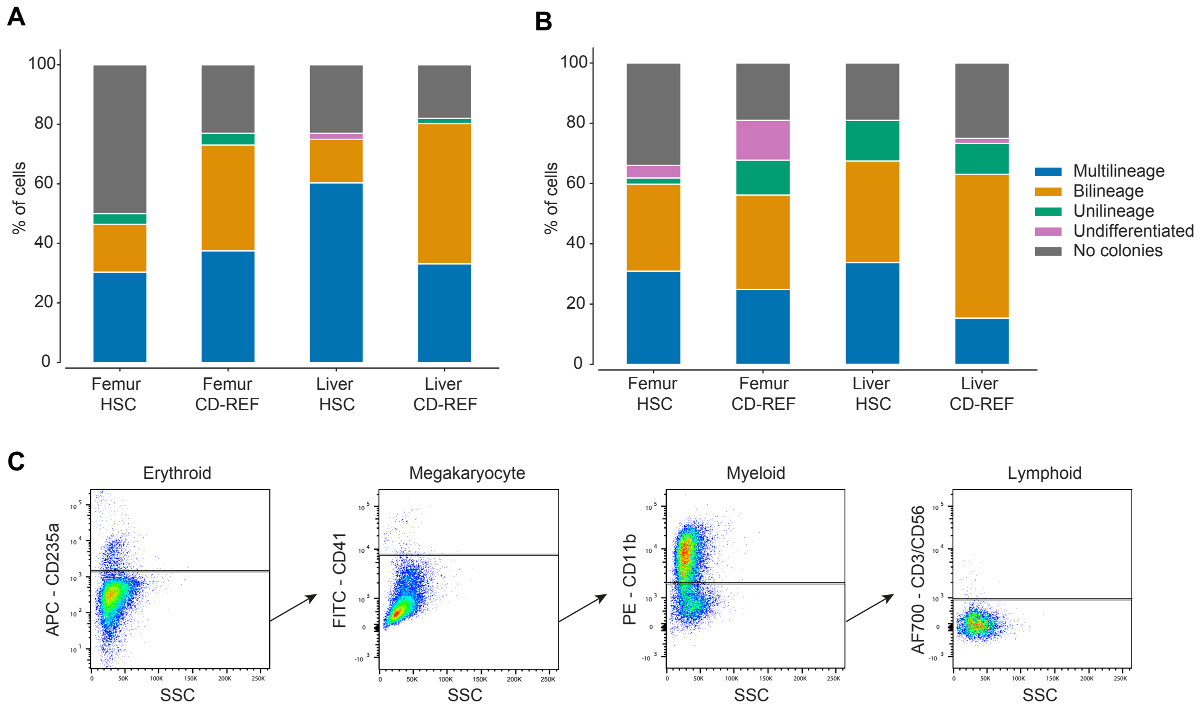

Supplementary figure 7 – Colony formation and the lineage output of CD-REF cells and phenotypic HSCs isolated from the foetal liver and femur, related to Figure 6. A-B. Stacked bar chart showing the differentiation potential and efficiency of colony formation of CD-REF cells compared to the phenotypic HSCs isolated from foetal liver and femur. The y-axis shows the percentage of colonies. The colonies have been divided by their differentiation potential as determined by FACS, namely: multilineage, bilineage, unilineage, and undifferentiated. Data from two different experiments are shown. **C.** Representative FACS plot showing the lineage composition of a quadrilineage colony.

Supplementary table 1 – Cell-surface markers used to isolate cell types, related to Figure 1.

Phenotypic cell type	Cell-surface markers
HSCs	Lin- CD34+ CD38- CD45RA- CD90+ CD49f+/-
MPPs	Lin- CD34+ CD38- CD90- CD45RA- CD49f- CD10- CD7-
CMPs	Lin- CD34+ CD38+ CD90- CD45RA- CD49f- CD10- CD7-
MEPs	Lin- CD34+ CD38+ CD90- CD45RA- CD49f- CD10- CD7- CD123-
GMPs	Lin- CD34+ CD38+ CD90- CD45RA+ CD49f- CD10- CD7- CD123+/-
CLPs	Lin-, CD34+ CD38+ CD90- CD45RA+ CD49f - CD10+ CD7-
T cells	CD45+ CD3+
NK cells	CD45+ CD56+
ILCs	Lin+/- CD45+ CD127+
Monocytes	CD34- CD33+ CD14+
Dendritic cells	Lin- CD45+ CD123+
Mast cells	CD45+ CD203c+
Basophils	CD45+ CD203c+ CD63+
Neutrophils	CD45+ CD15+ CD16+
Eosinophils	CD45+ CD15+ CD16-
Erythroid progenitors	CD36+ CD71+
Erythrocytes	CD235a+
Immature MKs	Lin- CD34- CD41+ CD61+ CD42-
Mature MKs	Lin- CD34- CD41+ CD61+ CD42+
Pro-B cells	CD45+ CD34+ CD19+ CD10+
Pre-B cells	CD45+ CD34- CD19+ CD10+
Mature B cells	CD45+ CD34- CD19+ CD10-
Endothelial cells	CD31+ CD144+ CD309+

Supplementary table 2 – Phenotypic cell types per organ, related to Figure 1.

Phenotypic cell type	Femur	Hip	Liver	Total
Committed progenitors (Lin-CD34+CD38+)	291	128	257	676
Non-committed progenitors (Lin-CD34+CD38-)	262	114	187	563
Basophils	76	0	47	123
CLPs	33	0	41	74
CMPs	130	12	76	218
CD62L-CD52-CD114-CD125-CD117+	79	0	86	165
Dendritic cells	85	0	87	172
Erythroid progenitors	126	0	0	126
Endothelial cells	35	0	29	64
Eosinophils	20	0	29	49
Erythrocytes	22	0	39	61
GMPs	117	7	88	212
HSCs	50	8	67	125
ILCs	75	0	83	158
MEPs	131	22	95	248
Immature MKs	48	0	0	48
Mature MKs	31	0	0	31
Monocytes	45	0	43	88
MPPs	121	38	111	270
Bone marrow cells	137	0	0	137
Mast cells	41	0	0	41
Neutrophils	39	0	31	70
NK-cell	74	0	0	74
CD-REF cells	500	0	0	500
T cells	79	0	0	79
Mature B cells	20	0	22	42
Pre-B cells	34	0	26	60
Pro-B cells	26	0	4	30
Total	2727	329	1448	4504

Supplementary table 3 – scRNA-Seq samples, related to the STAR Methods section.

No.	Gates	Min #counts	Max #counts	Min #genes	Max #genes	Max %mito
1	Non-committed progenitors<br>Committed progenitors	10,000	1,750,000	200	7,500	40
2	Non-committed progenitors<br>Committed progenitors<br>HSCs	10,000	2,000,000	200	7,500	40
3	Non-committed progenitors<br>Committed progenitors<br>HSCs<br>MPPs<br>CMPs<br>GMPs<br>MEPs	10,000	1,500,000	200	7,500	40
4	Non-committed progenitors<br>Committed progenitors<br>HSCs<br>MPPs<br>CMPs<br>GMPs<br>MEPs	10,000	2,750,000	200	9,000	40
5	Immature MKs<br>Mature MKs	10,000	1,750,000	200	9,000	40
6	Non-committed progenitors<br>Committed progenitors<br>HSCs<br>MPPs<br>CMPs<br>GMPs<br>MEPs	10,000	1,500,000	350	7,500	40
7	Bone marrow cells	400	1,750,000	200	7,500	60
8	Endothelial cells<br>Pro-B cells<br>Pre-B cells<br>Mature B	400	1,500,000	200	7,500	60
9	CLPs<br>GMPs<br>Monocytes	1,000	2,000,000	200	7,500	40
10	CD-REF cells	1,000	550,000	200	6,000	40
11	ILCs<br>NK cells<br>T cells	2,000	1,500,000	200	10,000	40
12	Neutrophils<br>Eosinophils<br>Erythrocytes	2,000	1,500,000	200	6,000	40
13	Dendritic cells<br>Mast cells<br>Basophils	2,000	1,500,000	200	7,500	40
14	Erythroid progenitors	500	3,000,000	100	4,500	40
15	CD62L- CD52- CD114-<br>CD125- CD117+	2,000	1,000,000	1,200	7,000	40
